# Comparative Genomics Supports Ecologically Induced Selection as a Putative Driver of Banded Penguin Diversification

**DOI:** 10.1093/molbev/msae166

**Published:** 2024-08-16

**Authors:** Fabiola León, Eduardo Pizarro, Daly Noll, Luis R Pertierra, Patricia Parker, Marcela P A Espinaze, Guillermo Luna-Jorquera, Alejandro Simeone, Esteban Frere, Gisele P M Dantas, Robin Cristofari, Omar E Cornejo, Rauri C K Bowie, Juliana A Vianna

**Affiliations:** Pontificia Universidad Católica de Chile, Facultad de Ciencias Biológicas, Instituto para el Desarrollo Sustentable, Santiago, Chile; Millennium Institute Center for Genome Regulation (CRG), Santiago, Chile; Millennium Institute of Biodiversity of Antarctic and Subantarctic Ecosystems (BASE), Santiago, Chile; Millennium Nucleus of Patagonian Limit of Life (LiLi), Santiago, Chile; Pontificia Universidad Católica de Chile, Facultad de Ciencias Biológicas, Instituto para el Desarrollo Sustentable, Santiago, Chile; Millennium Institute Center for Genome Regulation (CRG), Santiago, Chile; Millennium Institute of Biodiversity of Antarctic and Subantarctic Ecosystems (BASE), Santiago, Chile; Millennium Nucleus of Patagonian Limit of Life (LiLi), Santiago, Chile; Pontificia Universidad Católica de Chile, Facultad de Ciencias Biológicas, Instituto para el Desarrollo Sustentable, Santiago, Chile; Millennium Institute Center for Genome Regulation (CRG), Santiago, Chile; Millennium Institute of Biodiversity of Antarctic and Subantarctic Ecosystems (BASE), Santiago, Chile; Millennium Nucleus of Patagonian Limit of Life (LiLi), Santiago, Chile; Millennium Institute of Biodiversity of Antarctic and Subantarctic Ecosystems (BASE), Santiago, Chile; Department of Biogeography and Global Change, Museo Nacional de Ciencias Naturales (MNCN-CSIC), Madrid, Spain; Department of Biology, University of Missouri St. Louis and Saint Louis Zoo, St. Louis, MO 63121-4400, USA; Department of Conservation Ecology and Entomology, Faculty of AgriScience, Stellenbosch University, Stellenbosch 7602, South Africa; Center for Ecology and Sustainable Management of Oceanic Islands (ESMOI), Departamento de Biología Marina, Universidad Católica del Norte, Coquimbo, Chile; Centro de Estudios Avanzados en Zonas Áridas (CEAZA), Universidad Católica del Norte, Coquimbo, Chile; Facultad de Ciencias de la Vida, Universidad Andrés Bello, Departamento de Ecología y Biodiversidad, Santiago, Chile; Centro de Investigaciones de Puerto Deseado, Universidad Nacional de la Patagonia Austral, Puerto Deseado, Argentina; PPG Biologia de Vertebrados, Pontifícia Universidade Católica de Minas Gerais, Belo Horizonte, MG 30535-901, Brazil; Institute of Biotechnology, HiLIFE, University of Helsinki, Helsinki, Finland; Department of Ecology and Evolutionary Biology, University of California, Santa Cruz, CA 95060, USA; Museum of Vertebrate Zoology and Department of Integrative Biology, University of California, Berkeley, CA 94720-3160, USA; Pontificia Universidad Católica de Chile, Facultad de Ciencias Biológicas, Instituto para el Desarrollo Sustentable, Santiago, Chile; Millennium Institute Center for Genome Regulation (CRG), Santiago, Chile; Millennium Institute of Biodiversity of Antarctic and Subantarctic Ecosystems (BASE), Santiago, Chile; Millennium Nucleus of Patagonian Limit of Life (LiLi), Santiago, Chile

**Keywords:** penguin, genomics, diversification, adaptation, ecological niche

## Abstract

The relative importance of genetic drift and local adaptation in facilitating speciation remains unclear. This is particularly true for seabirds, which can disperse over large geographic distances, providing opportunities for intermittent gene flow among distant colonies that span the temperature and salinity gradients of the oceans. Here, we delve into the genomic basis of adaptation and speciation of banded penguins, Galápagos (*Spheniscus mendiculus*), Humboldt (*Spheniscus humboldti*), Magellanic (*Spheniscus magellanicus*), and African penguins (*Spheniscus demersus*), by analyzing 114 genomes from the main 16 breeding colonies. We aim to identify the molecular mechanism and genomic adaptive traits that have facilitated their diversifications. Through positive selection and gene family expansion analyses, we identified candidate genes that may be related to reproductive isolation processes mediated by ecological thermal niche divergence. We recover signals of positive selection on key loci associated with spermatogenesis, especially during the recent peripatric divergence of the Galápagos penguin from the Humboldt penguin. High temperatures in tropical habitats may have favored selection on loci associated with spermatogenesis to maintain sperm viability, leading to reproductive isolation among young species. Our results suggest that genome-wide selection on loci associated with molecular pathways that underpin thermoregulation, osmoregulation, hypoxia, and social behavior appears to have been crucial in local adaptation of banded penguins. Overall, these results contribute to our understanding of how the complexity of biotic, but especially abiotic, factors, along with the high dispersal capabilities of these marine species, may promote both neutral and adaptive lineage divergence even in the presence of gene flow.

## Graphical abstract

**Graphical Abstract:**
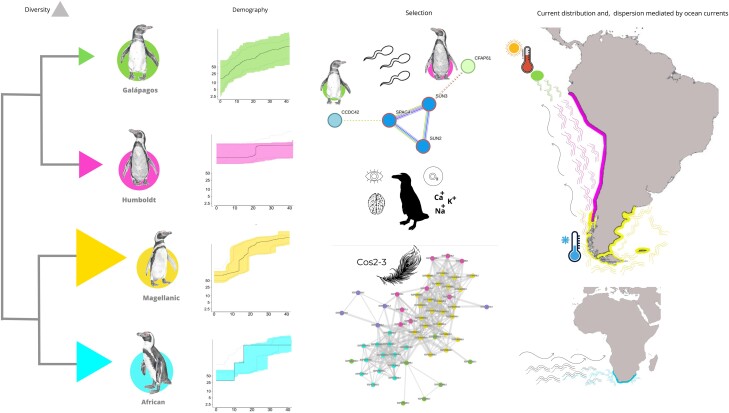

## Introduction

Strong environmental gradients in temperature and salinity across oceanic basins and the geographic isolation of islands are key factors that underpin the remarkable evolutionary radiation of many phenotypically unique plants and animal lineages (Warren et al. 2015). Islands are also hypothesized to have been fundamental to the diversification of several seabird lineages across the globe (e.g. shearwaters, Procellariidae, Obiol et al. 2023; tropicbirds, Phaethontidae, Varela et al. 2024). Given the high dispersal capacity of seabirds with shallow genetic divergence among species being characteristic of several clades (e.g. gulls, Laridae, Sonsthagen et al. 2016; shags, Phalacrocoracidae, Rawlence et al. 2022; skuas, Stercorariidae, Mikkelsen and Weir 2023), it seems that long periods of isolation in allopatry are unlikely to be the only, or even the most important, driver of lineage diversification in seabirds, thereby fundamentally differing from drivers of lineage formation among landbirds (Cai et al. 2020). This raises the question of the relative importance of accumulating genetic differences through genetic drift versus through natural selection (local adaptation) in shaping lineage formation among seabirds. This question is particularly pertinent given that both genetic drift and local adaptation are modulated by the extent of gene flow among populations and lineages (Via 2009). While the extensive movement of seabirds across large geographical ranges facilitates genetic exchange among populations, factors such as oceanic barriers, degree of population isolation, and specialized breeding behaviors (prezygotic barriers) can lead to divergence by restricting gene flow (Welch et al. 2012; Danckwerts et al. 2021; Kersten et al. 2021).

The mechanisms driving the accumulation of differences between populations can be prezygotic (e.g. morphology and breeding behavior) or postzygotic (mechanical incompatibility and infertility), often working synergistically to result in reproductive isolation. During the early stages of speciation, genetic divergence between populations is expected to occur at a few key loci (Via and West 2008; Feder et al. 2012). In the late stages of speciation, genome-wide divergence is likely to be observed due to selection acting on multiple adaptive loci to restrict gene flow (Feder and Nosil 2010). The sex chromosomes are among the genomic regions that undergo rapid differentiation, primarily due to their smaller effective population size that makes selective sweeps more likely. Divergence of sex chromosomes is further influenced by the presence of loci associated with reproduction located on these chromosomes, which can ultimately lead to postzygotic isolation through positive selection acting on these loci (Dufresnes and Crochet 2022).

Banded penguins (genus *Spheniscus*) are exceptional for their ability to have successfully colonized both tropical and temperate latitudes and are one of the youngest lineages among living penguins having originated along the South America coast some 1.8 million years ago (Mya) (Vianna et al. 2020). Four species are recognized in the genus. Three species are distributed along the coast of South America, including the Galápagos (*Spheniscus mendiculus*), Humboldt (*Spheniscus humboldti*), and Magellanic penguins (*Spheniscus magellanicus*), and the African penguin (*Spheniscus demersus*) is distributed along the south and west coasts of South Africa and Namibia. The Galápagos penguin holds the distinction of being the northernmost penguin species, straddling the equator (Garcia-Borboroglu and Boersma 2013). Magellanic penguins range across both the Pacific and Atlantic coasts of South America, while the Humboldt penguin is restricted to the Humboldt Current in the Pacific Ocean.

Here, we evaluate several classic speciation models, often formulated for landbirds, using banded penguins as an exemplar system with which to further our understanding of the speciation process in seabirds. First, we explore support for peripatric speciation between Galápagos and Humboldt penguins, representing an island–continent system. Second, we explore the evidence for parapatric speciation across the range boundary between Humboldt and Magellanic penguins. Third, we explore the extent of speciation in allopatry between the sister species African and Magellanic penguins. We hypothesize that speciation events in banded penguins were driven not only by geographical distance between continents or continent–islands but also by environmental heterogeneity, leading to selection across the genome to facilitate adaptation to local conditions. This is particularly significant given that most extant penguin lineages are adapted to the low water temperatures and high salinity of the sub-Antarctic and Antarctic (Thomas et al. 2020; Vianna et al. 2020; Cole et al. 2022), while banded penguins must navigate a trade-off between inhabiting subtropical and tropical regions and maintaining thermal equilibrium during reproductive seasons, especially in the face of heatwaves and shifts in local salinity levels.

## Results

**Fig. 1.**
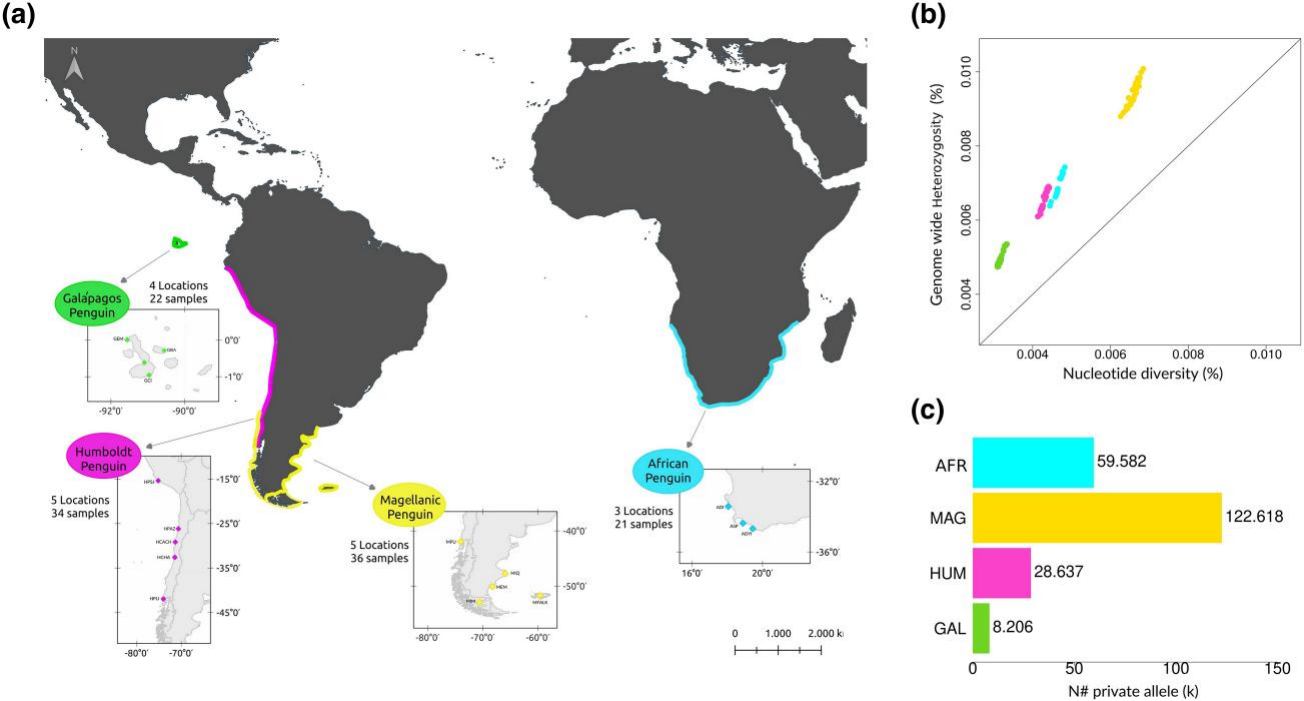
Geographic distribution and genetic diversity of banded penguin species. a) Geographic distribution of the breeding banded penguin colonies sampled in this study with the number of individuals sampled indicated. Color codes correspond to the 4 banded penguin (*Spheniscus*) species. b) Nucleotide diversity vs. genome-wide heterozygosity. c) Number of private alleles restricted to each species: African (AFR), Magellanic (MAG), Humboldt (HUM), and Galápagos Penguin (GAL).

A total of 114 penguin genomes were obtained, covering a range of per-sample depth of coverage values between 3× and 7× (see supplementary figs. S1 and S2 and table S1, Supplementary Material online). On average, 7 individuals from each of the 16 major breeding colonies were sequenced, thereby enabling us to sample from across the geographic range encompassed by each of the 4 banded penguin species (Fig. 1; supplementary table S2, Supplementary Material online).

### Diversity and Diversification

Nucleotide diversity, heterozygosity, and the number of private alleles were lowest in the Galápagos penguin and greatest in the Magellanic penguin (Fig. 1; supplementary figs. S3 and S4 and tables S3 and S4, Supplementary Material online). The percentage of sequence dissimilarity within species was lower in the Galápagos penguin (<0.004%) relative to the other 3 banded penguin species (supplementary fig. S4a, Supplementary Material online). Relatedness coefficients were consistently highest among Galápagos penguins and lowest among Magellanic penguins (supplementary fig. S4b, Supplementary Material online). Tajima's D was positive for all 4 species, indicating a deficit of rare alleles, a result consistent with population contraction (supplementary fig. S4c, Supplementary Material online).

**Fig. 2.**
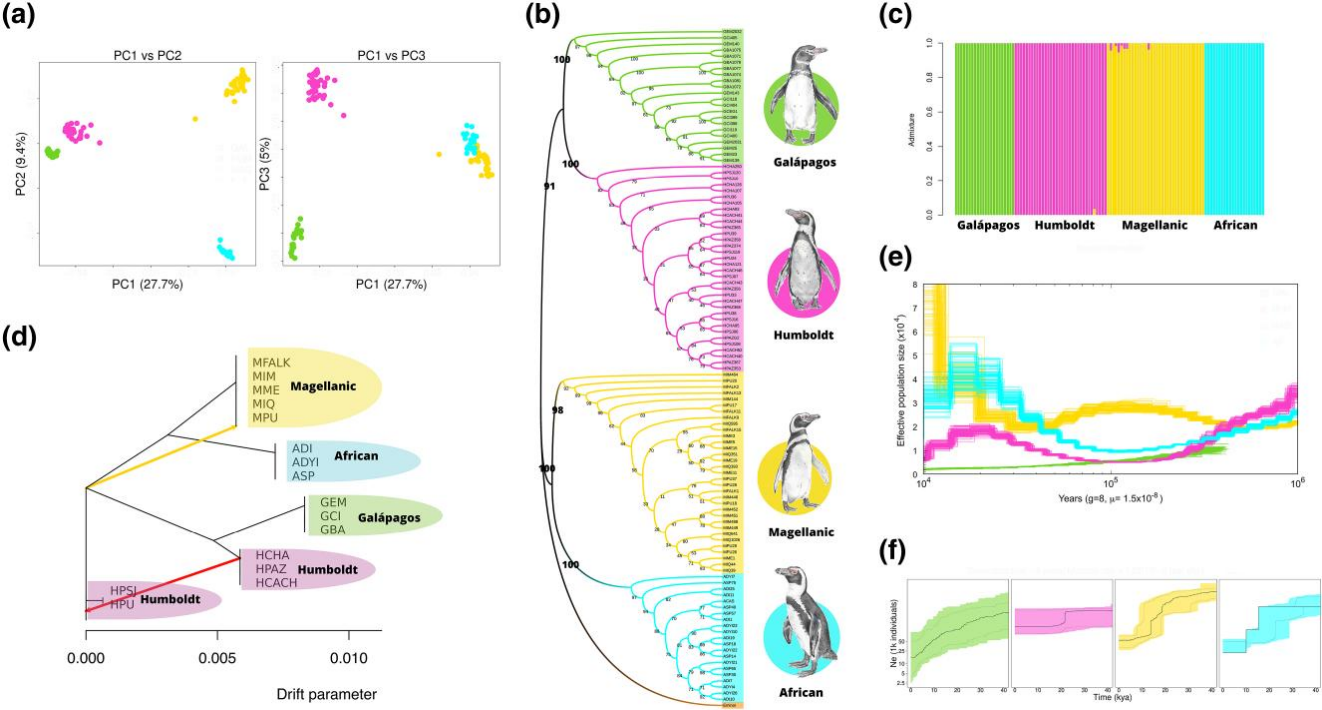
Population structure and demographic history of banded penguins. a) PCA based on genome-wide SNP data. b) Maximum likelihood topology generated with 4,700 UCEs. c) Estimates of admixture (*K* = 4). d) Maximum likelihood tree generated using Treemix, with the most significant ancestral periods of introgression indicated. Note that here we made use of neutral gene regions from whole-genome sequence data from a subset of individuals belonging to each of the breeding colonies. e) Demographic history inferred from the PSMC using a single genome of high coverage (∼30×). f) Demographic inference carried out with the set of populations belonging to the same species with stairway plot 2. Breeding colonies of Galápagos penguin: El Muñeco (GEM), Caleta Iguana (GCI), and Bartolomé (GBA); Humboldt penguin: Punta San JUAN (HPSJ), Pan de Azucar (HPAZ), Isla Chañaral (HCHA), Isla Cachagua (HCACH), and Puñihuil (HPU); Magellanic penguin: Puñihuil (MPU), Isla Magdalena (MIM), Malvinas/Falkland Island (MFALK), Monte Entrance (MME), and Isla Quiroga (MIQ); African penguin: Dassen Island (ADI), Stony Point (ASP), and Dyer Island (ADYI).

We performed a principal component analysis (PCA) with data set 1B (refer to supplementary table S3, Supplementary Material online). The PCA revealed that individuals were clustered by species (Fig. 2a), where the first principal component explained 27.7% of the variance and the second principal component explained 9.4% of the variance. Sex-linked sites show higher divergence between the Magellanic and African penguins than between Humboldt and Galápagos penguins (supplementary figs. S5 and S6, Supplementary Material online). Phylogenetic analyses performed on 4,740 ultraconserved elements (UCE) and 16,966 coding sequences (CDS) each placed the Galápagos and Humboldt penguins as sister species and the African and Magellanic penguins as sister species (Fig. 2b; supplementary fig. S7, Supplementary Material online), supporting previous phylogenomic hypotheses of species relationships (Pan et al. 2019; Vianna et al. 2020; Cole et al. 2022).

Pairwise comparisons of the 2D site frequency spectrum further indicated that the Galápagos and Humboldt penguins are sister species, with the Galápagos and African penguins exhibiting the highest level of sequence divergence compared to the other species pairs examined (supplementary fig. S8, Supplementary Material online). The results from admixture analyses recovered *K* = 4 as the optimal number of clusters. This result corroborates the PCA and phylogenomic analyses, with all 4 species being delimited with little indication of recent interspecific admixture (Fig. 2c). In contrast, the results from Treemix recovered 2 vectors whose placement suggests that gene flow may have occurred over the entire history of the diversification of banded penguins. A vector with low migration weight (close to 0) extends from the ancestral taxon of the Humboldt–Galápagos clade to the Magellanic penguin (Fig. 2d), and there is an intraspecific vector between populations of Humboldt penguin. Results from using the ABBA-BABA test (D-statistic *Z*-score > 3) are consistent with the Treemix results, suggesting that interspecific gene flow over the evolutionary history of banded penguins has occurred between Humboldt and Magellanic penguins (supplementary table S5, Supplementary Material online).

The degree of intraspecific population structure observed among sampled colonies spanning each species distributional range was limited, with levels of genomic differentiation between populations within species (*Fst*) varying between 0.001 and 0.006 (supplementary table S6, Supplementary Material online). PCA, EEMS, and BayesAss also support limited population structure within species (supplementary table S7 and figs. S9 and S10, Supplementary Material online).

### Demographic Inference

Both the pairwise sequentially Markovian coalescent (PSMC) models (Li and Durbin 2011) (Fig. 2e) and the stairway plot 2 models (Liu and Fu 2020) (Fig. 2f) supported past changes in population size for each of the 4 banded penguin species. In the PSMC analysis, the effective population size (*Ne*) of the Galápagos penguin shows a consistent decline toward the present. Humboldt and African penguins reached a low *Ne* point around 100,000 years before present (BP), after which both species started to increase. In contrast to the other species, the Magellanic penguin shows an expansion between 500,000 and 100,000 years BP. Stairway plots indicated that all 4 banded penguin species experienced a putative decline in *Ne* at approximately 20,000 years BP, during the last glacial maximum. However, confidence intervals indicate considerable uncertainty for the Humboldt penguin which may have maintained a stable population size. Magellanic, African, and Galápagos penguins have continued to decline over the past 40,000 years. Demographic inferences of individuals from each sampled population (supplementary fig. S11, Supplementary Material online) are consistent with species-wide inferences of changes in *Ne* (Fig. 2e).

### Detection of Outlier Loci, Gene Family Expansion, and Contraction

**Fig. 3.**
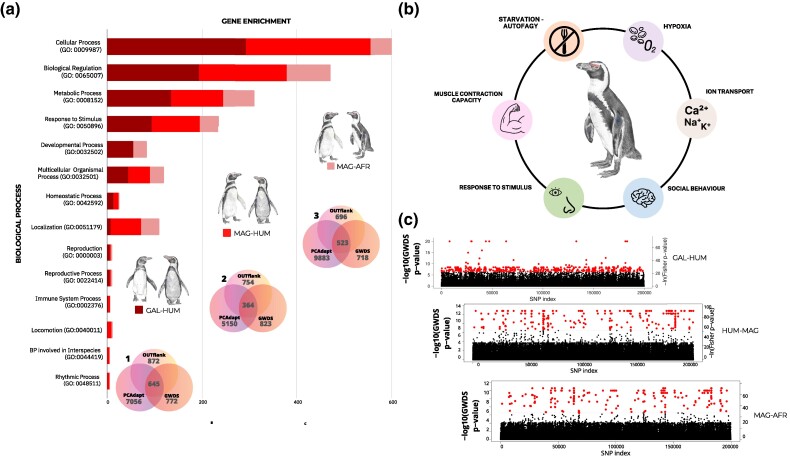
Whole-genome divergent selection between pairwise *Spheniscus* species. a) The primary GO and enrichment analysis conducted in the selection analysis between species (a1 to a3). Venn diagrams illustrate the number of outlier SNPs recovered using OUTFLANK, PCAadpt, and GWDS and their intersection. b) The biological functions inferred to be under selection in all 4 banded penguin species. c) Manhattan plots illustrate the SNPs between species and their degree of differentiation.

A total of 1,532 single nucleotide polymorphisms (SNPs) on autosomal scaffolds (between Galápagos–Humboldt, Humboldt–Magellanic, and Magellanic–African) were identified through species pair comparisons using 3 commonly used methods to detect outlier loci: OUTFLANK (Whitlock and Lotterhos 2015), PCAdapt (Luu et al. 2017), and GWDS (de Jong et al. 2021) (Fig. 3; supplementary table S8, Supplementary Material online). The top 4 enriched biological processes (Fig. 3a) were cellular process (GO:0009987), biological regulation (GO:0065007), metabolic process (GO:0008152), and response to stimulus (GO:0050896). SNPs were distributed across CDSs, genes, messenger RNAs (mRNA), and pseudogenes (supplementary table S8, Supplementary Material online). Depending on the species pair compared, between 364 and 645 SNPs were retrieved by all 3 methods (Fig. 3a1 to a3; supplementary table S8, Supplementary Material online). Signals of selection associated with these biological functions are linked to the ecological habits of the banded penguins, highlighting molecular adaptation to hypoxia, osmoregulation, and visual and olfactory stimuli, as well as muscular development and cognitive capabilities related to social behaviors, memory, and learning (Fig. 3b; supplementary tables S9 and S10, Supplementary Material online). *Fst* of outlier SNPs was lower between Galápagos and Humboldt penguins and higher between Humboldt and Magellanic and Magellanic and African penguins (Fig. 3c). The biological processes showing the highest gene enrichment between Galápagos and Humboldt species (Fig. 3a) were spermatogenesis (GO:0007283), response to heat (GO:0031072), and morphogenetic regulation (GO:0009653; supplementary table S11, Supplementary Material online). Comparison between Galápagos and Humboldt penguin recovered 645 SNPs under selection; 183 (28%) SNPs under divergent selection are involved in spermatogenesis (e.g. *SPAG4*, *ADCY10*, *MROH2B*, *DRC1*, *SUN3*, *ARHGAP24*, *HSD17B11*, *YTHDC1*, *SUN2*, *CFAP61*, and *CCDC42*; Fig. 3b), with several of these loci functionally linked to each other (Fig. 3; supplementary table S9, Supplementary Material online).

A Human Phenotype Ontology analysis revealed enriched phenotypic characteristics associated with the smaller body sizes of Galápagos penguins compared to Humboldt penguins. Among the most prominent phenotypic features were “growth delay,” “microcephaly,” “short digit,” “hypogonadism,” “short nose,” “hypothyroidism,” “short toe,” “short finger,” “short neck,” and “small nail.”

**Fig. 4.**
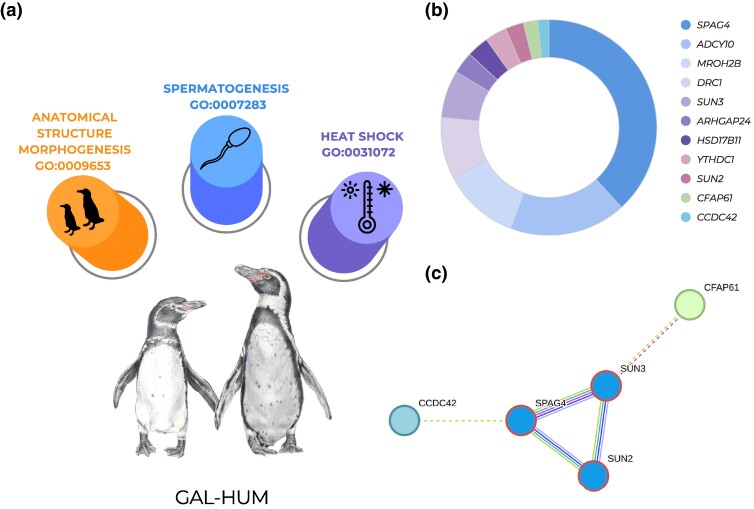
Enrichment results of the SNPs under divergent selection between Galápagos and Humboldt penguins. a) The GO of the most relevant biological processes, along with SNPs under selection, identified throughout the genomes of Galápagos and Humboldt penguins. b) The relative proportion of SNPs under selection, linked to genes associated with functions pertaining to spermatogenesis and sperm motility. c) Associations between spermatogenesis loci revealing functional relationships between genes. Interaction types include direct interactions, gene expression regulation, or participation in shared biochemical pathways.

We used molecular data to identify the sex of each individual revealing a ratio of 52% males to 48% females in our data set. We found 4 sexual scaffolds inferred to be “Z” VULB01013104.1 (*P* = 3.96446519251287e−83), VULB01007854.1 (*P* = 3.54081731216743e−102), VULB01004053.1 (*P* = 7.39397430999582e−136), and VULB01013990.1 (*P* = 3.77227071588691e−65). In males (2 copies of the Z-chromosome), 622 SNPs were recovered across the 4 putative Z-chromosome scaffolds for the Galápagos–Humboldt (60 SNPs), Humboldt–Magellanic (219 SNPs), and Magellanic–African penguin comparisons (343 SNPs) (supplementary fig. S12, Supplementary Material online). The pairwise comparisons indicate a diverse range of biological processes in common, including molecular transducer activity binding (GO:0005488), structural molecule activity (GO:0005198), and catalytic activity (GO:0003824). Among the uniquely enriched biological processes for the Z-chromosome scaffolds in pairwise species comparisons are GAL-HUM molecular function regulator activity (GO:0098772), HUM-MAG cytoskeletal motor activity (GO:0003774), ATP-dependent activity (GO:0140657), and MAG-AFR translation regulator activity (GO:0045182). We confirmed the presence of a gene block on the Z-chromosome (*DCC*, *MEX3C*, *POLI*, *MAPK4*, *PARP8*, *RAB27B*, and *TCF4*) that has been lost in other bird lineages such as Galliformes and passerines (Fig. 4; Friocourt et al. 2017; Patthey et al. 2017).

Restricting the analyses to females (single copy of the Z-chromosome), 828 SNPs were detected to be under selection on the Z-chromosome across the Galápagos–Humboldt (159 SNPs), Humboldt–Magellanic (293 SNPs), and Magellanic–African (376 SNPs) comparisons (supplementary fig. S13, Supplementary Material online). The findings suggest a broad array of shared biological processes, including binding (GO:0005488), structural molecule activity (GO:0005198), molecular function regulator activity (GO:0098772), and catalytic activity (GO:0003824).

**Fig. 5.**
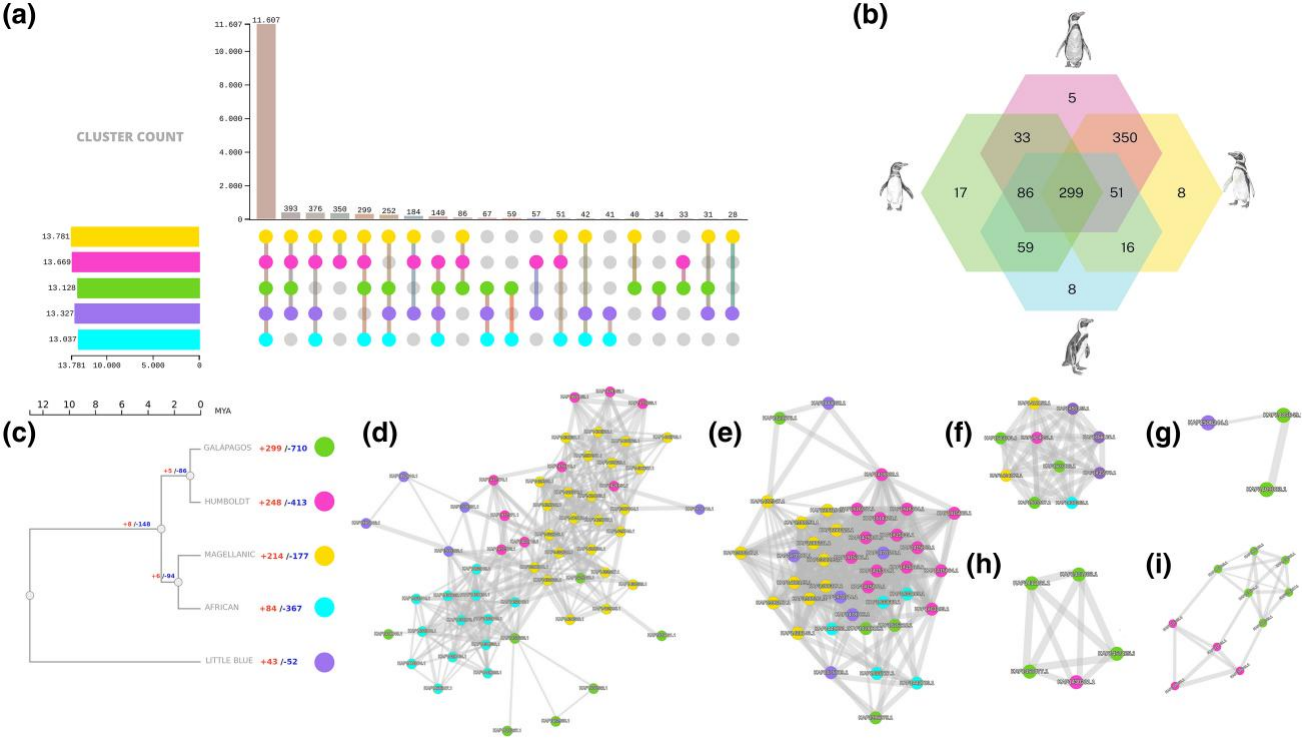
Comparative gene family analyses among banded and little blue penguins. a) Cluster and protein counts of orthogroups found among banded and little blue penguins. b) Cluster and protein count of orthogroups found among banded penguins. c) Maximum likelihood tree based on single-copy genes showing the gain (red) and loss (blue) of genes across banded penguins lineages. d) Feather keratin Cos2-3 family gene expansion. e) Histone H2B family gene expansion. f) The gene family associated with heat stress response includes the heat shock transcription Y-linked gene. (g and h) The adenylate cyclase type 10 and chromodomain Y-like gene are part of the spermatogenesis expanded gene family. i) The gain in the osmoregulation gene family involved sodium channel genes.

OrthoFinder (Emms and Kelly 2019) identified 299 gene families that are shared among all banded penguin species (Fig. 5; supplementary tables S12 to S25, Supplementary Material online), whereas 325 gene families were categorized as single-copy orthogroups including all species. Gene family expansion and contraction were investigated using CAFE5 (Mendes et al. 2021), with the gene birth rate estimated to be 0.00100 when accounting for duplications per gene per million years. Employing this approach, a total of 888 gene families across the 5 species (4 banded species and little penguin as outgroup) were found to have experienced noteworthy expansion, and 1719 experienced contraction events (Fig. 5). The primary biological and molecular processes that have accelerated the rate of evolution among species are associated with intermediate filament bundle assembly (*P*-value 5.05E−27), visceral muscle development (*P*-value 2.62E−09), and nucleosome assembly (*P*-value 0.00; supplementary fig. S14, Supplementary Material online). Several of the expanding gene families among banded penguin species could be involved in biological processes related to the response to environmental stimuli such as olfactory receptors (Fig. 5; supplementary figs. S15a and S16, Supplementary Material online), feather diversification (Fig. 5d; supplementary fig. S15b, Supplementary Material online), osmoregulation, and visual receptors, among others (e.g. histone gene families, Fig. 5e). The Galápagos penguin exhibits 1,009 rapidly evolving gene families, consisting of 299 gene family expansions and 710 contractions. Notably, the expanded gene families in Galápagos penguins were predominantly associated with biological processes such as spermatogenesis (*P*-value 2.9E−04; Fig. 5; supplementary fig. S17, Supplementary Material online), gamete generation (*P*-value 2.14E−08), and reproduction structures (*P*-value 7.7E−32). Additionally, the results suggest an exclusive expansion and accelerated evolutionary rate in the heat shock gene families of the Galápagos penguin and genes involved with spermatogenesis and osmoregulation (Fig. 5f to i). In terms of gene families that have contracted, these genes encoded various proteins such as odorant receptors, keratin proteins, and sodium chloride–channel proteins, among others.

### Comparisons of the Occupancy of Niche Space among Banded Penguin Species

**Fig. 6.**
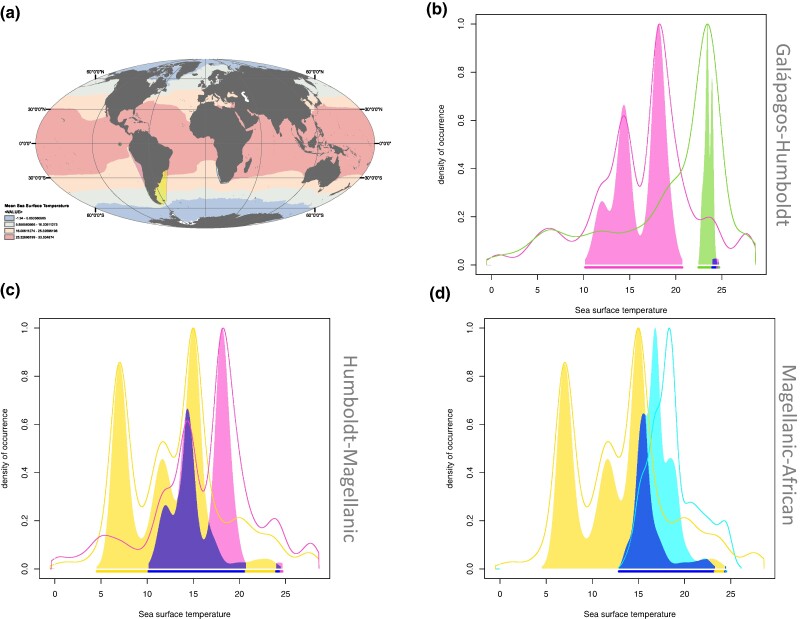
Banded penguin ecological niche overlap. a) Sea surface temperature across the globe, with breeding colony locations where environmental data were obtained. Graphical representation of the extent of niche overlap (in purple and dark blue) in pairwise comparisons between banded species: b) Galápagos penguins (green) vs. Humboldt (pink); c) Humboldt (pink) vs. Magellanic penguins (yellow); d) Magellanic (yellow) vs. African penguins (light blue).

The Galápagos and Humboldt penguins show uneven dynamic niche occupation (Fig. 6; supplementary figs. S18 to S24 and tables S26 to S31, Supplementary Material online). The ecological niche of the Humboldt penguin spans a much higher range of environmental tolerances, whereas the niche occupied by the Galápagos penguin is restricted to the environment of the Galápagos’ Islands (supplementary fig. S18, Supplementary Material online). This results in the Galápagos penguin occupying a novel niche (Schöener’s D overlap of 10%) compared to the Humboldt penguin. In the Galápagos penguin, the species occupancy of islands near the equator represents a unique thermal niche expansion that comprises 99% of its niche space (supplementary table S26, Supplementary Material online). This difference is visualized as a thermal gap in Fig. 6 showing that the colonization of the Galápagos Islands represents a steep transition from its sister species, the Humboldt penguin, by being adapted to seawater that is on average 2 to 4 degrees warmer than the waters occupied by the Humboldt penguin (supplementary fig. S18, Supplementary Material online). Differences observed in the chlorophyll content of the sea are not as pronounced, with 25.6% overlap in trophic levels between Galápagos and Humboldt penguins (supplementary table S27, Supplementary Material online).

Magellanic and Humboldt penguins represent the only pair of banded penguin species that have a partially sympatric range, coexisting along the Southern Pacific coastline of South America. Consequently, their macroecological niche overlap is the highest among banded penguins, reaching 53%, and their thermal niche overlap is 55% comprising nearly 100% of the niche stability (retained niche space) of Humboldt over Magellanic penguins. Thus, Humboldt penguins do not occupy any unique niche thermal space with respect to Magellanic penguins (Fig. 6; supplementary table S5, Supplementary Material online). The unique part of the Magellanic penguin thermal niche (some 37%) can be attributed to the cold waters (down to 5 °C) of the southernmost parts of its range, where Humboldt penguins do not reach. The higher thermal limit of Humboldt and Magellanic penguins is remarkably similar, showing a high degree of niche conservatism, with neither species occupying waters >20 °C suggesting this isotherm may be an important ecophysiological barrier (table S26, Supplementary Material online). The salinity and chlorophyll levels encompassed by the distributional ranges of Humboldt and Magellanic penguins overlap (50% and 74%; supplementary figs. S19 and S20, Supplementary Material online) with a small expansion of the niche for Magellanic penguins (12% and 20%; supplementary fig. S21 and tables S27 to S29, Supplementary Material online).

Magellanic and African penguins consistently exhibit a high degree of niche overlap with the Humboldt penguin, with percentages of 53% and 32%, respectively. Additionally, they share an 18% overlap with each other. The thermal niche, salinity, and chlorophyll levels of the niche space occupied by these species remain consistent, indicating a strong niche conservatism in environmental conditions (supplementary figs. S22 to S24 and tables S26 to S29, Supplementary Material online).

We also recovered niche differences among populations within species that are consistent with the observation of 2 genetic clusters obtained by EEMS for Galápagos, Humboldt, and Magellanic penguins (supplementary figs. S22 to S24 and tables S30 and S31, Supplementary Material online).

The results of the redundancy analysis (RDA) reveal a statistically significant association of a set of environmental and genetic variation among banded penguin species (supplementary figs. S25 and S26 and tables S26 to S29, Supplementary Material online). These results from an RDA suggest that Galápagos penguins tend to be positively associated with the average speed of present ocean currents (VELO) and the annual mean temperature variable (BIO1), while Magellanic penguin tends to be positively correlated with chlorophyll levels in the ocean (CHLO).

## Discussion

Young species assemblages that occur across environmental gradients provide excellent systems to investigate molecular and ecological mechanisms that drive local adaptation. Changes in climate across the southern oceans from the Pleistocene to the present likely led to the current distribution of banded penguins (Vianna et al. 2020). Our results indicated that gene flow following postglacial range expansion appears to have facilitated genetic homogenization of allelic diversity within species, leading to minimal spatial genetic structure within each banded penguin species. Further, our results indicate that both genetic drift among geographically isolated species and local adaptation of populations across ecological gradients have facilitated the diversification of banded penguins and helped to maintain species boundaries in these highly mobile seabirds.

Our analyses suggest that origins of the Galápagos penguin are consistent with the expectations of peripatric speciation. The demographic inference of Galápagos penguins indicated a low effective population size over several hundred thousand years, which is indicative of a small founding population with genetic drift leading to the loss of low-frequency alleles. We hypothesize that Galápagos penguins colonized the Galápagos Islands long after the origin and establishment of the cold Humboldt Current (between the Neogene and the climatic fluctuations of the Quaternary; Thiel et al. 2007) and likely followed the northerly direction of the Humboldt Current and became isolated in the Galápagos Islands after a rare open ocean dispersal event.

Glacial cycles likely significantly disrupted the Humboldt Current due to the presence of ice sheets along the Southern Pacific coast of South America as suggested by geological evidence (Rabassa et al. 2000) and effective population size reduction in other marine species (Pardo-Gandarillas et al. 2018; Dantas et al. 2019; Weinberger et al. 2021). Effective population size of endemic species reliant on one of the world's largest upwelling systems, such as the Humboldt penguin, appears to have been impacted by the drop in sea surface temperature and disruption of the Humboldt Current during glacial cycles. The changes to oceanic conditions altered nutrient supply and thereby likely influenced the reproductive success and population dynamics of coastal breeding populations of penguins (Kim et al. 2002; Thiel et al. 2007; Montecino and Lange 2009; Dantas et al. 2019).

The bathymetric differences between the Atlantic coast compared to the Pacific coast likely resulted in the exposure of the Patagonian Continental Shelf during glacial conditions (Violante et al. 2014). This geographical feature is postulated to have provided emerging nesting sites for intertidal marine vertebrates such as sea lions (Hoffman et al. 2016), rockhopper penguins (Vianna et al. 2020) and Magellanic penguins along the Atlantic coast of South America (Fig. 2) as sea levels fell during the mid-Pleistocene glaciations (Cavallotto et al. 2011).

The limited but ongoing gene flow, the adjacent geographic range with overlapping ecological niches, and ecological niche conservatism support our hypothesis that Humboldt and Magellanic penguins likely diverged in parapatry. Interglacial periods in the South American Austral region would have facilitated secondary contact (Sersic et al. 2011; González-Wevar et al. 2016; Ceballos et al. 2016) and hybridization where species ranges overlap (Simeone et al. 2009; Hibbets et al. 2020; Vianna et al. 2020). Our findings reveal that while such hybridization is infrequent, it has likely persisted for thousands of years, with directional gene flow from Humboldt to Magellanic penguins (Fig. 2d). This result is consistent with the emerging viewpoint that admixture between different lineages is common and widespread in recently diverged species (Richards et al. 2019), highlighting the relevance of speciation with gene flow as an important mechanism of lineage divergence in seabirds.

Previous studies indicate that South America served as the point of origin for banded penguins (*Spheniscus*; Vianna et al. 2020), with the Benguela current playing a crucial role in facilitating dispersal between South America and Africa, thereby promoting speciation through allopatry during the Pleistocene. Our results suggest that after penguins reached Southern Africa, African penguins experienced decreases in effective population size during glacial periods, which may have impacted reproductive success during adverse climatic periods (Barrable et al. 2002; Quick et al. 2022).

The analyses investigating selection across the genome suggest that outlier SNPs, encompassing diverse gene functions, may be undergoing positive or purifying selection in banded penguins. These include loci associated with biological processes related to hypoxia, osmoregulation, and sensory perception, which could be advantageous for diving birds, enabling penguins to detect prey within deep and dimly lit waters. These genetic adaptations appear to align with patterns observed for several seabird species (Pincemy et al. 2009; Coffin et al. 2011; Höglund et al. 2019; Mendelson and Safran 2021; Cole et al. 2022; Sin et al. 2022). Additionally, positive selection and gene family expansion for loci associated with environmental stimuli, such as olfactory and visual receptors, feather keratin, and inner ear balance, suggest the potential reliance of penguins on a combination of sensory cues to navigate their environments and locate food sources, although further research is warranted to confirm these hypotheses.

According to our outlier analyses, genetic variants at specific positions within the genes *SPAG4*, *ADCY10*, *SUN3*, *MROH2B*, and *DRC1*—known for their roles in spermatogenesis, sperm motility, and resilience to heat shock (Frohnert et al. 2011; Calvi et al. 2015; Kenigsberg et al. 2017; Yang et al. 2018; Akbari et al. 2019; Pereira et al. 2019; Zhang et al. 2021)—might play a crucial role in upholding species boundaries between Galápagos and Humboldt penguins. Several gametogenesis genes tend to evolve rapidly, are under positive selection (Swanson et al. 2003; Dean et al. 2008; Kopania et al. 2022), and have been described as “speciation genes” for some model species of birds (Irwin 2018), mammals (Mihola et al. 2009), and insects (Orr et al. 2004; Presgraves 2008). Our results suggest that the signals of selection on loci associated with gametogenesis have been stronger and more frequent in island endemic species such as the Galápagos penguin.

Environmental temperature could have shaped how spermatogenesis genes accumulate genomic differences among populations (Tao et al. 2007; Qvarnström et al. 2016). For instance, male infertility has been related to high temperatures that deform and kill sperm cells (Cai et al. 2021; Schou et al. 2021; Gao et al. 2022). Galápagos penguins experienced the highest maximum sea surface temperatures during the Pleistocene glaciations relative to the remaining banded penguin species (Lyle et al. 1992; Liu and Herbert 2004; Vianna et al. 2020). Thus, selection on alleles at loci associated with spermatogenesis may have been necessary to maintain fertility at higher sea surface temperatures for the Galápagos penguin and thereby facilitated divergence from the Humboldt penguin by increasing the degree of postzygotic isolation in this young species pair. Results from our RDA analyses suggest that currently, annual mean temperatures tend to be positively associated with genotype traits in Galápagos penguins. These findings, coupled with those from the ecological niche analyses, suggest that niche divergence related to temperature, along with signals of selection in genes associated with spermatogenesis, may have been key factors in the ecological speciation of Galápagos penguins. Furthermore, our results suggest that species boundaries are being reinforced by the apparent current absence of gene flow between Galápagos and Humboldt penguins. The absence of gene flow is most likely due to the presence of a thermal isolation barrier (Fig. 6), that Humboldt penguins do not cross despite their ability to travel long distances at sea.

The functional enrichment analysis using the Human Phenotype Ontology database suggests that selection is also acting on loci associated with morphogenesis between Galápagos and Humboldt penguins, particularly in how anatomical structures are generated and organized (supplementary table S11, Supplementary Material online). A reduction in body size may be related to the “island syndrome,” a term that refers to the rapid evolution of reduced dispersal capacity and smaller body sizes associated with high environmental temperatures and dampened temperate extremes on islands (Pörtner and Farrell 2008). This effect is particularly pronounced in amphibious seabirds such as penguins, impacting their ability to achieve thermoneutrality on land and the metabolic costs associated with heat generation while in the sea (Stahel and Nicol 1982; Bevan et al. 2002; Fahlman et al. 2005). Thermal body homeostasis in penguins is influenced by factors such as the high thermal conductivity and specific heat capacity of water during dives, which far exceeds that of air (Stahel and Nicol 1982). Heat stress can also result from extended periods on land during breeding and molting (Wilson and Wilson 1990; Roberts 2016). Notably, body size is crucial for coping with cold stress, governed by the surface area-to-volume ratio (Oswald and Arnold 2012). This factor may restrict certain species, like the little penguin, to temperate regions (Stahel and Nicol 1982) and potentially also be influencing the distribution of Galápagos penguins. Penguins employ diverse mechanisms to counteract heat loss, including dense packing of feathers with no space between feather tracts, layers of blubber, and reduced blood flow to the exposed appendages, especially in response to cold water in upwelling zones (Stonehouse 1967). However, these adaptations present challenges in dissipating heat within burrows, potentially causing thermal stress for banded penguins on land (Boersma and Rebstock 2014; Holt and Boersma 2022; Shaun and Pichegru 2023; Welman and Pichegru 2023). This is particularly pertinent for Galápagos penguins, which may encounter air temperatures exceeding 40 °C (Boersma 1976; Kemper et al. 2007; Boersma and Rebstock 2014).

Based on our findings, we emphasize the importance of identifying the presence of repeated gene blocks on Z sex chromosomes. These blocks, comprised of 12 genes each, have reportedly been lost due to chromosomal rearrangement in other bird lineages and could have significant implications for adaptation and survival in species that retain them (Friocourt et al. 2017; Patthey et al. 2017). Our results suggest that these gene blocks may be under selection in banded penguins, despite the strong purifying selection operating on sex chromosomes. However, caution should be exercised when interpreting signals of selection associated with the sex chromosomes, as faster genetic drift due to the smaller effective population size of sex chromosomes may lead to false signatures of selection (Dean et al. 2015).

Positive selection and gene family expansion for loci associated with the response to environmental stimuli, such as those involved in olfactory and visual receptors, feather keratin, and inner ear balance, suggest that foraging penguins rely on a combination of olfactory, visual, integument, and mechanoreceptors to effectively navigate their environments and locate upwelling waters rich in food sources. While visual perception has long been recognized as crucial for avian foraging, recent research has shed light on the equally significant role of olfactory receptors in Procellariiformes (Sin et al. 2022) and penguins (Coffin et al. 2011) in facilitating foraging. Visual receptors in birds are vital for detecting colors, shapes, and movement of prey when foraging in low-light environments such as underwater, as well as for identifying and avoiding predators such as seals (Höglund et al. 2019, Cole et al. 2022). Differences in visual acuity, feather density and coloration (plumage), and facial patterning could play a role in species-specific mate choice (Pincemy et al. 2009) through sexual selection and thereby serve as important mechanisms in the formation and maintenance of prezygotic reproductive isolation among these young species (Mendelson and Safran 2021). Olfactory receptors enhance avian foraging by allowing birds to detect a variety of odors associated with food availability and also could facilitate the homing ability of seabirds, enabling them to navigate back to their breeding colonies (Silva et al. 2020; Bonadonna and Gagliardo 2021).

## Conclusions

Our research revealed a spectrum of genetic divergence among banded penguin species, characterized by differing degrees of shared genetic variation between lineage pairs. These results suggest recent isolation and independent evolutionary trajectories in diverse environments for the 4 species, with support for peripatric, parapatry, and allopatric models of speciation. Isolation and divergence in temperature niches between Galápagos and Humboldt penguins may have expedited the speciation process between these evolutionary young lineages, potentially through selection acting on genes associated with prezygotic reproductive isolation. Signatures of selection acting on loci associated with hypoxia, osmoregulation, social behavior, responses to external environmental stimuli, thermoregulation, autophagy, and starvation appear to be shared traits under selection in banded penguins. Future research should prioritize obtaining highly contiguous genomes (i.e. chromosomes) to determine the importance of structural variation on species adaptation. While we have explored the potential significance of the genes we identified to be under selection and gene family dynamics linked to the putative diversification of banded penguins, a comprehensive understanding of their evolutionary history necessitates incorporation of data from transcriptomic analyses and functional experiments. Such data would significantly improve our understanding of the underlying molecular adaptations that have enabled banded penguins to occupy waters that span from the subantarctic to the equator.

## Materials and Methods

### Sampling

A total of 114 blood samples were collected from 16 breeding colonies of the 4 species of *Spheniscus* penguins: Galápagos, Humboldt, Magellanic, and African penguins (Fig. 1; supplementary table S2, Supplementary Material online). Individuals were captured, and approximately 1 to 1.5 mL of blood was extracted by puncturing the brachial vein or the dorsal aspect of the foot, after which the birds were released. The blood was preserved by adding 96% ethanol, or lysis buffer, or by spotting onto Fast Technology for Analysis of nucleic acids cards.

### DNA Isolation and Genomic Library Preparation

DNA was isolated from blood samples using a phenol-chloroform or salt extraction protocol (Aljanabi and Martinez 1997). The concentration and integrity of the isolated genomic DNA were determined using a Qubit spectrophotometer (Thermo Fisher Scientific) and by 1% agarose gel electrophoresis. Genomic DNA was fragmented using an ultrasonicator. Once fragmented, the DNA was processed for the purpose of constructing paired-end libraries with the Illumina TruSeqNano kit. The genomic fragments obtained were ligated to polyA tails, index adapters, and barcodes. Six cycles of polymerase chain reaction were carried out, and the amplified libraries were bead purified and then quantified with the Qubit. The resulting libraries were sequenced at ∼5× coverage with paired-end 151 base pair reads using an Illumina NovaSeq 6000 S4 platform at the University of California, Berkeley.

### Quality Control

The quality of the raw DNA sequences was accessed using FastQC v0.11.4 (Andrews 2010) before and after read and quality trimming. Demultiplexing and the trimming of lower quality flanking regions were carried out (establishing a quality threshold of 25) using a sliding window of 4:15 with Trimmomatic (Bolger et al. 2014).

### Resequencing and Variant Discovery

Reads were aligned against the little blue penguin *Eudyptula minor novaehollandiae* genome assembly VULB01 SAMN12384878 (accession: PRJNA556735 ID: 556735; Pan et al. 2019) using BWA-MEM (Li and Durbin 2009). Four of the banded penguin genomes, with approximately 31× coverage each (supplementary table S1, Supplementary Material online), were obtained from Vianna et al. (2020). The aligned SAM and BAM files were processed prior to SNP identification with SAMtools (Danecek et al. 2021), Picard Tools, RealignerTargetCreator, and IndelRealigner (Van der Auwera et al. 2013) to remove duplicates, correct relationship pair information, correct unmapped read flags, and obtain overall mapping statistics. We identified variants for each individual using BCFtools mpileup and BCFtools call implemented in BCFtools (Danecek et al. 2021). We used the approach of Nursyifa et al. (2022) to identify scaffolds comprising the sex chromosomes. This method is based on mapping sequence fragments using differences in relative read depth among scaffolds to identify contigs that map to the sex chromosomes. We recovered 4 sex scaffolds that mapped to the Z-chromosome “Z” VULB01013104.1 (*P* = 3.96446519251287e−83), VULB01007854.1 (*P* = 3.54081731216743e−102), VULB01004053.1 (*P* = 7.39397430999582e−136), and VULB01013990.1 (*P* = 3.77227071588691e−65). Variant site calling files (VCF) were filtered by quality, keeping only those with genotype quality equal to or greater than 30 in Phred score, and covered by at least 3 reads and a maximum of 7 reads.

Low coverage genomes (∼5×) were used to estimate population structure, genetic diversity, and for selection scans, whereas both high (4 ∼30 × genomes) and low coverage genomes were used for demographic reconstruction.

The average coverage and average missing data for each individual genome are summarized in the supplementary figs. S1 and S2, Supplementary Material online, respectively. We identified 60,076,142 SNPs, and specific filters were used to optimize each data set for different analyses (supplementary table S3, Supplementary Material online). SNPs located on the scaffolds comprising the sex chromosomes represented 7% of the data set. Mitochondrial scaffolds were excluded from all analyses.

### Population Structure

To characterize intra- and interspecific genetic variability, we estimated genomic diversity using the following estimators: (i) nucleotide diversity (π) estimated as the average number of nucleotide differences per site between 2 DNA sequences within a population (Nei 1975); (ii) heterozygosity; and (iii) Tajima's D that compares 2 estimators of genetic diversity *π* (nucleotide diversity) and *θ* which is the Watterson's estimator of the population mutation rate (Tajima 1989) (supplementary fig. S3, Supplementary Material online). Genome-wide heterozygosity was computed using the formula in the R package sambaR (de Jong et al. 2021).

Here, nH represents the count of heterozygous sites observed for an individual within the SNP data set, nind signifies the number of nonmissing data points for that individual within the SNP data set, nsnps indicates the total number of SNPs in the data set, and ntotal denotes the total number of sequenced sites, encompassing both monomorphic and polymorphic loci.

We used PCA to explore broad genetic affinities among the banded penguin individuals using Plink 1.9 (Purcell et al. 2007) (Fig. 2; supplementary fig. S9, Supplementary Material online). We estimated ancestry coefficients of the different banded penguin individuals at both the species and population level with ADMIXTURE v1.3 (Alexander and Lange 2011). ADMIXTURE uses a cross-validation procedure to select the most probable number of clusters (*K*) that explain the structure of the data (Fig. 2).

The occurrence of admixture among lineages was further investigated using the interspecific SNP data set with Treemix v1.12 (Fig. 2). Treemix models the relationship among the sample populations with the ancestral population using genome-wide allele frequency data and a Gaussian approximation of genetic drift. The optimal *m*-value (*m* = 2) was estimated using the OptM R package (Fitak 2021).

To determine the extent of hybridization and introgression between Humboldt and Magellanic penguins, we used Dsuite (Malinsky et al. 2021) to obtain the D-statistic and f4-ratios. The D-statistic, also known as the ABBA-BABA test, is commonly used to assess evidence of gene flow between 2 taxa. Under this approach, 4 taxa are analyzed, and the ancestral alleles (“A”) and derivatives (“B”) are considered. The allelic patterns “ABBA” and “BABA” occur with the same frequency in a scenario without introgression, while the excess of either of the allelic patterns is considered to be indicative of introgression between 2 taxa and in the test is reflected by the D-statistic being significantly different from 0 (supplementary table S5, Supplementary Material online). The contemporary gene flow rates were estimated using BayesAss3-SNP (Mussmann et al. 2019; supplementary table S7, Supplementary Material online). We set the number of iterations to 1,000,000, the burn-in to 100,000, and the delta values to 0.1.

### Estimation of Effective Migration Surface

We analyzed patterns of gene flow among banded penguin populations in a spatial context to determine how landscape features impact genetic variation. We used EEMS (Petkova et al. 2016) to estimate gene flow across the landscape. We generated a biallelic matrix of genotypes with Plink 1.9 that was then transformed with bed2diff (available from the EEMS GitHub repository) into a genetic differentiation matrix. Each individual was georeferenced, and a habitat polygon was manually constructed with the help of the Google Maps API v3 Tool (http://www.birdtheme.org/useful/v3tool.html). The study area was covered with a dense regular grid composed of triangular demes. EEMS was run independently for each species using the runeems_SNPS script and the default setting for 10 million steps and a 1 million step burn-in with 400 demes. We used the R-scripts described at https://github.com/dipetkov/reemsplots2 to visualize the results.

### Demographic History

We made use of PSMC version 0.6.5-r67 (Li and Durbin 2011) to reconstruct the demographic history of each species over deep time using both the high coverage (Fig. 2) and low coverage genomes (supplementary fig. S11, Supplementary Material online). For the PSMC analysis, we first called variants for each individual. To do so, we used SAMtools version 1.3.1 with HTSlib 1.3.1 and the vcfutils.pl script from BCFtools 1.3.1 with the command “samtools mpileup -C50 -uf ref.fa aln.bam | BCFtools view -c - | vcfutils.pl vcf2fq -d 10 -D 100 | gzip &gt; diploid.fq.gz”. Following the recommendation of the PSMC documentation (https://github.com/lh3/psmc), we used a third of the average read depth as the minimum read depth (-d) and at least twice the average read depth as the maximum read depth (-D) (-d 3 -D 12). The generated consensus fasta file was made through the fq2psmcfa command, and then, PSMC was run with parameters “-N25 -t15 -r5 -p 4 + 25*2 + 4 + 6”. Once we obtained the psmcfa files, sex-linked scaffolds and CDS regions were removed with the seqtk (Li 2012) and seqkit (Shen et al. 2016) tools. Prior to bootstrapping, we carried out the *splitfa* function, which is a tool for splitting a multi-FASTA file into individual files, with each file containing a single sequence, and then, the inference of population size history of each pseudohaploid fasta sequences was made with the *psmc* command. We assumed a nucleotide substitution rate of *m* = 1.91*10^−9^ substitutions/site/year based on the chicken lineage (*Gallus gallus*) multiplied by the generation time of banded penguin species (*g* = 8) as described by Vianna et al. (2020).

To estimate demographic history over more recent time periods (last 40,000 years), we made use of stairway plots v2.0 using the total set of neutral SNPs of all genomes (low and high coverage) for each species. This method makes use of a likelihood approach to determine values that best reproduce the observed SFS and then uses this information to estimate changes in *Ne* through time. We generated the frequency spectra of folded sites through ANGSD realSFS (Korneliussen et al. 2014). Stairway plot 2 was run on the folded SFS with the same mutation rate parameters and generation time estimates as used for the PSMC analyses.

### Genome-Wide Locus Phylogeny

We obtained UCEs and CDSs from the pseudohaploid fasta files. We used BCFtools norm to align BAM reads to the left, to perform the normalization of the indels, and to check if alleles match the reference. Then, a fasta consensus sequence was generated for each individual with BCFtools consensus. We identified missing sites with bedtools genomecov and masked them with bedtools maskfasta. Once such sites were masked, UCE loci were extracted with PhylUCE (Faircloth 2016) using these functions phyluce_probe_run_multiple_lastzs_sqlite and phyluce_probe_slice_sequence_from_genomes. UCEs present in more than 70% of the taxa were retained for analysis. CDS and exon loci were extracted with Gffreads (Pertea and Pertea 2020). Each of the UCEs, CDS, and exon data sets was separately aligned with MAFFT v7.245 (Katoh and Standley 2013). Phylogenetic trees were estimated from the concatenated data obtained with catfasta2phyml.pl https://github.com/nylander/catfasta2phyml using IQTREE v1.5.3 (Minh et al. 2020) which makes use of the maximum likelihood optimality criteria. The nucleotide substitution model used was GTR + G with branch support determined using an ultrafast bootstrap (Minh et al. 2013).

### Detecting Signatures of Selection across the Genome

Given the recent divergence of the banded penguin species from each other, they constitute an ideal clade with which to identify regions of recent genomic differentiation and candidate loci under selection. We compared sister species Galápagos–Humboldt and Magellanic–African through *Fst*-based selection analyses because genetic divergence due to background drift is minimized. We also compared Humboldt–Magellanic penguins due to their recent introgression events. To screen as many SNPs as possible, we filtered the whole genome raw vcf files with the following parameters using vcftools –minQ 30 –minDP 3 –max-missing 1 -min count 2 and no missing data. Outlier analyses were conducted via several R packages, including OUTFLANK, that works to detect unusually high or low levels of genetic differentiation between populations through Fst pairwise genetic differentiation metrics using false discovery rate (FDR) to reduce false positive; PCAadapt, which combines PCA with linear regression; and GWDS that conducts a SNP-by-SNP analysis comparing allele frequencies between pairs of populations in a data set of biallelic SNPs with Bonferroni correction. We conducted an independent selection analyses on autosomes and sex scaffolds. The genomic positions of outlier SNPs were mapped using the reference genome *Eudyptula minor novaehollandiae* GFF file, which enabled the identification of genes, CDSs, mRNA, and other genome regions.

For analysis of selection on the sex scaffolds, we filtered the raw whole genome VCF files using VCFtools with the following parameters: –minQ 30, –minDP 3, –max-missing 1, –min-count 2, and no missing data. Then, we constructed a VCF file containing only the Z sexual scaffolds. After this, we generated separate VCF files for males (2 copies of the Z-chromosome) and females (a single copy of the Z-chromosome) and subsequently performed selection analyses based on *Fst*, similar to those described in the previous section.

We performed functional enrichment analyses including Gene Ontology (GO and Human Phenotype Ontology (HPO) using Uniprot (The UniProt Consortium 2015), PANTHER (Thomas et al. 2022), and g:Profiler (Kolberg et al. 2020). We used Fisher's exact test with FDR correction to compute significance of associations. GO terms in the categories of biological process, molecular function, and cellular component with a FDR of less than 0.05 were considered significantly enriched. We evaluate the functional interactions of proteins encoded by genes using STRING (Szklarczyk et al. 2021).

### Protein Family Evolution Analyses

We used 5 genome protein sequences of penguin species, including, Galápagos SAMN12384884, Humboldt SAMN12384883, Magellanic SAMN12384882, and African SAMN12384881 penguins to identify gene families (Pan et al. 2019). Little penguin was chosen as the outgroup. We employed OrthoFinder v3 (Emms and Kelly 2019) to infer orthogroups identifying a total of 11,607 gene families (Fig. 5a), encompassing a vast repertoire of 64,058 genes when compared to little penguin (supplementary tables S12 to S25, Supplementary Material online). The evolution of gene families (gain and loss) was analyzed using CAFE v5 (Mendes et al. 2021), with the lambda parameter used for calculating birth and death rates. We used single-copy genes to infer an ultrametric tree with FastTree2 (Price et al. 2010) and calibrated with the divergence time (13 Mya) of the most recent common ancestor between little penguin and Banded penguins, obtained from Vianna et al. (2020).

### Ecological Niche Overlap between Species

Macroecological niche overlap analyses were performed with the R package Ecospat following the work of Broennimann et al. (2012) and Di Cola et al. (2017). Schöener D overlap values indicate the degree of superposition of the 2 paired units compared. In addition to the niche D overlap, which is the same to both paired species, the dynamic niche evolution is presented as an indication of the niche differentiation levels of each species pair. The outcomes of these analyses are presented as niche unfilling, stability, and expansion and respectively indicate unfilling: the percentage of niche of the paired species for comparison that was not occupied by the target species of study; stability: the percentage of the niche of the studied species that overlapped between the species pair; and expansion: the percentage of the niche of the studied species that is exclusive to that species relative to the other species in the pairwise comparison. Niche stability and expansion add to 1. Thus, stability and expansion are given as percentages.

To this end, mean sea surface temperature, mean sea surface salinity, and mean sea surface chlorophyll ocean variables were retrieved from the BioOracle 2.0 repository with a spatial resolution of 10 × 10 km. Spatial occurrences for all 4 banded penguin species were retrieved from their records in the sea reported in GBIF. Spatial records were downloaded and filtered with the R package spocc (https://github.com/ropensci/spocc) to match the resolution of the ocean variables considered. Isolated records from distant locations (>5,000 km) to known colonies were discarded since they are considered either errors or vagrant (nonreproductive) individuals. In addition, for 3 of the 4 species, an additional contrast of niche overlap was calculated for the regional subpopulations that showed a high degree of isolation. In the case of the Galápagos penguin, this was done between records of Isabella and Santiago islands. In the case of the Humboldt penguin, breeding populations of this species were broken into north and south subpopulations using as a natural break reflecting a discontinuity in the species distribution around the Atacama corridor. Lastly, Magellanic penguin subpopulations were subdivided between the Atlantic and the Pacific coasts using the Magellan Strait as a discontinuity.

The multidimensional niche comprising all 3 variables integrated was calculated and mapped in a sPCA for all 6 paired combinations of the 4 banded penguin species as well within the 3 regional intraspecific populations of Magellanic, Galápagos, and Humboldt penguins (supplementary fig. S18, Supplementary Material online). The Schöener D overlap index was estimated (supplementary tables S21 to S23, Supplementary Material online). We then performed a sPCA that depicts the full niche space distribution clouds and the contour of the 95% centroid (discontinued lines, supplementary fig. S18, Supplementary Material online). In addition, the extent of individual variable overlap was estimated and graphically mapped (supplementary figs. S18 to S21, Supplementary Material online). The shared niche space (typically named stability) together with the unique niche of the 2 paired species (typically referred to as niche unfilling and niche expansion) for each individual variable was summarized in supplementary tables S25 to S30, Supplementary Material online. The figures show stability in gray and the unique niche space of each species in its respective guiding color. In the case of intraspecific regional subpopulation comparisons, these are represented with contrasting shades of the same color (dark and light, supplementary figs. S22 to S24, Supplementary Material online).

### Genomic Environmental Association

We utilized RDA, a canonical ordination method developed by van den Wollenberg (1977) and Legendre and Legendre (2012), to investigate the variance in response variables. We used only autosomal scaffolds. For interspecific analysis, we use data set 1B, and for intraspecific analysis, we use data set 2A. The resulting vcf file for each set of SNPs files was converted into a lfmm file for input into the population RDA approach. RDA was conducted using a systematic workflow in R, employing various packages such as vegan (Dixon 2003), LEA (Frichot and François 2015), permute (https://github.com/gavinsimpson/permute), and corrplot (Wei et al. 2017).

The climatic characteristics of each of each site were compared across 19 bioclimatic variables extracted with R software packages: terra (Hijmans et al. 2022), raster (https://github.com/rspatial/raster), and rgdal (Bivand et al. 2015) at 30 arc-second resolutions from the CHELSA database (Karger et al. 2017) covering the period 1980 to 2010. The median elevation was obtained from the SRTM4.1 global topography data set (Amatulli et al. 2018). To choose the environmental variables for the analysis, the correlation between variables was examined with matrices and plots. Variables with R-square values under 0.77 were considered as variables with low correlation and were kept for further analysis (supplementary table S32, Supplementary Material online). Genetic and environmental data were inspected for data structure and filtered for missing values. The best subsetting of variables explored in the RDA analysis (i.e. those maximizing the R-square value while being highly significant) comprised present surface chlorophyll mean (Chlo), present surface current velocity mean (Velo), annual mean temperature (BIO1), and annual precipitation (BIO12) (supplementary table S32 and figs. S25 and S26, Supplementary Material online). The RDA was executed with genetic data regressed on the selected environmental variables that were previously standardized.

We examine the relationship between geographical distances, spatial autocorrelation, and environmental variables within a designated study area. By incorporating Moran's I statistic, we identify spatial patterns to assess the environmental drivers influencing spatial distributions. Subsequently, we derived the dbMEM and integrated it as a covariate in the RDA analysis to evaluate the relationship among environmental variables while accounting for geographical distance.

We conducted RDAs among banded penguin species and within species. The results were examined using several analytical approaches including assessments of eigenvalues and adjusted R-squared values, and significance tests were performed with 999 permutations for (i) the RDA model, (ii) the terms of the model (added sequentially), and (iii) the axes of the model (supplementary table S33, Supplementary Material online). Visualization of the RDA outputs was achieved through scatter plots.

## Supplementary Material

msae166_Supplementary_Data

## Data Availability

Banded penguin raw fastq reads and reconstructed genomes have been deposited in the GenBank database (PRJNA939132, BioSample accession numbers SAMN33459127 to SAMN33459236). All scripts are available at GitHub (https://github.com/lafabi). All other data needed are provided in either the main text or in the Supplementary material.
