# Supplementary material for: Comparative Genomics Supports Ecologically Induced Selection as a Putative Driver of Banded Penguin Diversification
Source: Mol Biol Evol. 2024 Aug 16;41(9):msae166. doi: 10.1093/molbev/msae166 (PMC11371425; doi:10.1093/molbev/msae166)
Supplement: msae166_Supplementary_Data [file msae166_supplementary_data.pdf]

Supplementary Materials

Comparative genomics supports ecologically induced selection as a putative driver of banded penguin diversification

Fabiola León[1,2,3,4], Eduardo Pizarro[1,2,3,4], Daly Noll[1,2,3,4], Luis R. Pertierra[3], Patricia Parker[5], Marcela P. A. Espinaze[6], Guillermo Luna-Jorquera[7,8], Alejandro Simeone[9], Esteban Frere[10], Gisele Dantas[11], Robin Cristofari[12], Omar E. Cornejo[13], Rauri C. K. Bowie[14*], Juliana A. Vianna[1, 2, 3,4*]

*Juliana A. Vianna, Pontificia Universidad Católica de Chile, Facultad de Ciencias Biológicas, Departamento de Ecosistemas, Instituto para el Desarrollo Sustentable, Av. Bernardo O'Higgins 340, Santiago, Chile, phone number: 56-223542730, jvianna@uc.cl, and Rauri C.K. Bowie, Museum of Vertebrate Zoology and Department of Integrative Biology, University of California, Berkeley, 3101 Valley Life Science Building, CA 94720-3160, USA. bowie@berkeley.edu.

Supplementary results and discussion

Banded penguin population Structure

The PCA reveals slight variance explained among sampled colonies, ranging from 6.3% in the Humboldt penguin to 9.7% in the Galapagos penguin (supplementary fig S9A). Although admixture analyses among individuals within species do not align with the PCA results (as shown in K = 1, supplementary fig S9B), EEMS also detected slight spatial population differentiation among colonies for all species, except for the African penguin (supplementary fig S9C). For the Galápagos penguin EEMS detected reduced migration rates between the GBA breeding colony on Santiago Island and the other two breeding colonies, El Muñeco (GEM) and Caleta Iguana (GCI) located on neighboring Isabela Island (supplementary fig S9C1). For the Humboldt penguin EEMS detected a corridor with effective migration rates significantly higher than the general average along the central and southern Pacific coast, connecting all colonies except the northernmost colony (HPSJ, supplementary fig S9C2). For Magellanic penguins, EEMS identified a signal of restricted gene flow between breeding colonies situated on the Pacific and Atlantic coasts, but with gene flow homogenizing genetic diversity among colonies restricted to the Pacific or Atlantic coasts of South America (supplementary fig S9C3). The Neighbor-Joining tree assigned most of the individuals from a locality to their own monophyletic group (supplementary fig S10). Analyses performed with BayesAss (Mussmann et al. 2019) detected slightly asymmetric bidirectional gene flow between populations (supplementary table S7).

Regional niche space comparisons within species

We found niche differences (D overlap 5%) that are consistent with the observation of two genetic clusters obtained by EEMS for Galápagos penguins (supplementary fig. S22, supplementary table S30-S31). The Santiago population is centered on approximately 1ºC warmer temperatures than the Isabela Island populations. Specifically, we observed a thermal overlap of 11%, where 69% of the species' thermal niche space is exclusively comprised by the colonies at Isabella, that is, colder sea water conditions are unfilled by the Santiago cluster. The salinity levels (39%) overlap much more between genetic clusters. Hence, the ecophysiological selective pressure is likely to be driven to a greater extent by differences in thermal conditions rather than salinity between islands.

The two genetic clusters of Humboldt populations along the latitudinal gradient show niche differences consistent with the steep thermal gradient along the coast of South America resulting in less than 6% thermal overlap and an overall niche overlap of 9% (supplementary fig. S23). Thus, limited gene flow between northern and southern populations may in part be explained by different environmental conditions. There is a stronger niche overlap (39%) between the two genetic clusters of Magellanic penguins: temperature (49%), chlorophyll (69% overlap), and salinity (41%) (supplementary fig. S24, supplementary table S30, S31). The large area of Magellanic penguin distribution in the Atlantic and the smaller range in the Pacific could indicate displacement by competition from the Humboldt penguin.

At the intraspecific level, results of the Redundancy Analysis (RDA) reveal that GEM and GCI has a tendency to be positively associated with VELO and CHLO and negatively correlated with GBA in Galápagos penguins (supplementary fig. S26, table S32). These same variables are prone to be positively associated with the HPSJ population and negatively correlated with

HPUNH. Annual Mean Precipitation (BIO12) leans toward positively correlated with the MPUNH population, while ADYI tends to be negatively associated with the Mean Diurnal Range (BIO2) variable, and ASP with BIO1 respectively.

Our results suggest that intraspecific population structure is nearly but not completely panmictic, with a subtle isolation by distance and much of the explainable genetic variation associated with latitudinal separation, which is consistent with previous studies (Dantas et al 2019, Nims et al 2008; Dantas et al 2018). The weak intraspecific structure found among penguin colonies could have evolved over time as a consequence of interactions with environmental factors, where gradients in environmental variables, isolation, and genetic drift play a crucial role in molding the unique traits observed among colonies within each species. Colonies residing in diverse abiotic environments have undergone slight genetic differentiation, apparently, as a result of a combination of factors including isolation by resistance (EEMS), influence of associations with environmental variables (RDA), and thermal niche differentiation.

Material and Methods

To characterize intra- and inter-specific genetic variability, we estimated genomic diversity using the following estimators:

1) nucleotide diversity (π)

$$\pi = \frac{\sum_{i=1}^{n-1} \sum_{j=i+1}^{n} dij}{N \times L}$$

where, *n* is the number of sequences in the sample, *dij* is the number of nucleotide differences between sequence *i*i and sequence *j*j, N=2n(n−1) is the number of pairwise comparisons and L is the total number of nucleotide sites compared_(Nei 1975);

2) heterozygosity which was computed using the formula in the R package sambaR (de Jong et al 2021).

$$H_{genome=}\frac{\left(\frac{n_H}{n_{ind}}\times n_{snps}\right)}{n_{total}}$$

3) Tajima's D (Tajima 1989).

$$D=\pi-\theta_{Wpersite}$$

References

Dantas G, Oliveira L, Santos A, Flores M, Melo D, Simeone A, González-Acuña D, Luna-Jorquera G, Le Bohec C, Valdés-Velasquez A, et al. 2019. Uncovering population structure in the Humboldt penguin (*Spheniscus humboldti*) along the Pacific coast at South America. *PLOS ONE* 14:e0215293.

Dantas GPM, Cardoso MG, Marasco ACM, Castro LT, Almeida VS, Rodrigues Santos F, Oliveira LR, Crespo E, Frere E, Milliones A, et al. 2018. Demographic history of the Magellanic Penguin (*Spheniscus magellanicus*) on the Pacific and Atlantic coasts of South America. *Journal of ornithology* 159: 643-655.

de Jong MJ, de Jong JF, Hoelzel AR, Janke A. 2021. SambaR: An R package for fast, easy andc reproducible population-genetic analyses of biallelic SNP data sets. *Molecular Ecology Resources* 21:1369–1379.

Mussmann SM, Douglas MR, Chafin TK, Douglas ME. 2019. BA3-SNPs: Contemporary migration reconfigured in BayesAss for next-generation sequence data. *Methods in Ecology and Evolution* 10:1808–1813.

Nei M. 1975. Molecular population genetics and evolution. Molecular population genetics and evolution. [Internet]. Available from: https://www.cabdirect.org/cabdirect/abstract/19750118335

Nims BD, Vargas FH, Merkel J. & Parker PG. 2008. Low genetic diversity and lack of population structure in the endangered Galápagos penguin (*Spheniscus mendiculus*). *Conservation Genetics* 9:1413-1420.

Tajima F. 1989. Statistical method for testing the neutral mutation hypothesis by DNA polymorphism. *Genetics* 123:585–595.

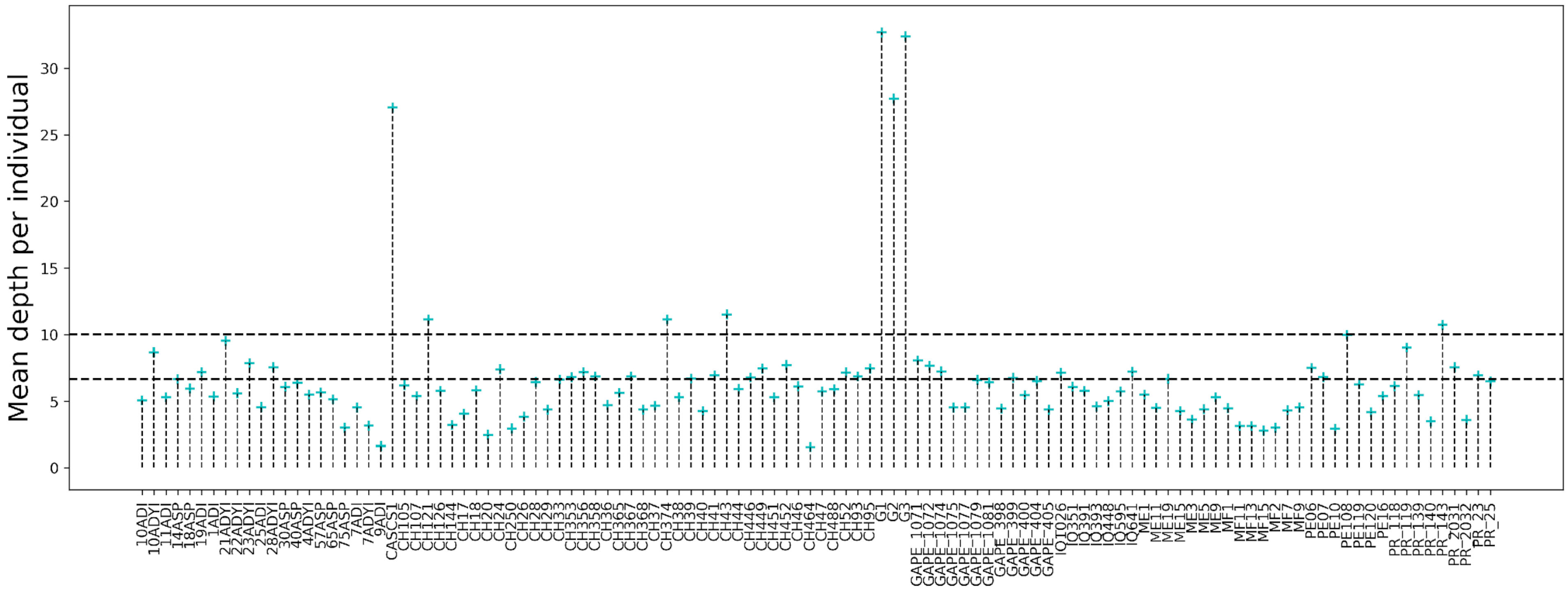

Supplementary Figure 1. Average genomic coverage per individual.

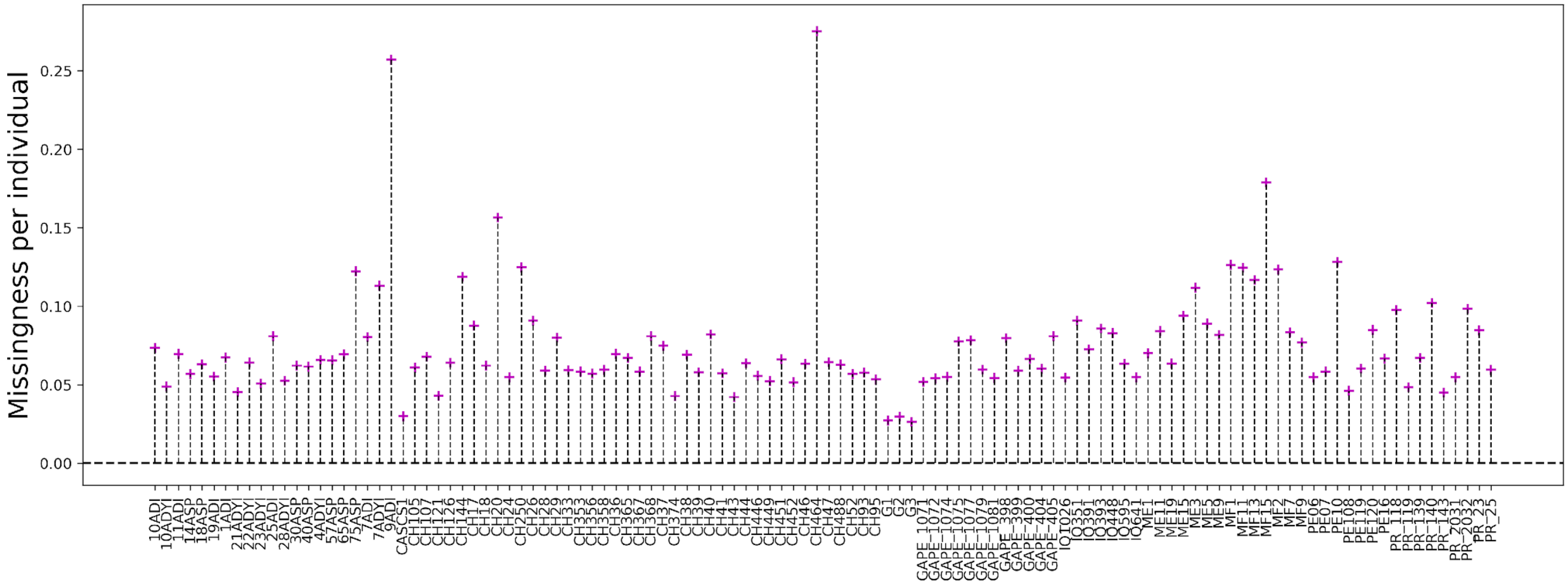

Supplementary Figure 2. Average missing data across the genome for each individual.

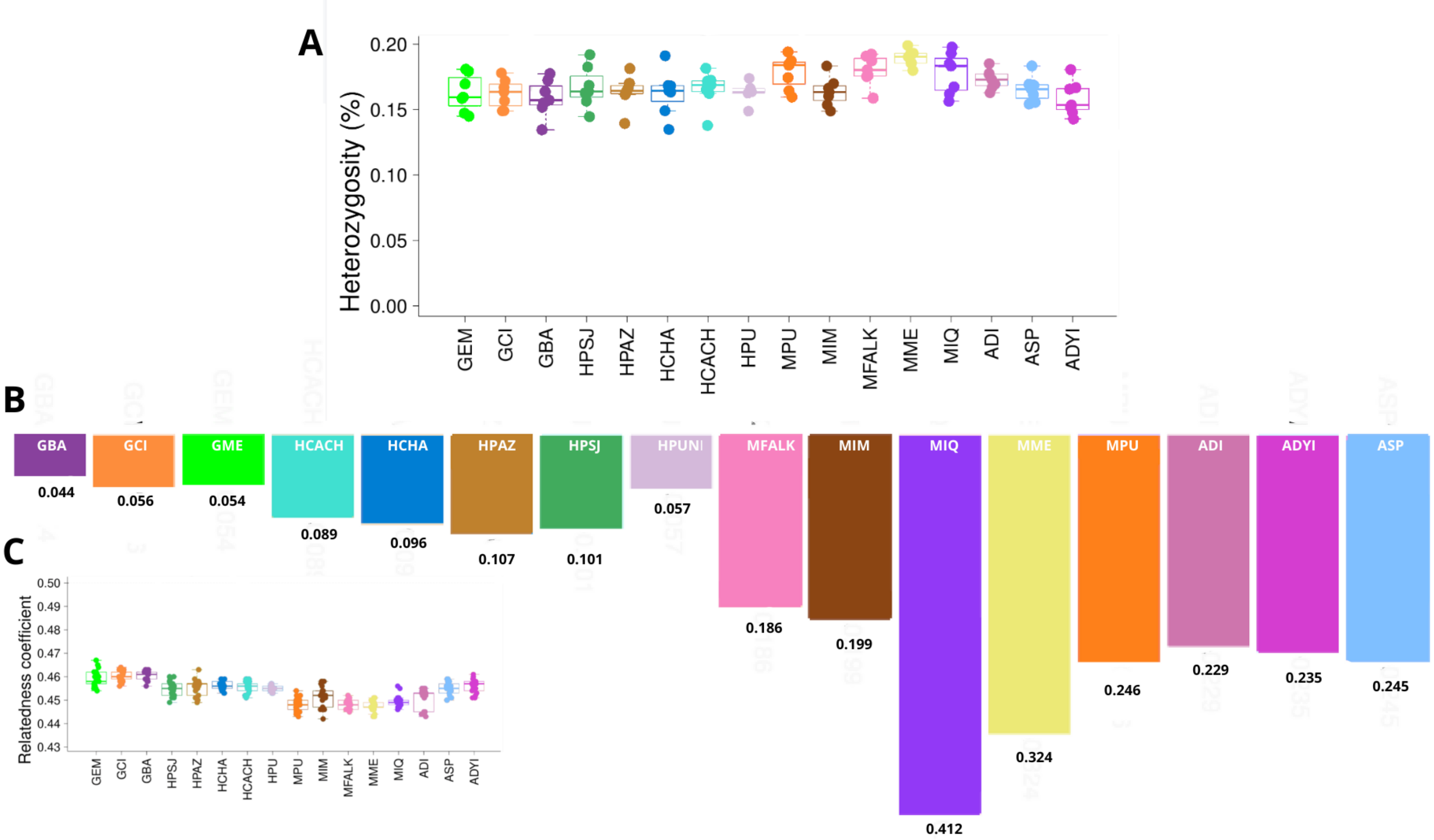
A
Heterozygosity (%)
0.20
0.15
0.10
0.05
0.00
GEM
GCI
GBA
HPSJ
HPAZ
HCHA
HCACH
HPU
MPU
MIM
MFALK
MME
MIQ
ADI
ASP
ADYI
B
GBA
0.044
GCI
0.056
GME
0.054
HCACH
0.089
HCHA
0.096
HPAZ
0.107
HPSJ
0.101
HPUNI
0.057
MFALK
0.186
MIM
0.199
MIQ
0.412
MME
0.324
MPU
0.246
ADI
0.229
ADYI
0.235
ASP
0.245
C
Relatedness coefficient
0.50
0.49
0.48
0.47
0.46
0.45
0.44
0.43
GEM
GCI
GBA
HPSJ
HPAZ
HCHA
HCACH
HPU
MPU
MIM
MFALK
MME
MIQ
ADI
ASP
ADYI

Supplementary Figure 3. Genetic Diversity of each sampled breeding colony of banded penguin. Heterozygosity (A), Private alleles (B), and relatedness coefficients (C). From left to right breeding colonies of Galápagos penguin: El Muñeco (GEM), Caleta Iguana (GCI), Bartolomé (GBA); Humboldt penguin: Punta San JUAN (HPSJ), Pan de Azucar (HPAZ), Isla Chañaral (HCHA), Isla Cachagua (HCACH), and Puñihuil (HPU); Magellanic penguin: Puñihuil (MPU), Isla Magdalena (MIM), Malvinas/Falkland Island (MFALK), Monte Entrance (MME), and Isla Quiroga (MIQ); African penguin: Dassen Island (ADI), Stony Point (ASP), Dyer Island (ADYI).

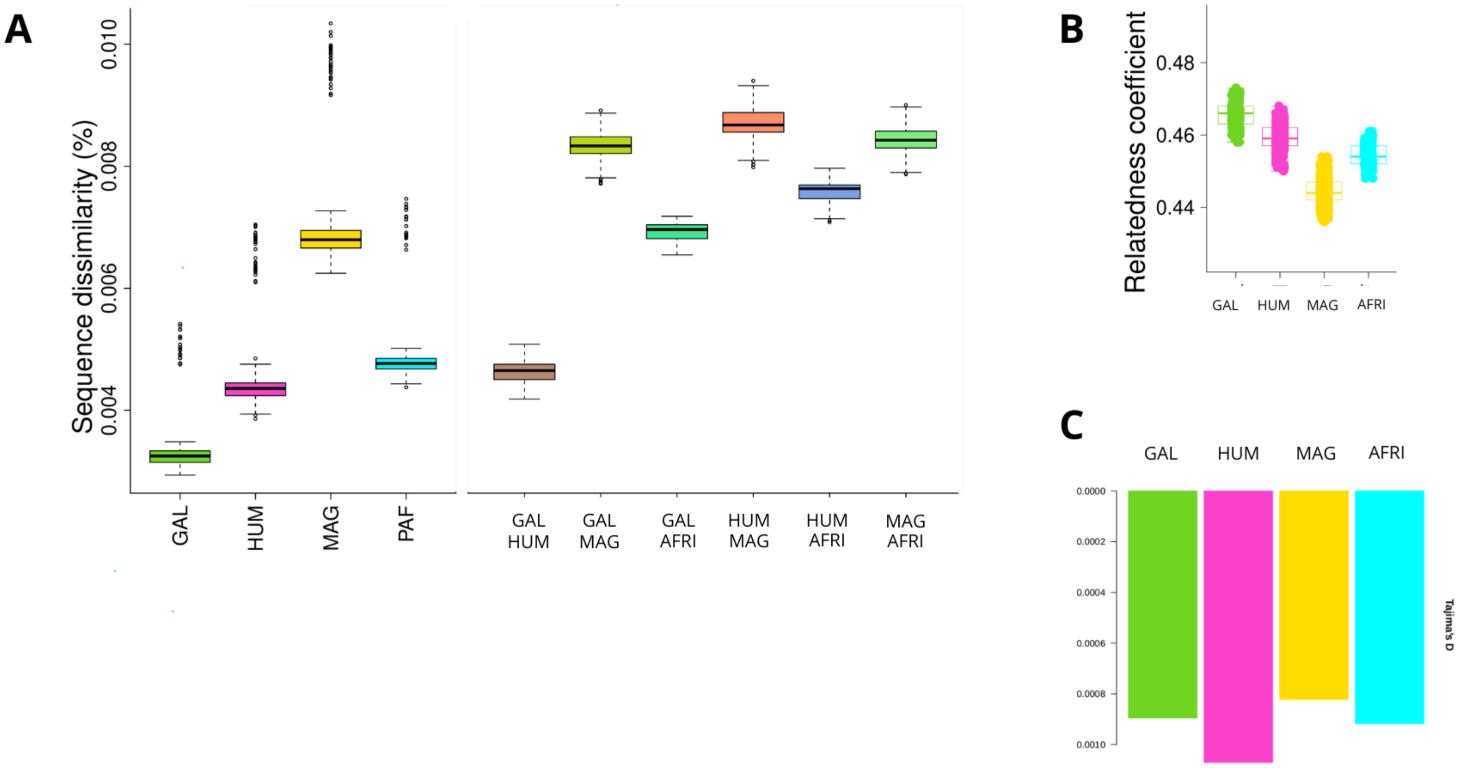

Supplementary Figure 4. Genome-wide genetic diversity of banded penguins. Galápagos penguins (GAL), Humboldt penguins (HUM), Magellanic penguin (MAG), and African penguins (AFRI). (A) Within species, and pairwise between comparison between species dissimilarity by species. (B) Relatedness coefficients among species. (C) Tajima's D index among species; positive values indicate a lack of rare alleles.

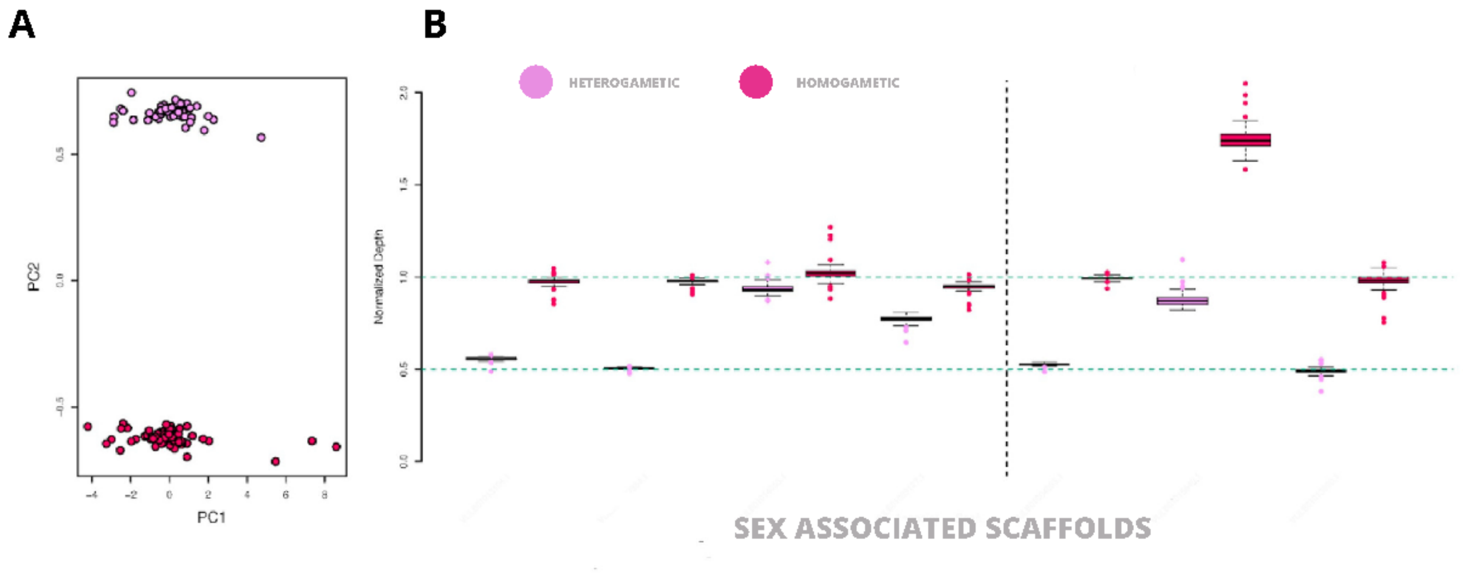

Supplementary Figure 5. Identification of sex and sex-linked scaffolds. The colors in the figure represent the SATC-inferred homogametic (dark red) and heterogametic (pink red) sexes. (A) On the left, a Principal Component Analysis (PCA) plot depicting the normalized depth of coverage across all scaffolds and samples. (B) On the right, a boxplot illustrates the normalized depth of coverage for each sex from the identified sex-linked scaffolds. A vertical dashed line delineates between scaffolds that satisfied both the t-test and ratio-

based threshold criteria (i.e., X/Z-linked, to the left of the line) and those that solely met the t-test criterion (i.e., abnormal depth ratios, to the right of the line).

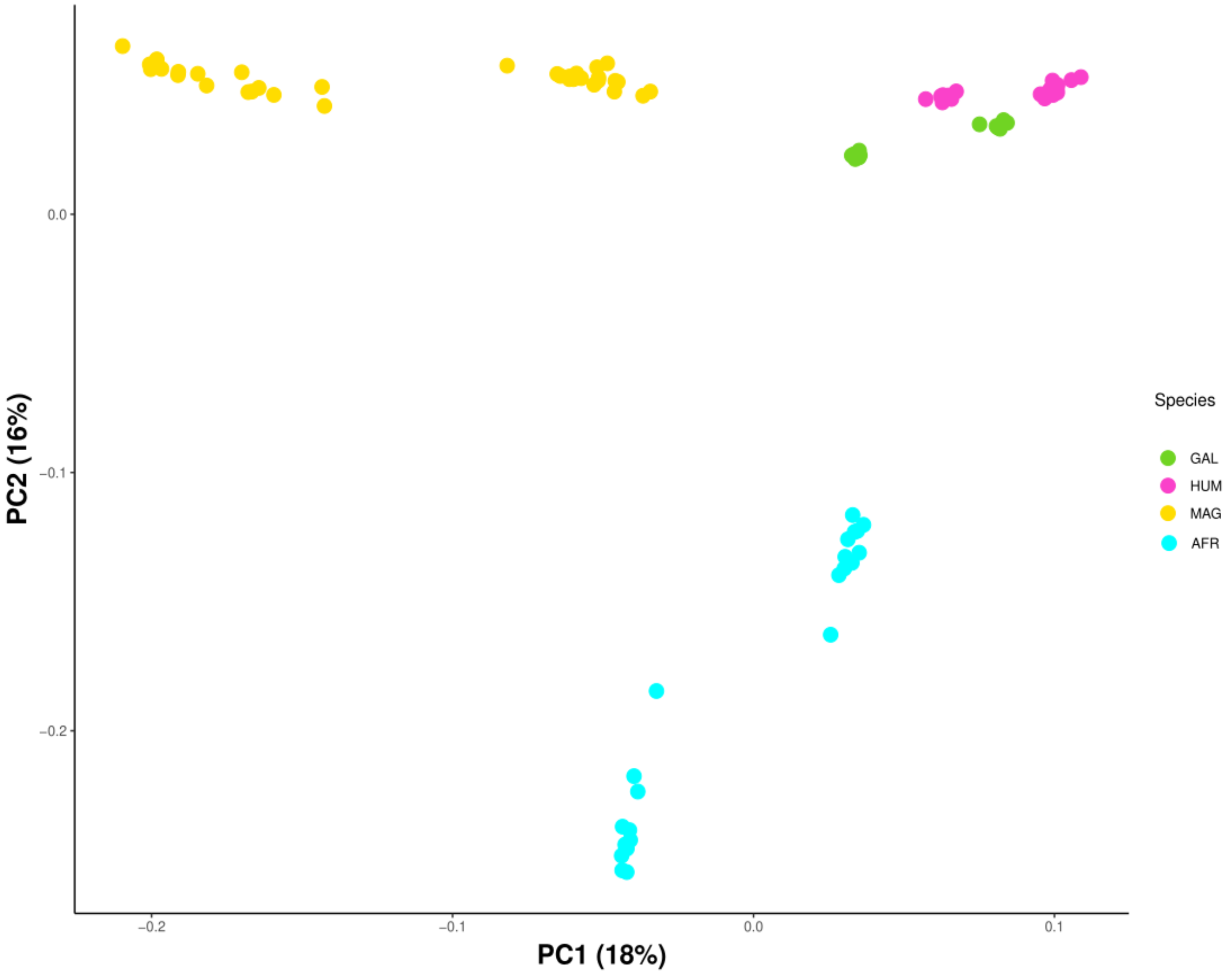

Supplementary Figure 6. Principal Component Analysis of sex-linked scaffolds. SNPs from Galápagos and Humboldt penguins cluster closer to each than to Magellanic and African penguins.

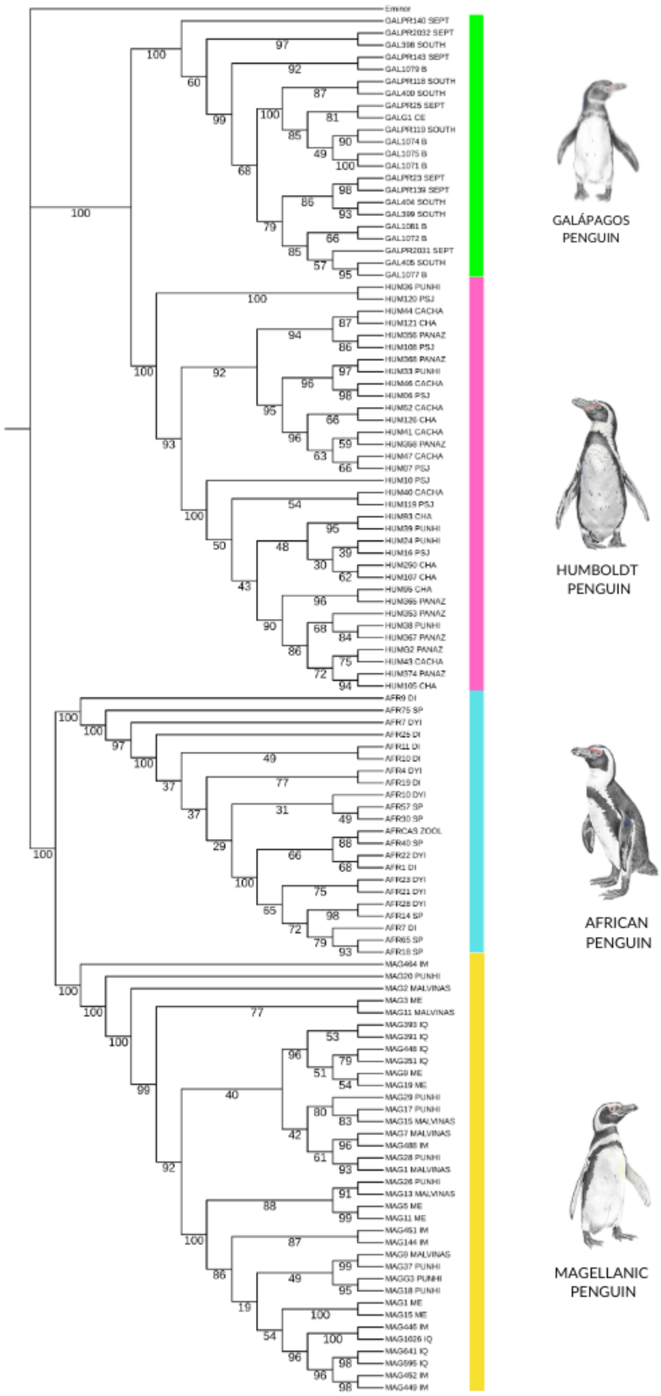

Supplementary Figure 7. Maximum likelihood (ML) topology depicting relationships among the 4 banded penguin species. The ML tree was generated with 16,966 CDS

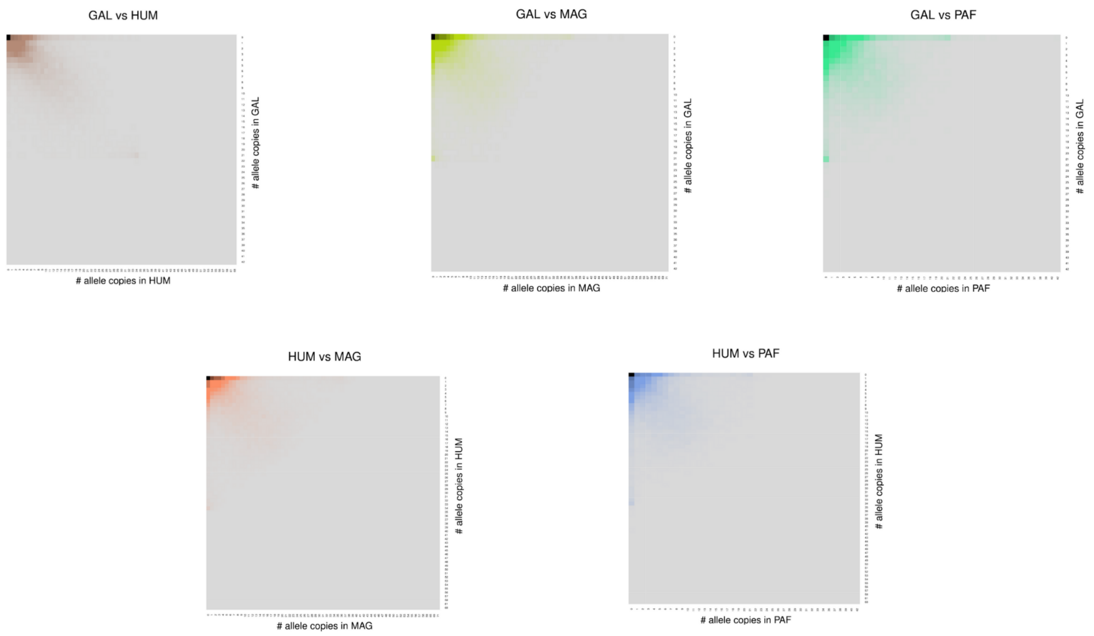

Supplementary Figure 8. Two-dimensional frequency spectrum (2D-SFS) heatmap between banded penguin species. Recent divergences are shown as spectra with a density trend of SNPs that are located along the diagonal. Lineages with more ancestral divergences show spectra with SNP densities that are located towards the X and Y axes indicating opposite evolutionary trends. Each figures indicate the pairwise comparison by GAL (Galápagos), HUM (Humboldt), MAG (Magellanic), PAF (African penguin).

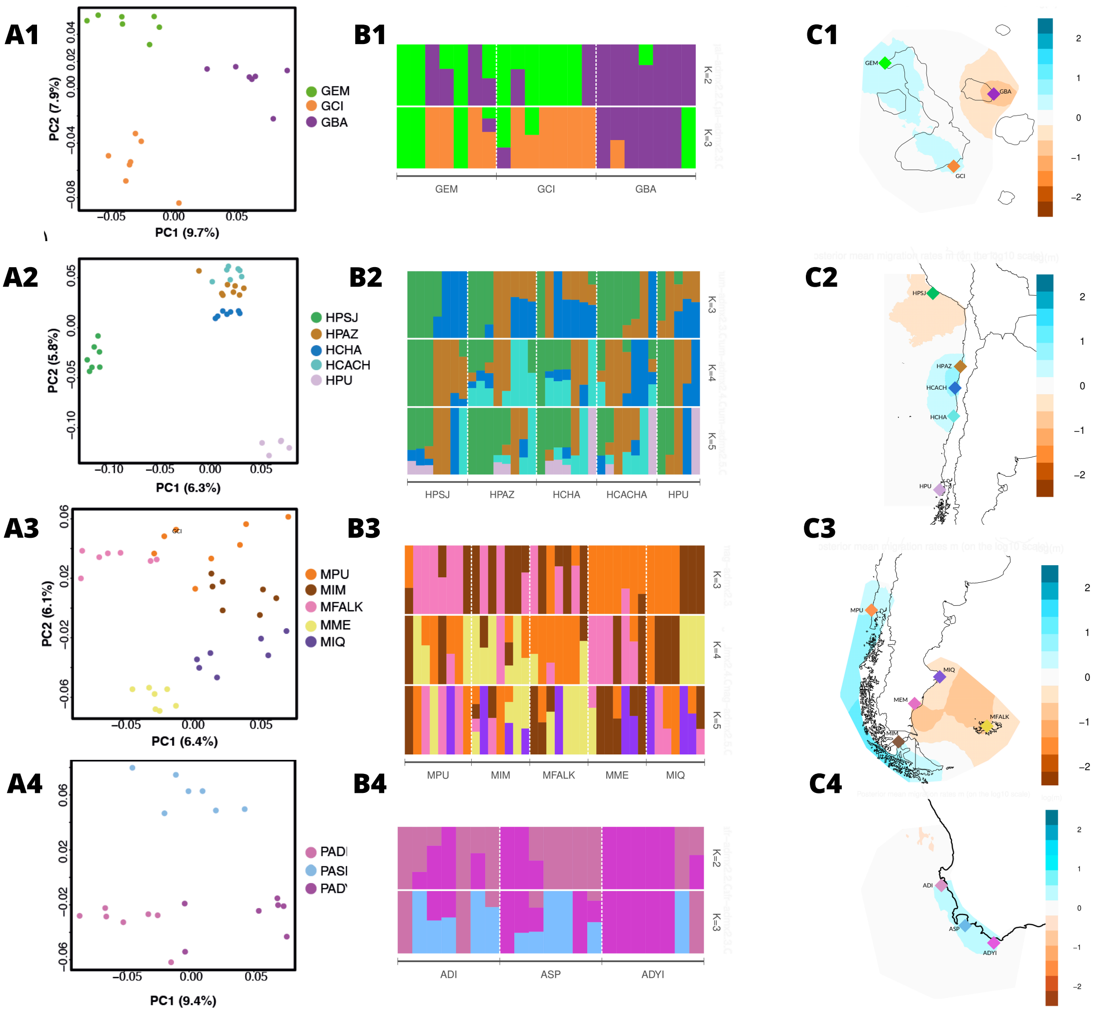

Supplementary Figure 9. **Population structure of the Galápagos, Humboldt, Magellanic, and African Penguins**. **(A)** Principal component analysis of 5000 informative SNPS within each of the 4 banded penguin species. **(B)** Intraspecific ADMIXTURE analysis for each species of banded penguin **(C)** Spatial genomic structure using EMMS with gene flow corridors indicated in blue and barriers to gene flow in orange. Breeding colonies of Galápagos penguin: El Muñeco (GEM), Caleta Iguana (GCI), Bartolomé (GBA); Humboldt penguin: Punta San JUAN (HPSJ), Pan de Azucar (HPAZ), Isla Chañaral (HCHA), Isla Cachagua (HCACH), and Puñihuil (HPU); Magellanic penguin: Puñihuil (MPU), Isla Magdalena (MIM), Malvinas/Falkland Island (MFALK), Monte Entrance (MME), and Isla Quiroga (MIQ); African penguin: Dassen Island (ADI), Stony Point (ASP), Dyer Island (ADYI).

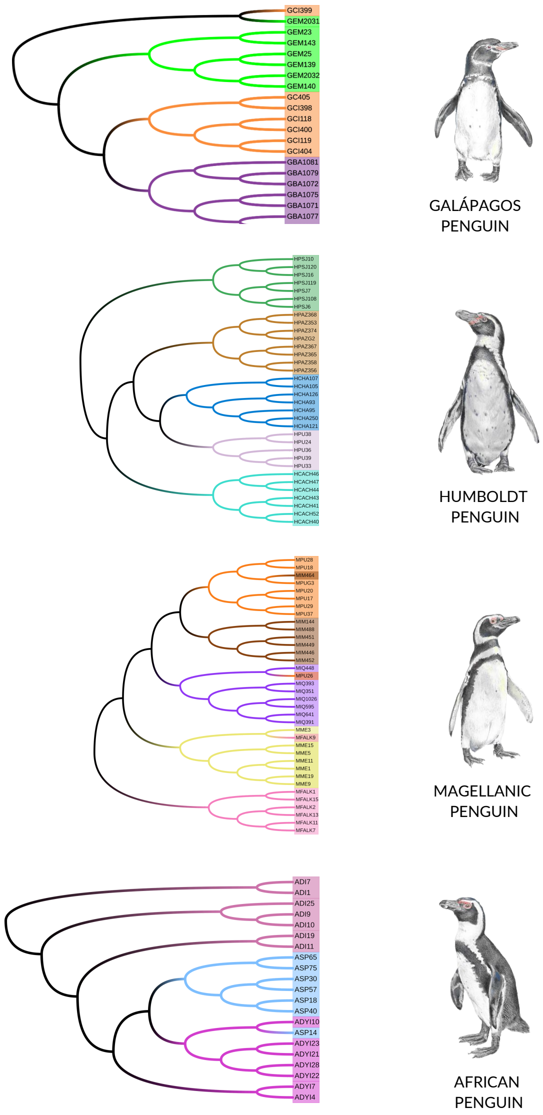

Supplementary Figure 10. Unrooted topology for all individuals from each species. Topology using informative SNPs indicating the genealogical relationships of individuals by breeding colonies (SNPs dataset for each are indicated at Table S2). The acronyms for each reproductive colony are detailed in Table S1.

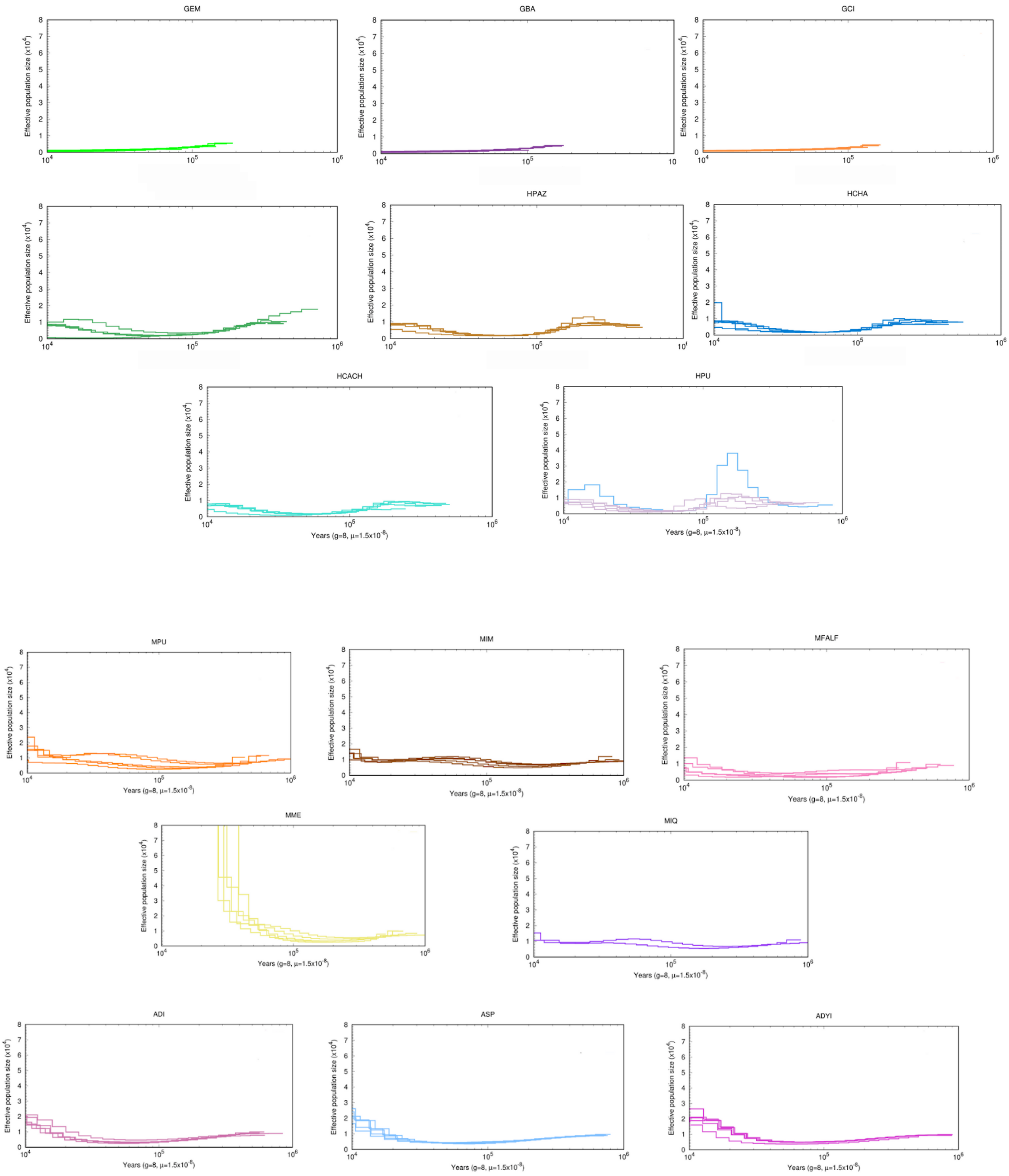

Supplementary Figure 11. **Pairwise sequentially Markovian coalescent (PSMC) estimates of the effective population size (*Ne*) in the 16 banded penguin populations sampled.** Lines represent results obtained from each individual belonging to the same breeding colony. The acronyms for each reproductive colony are detailed in Table S1.

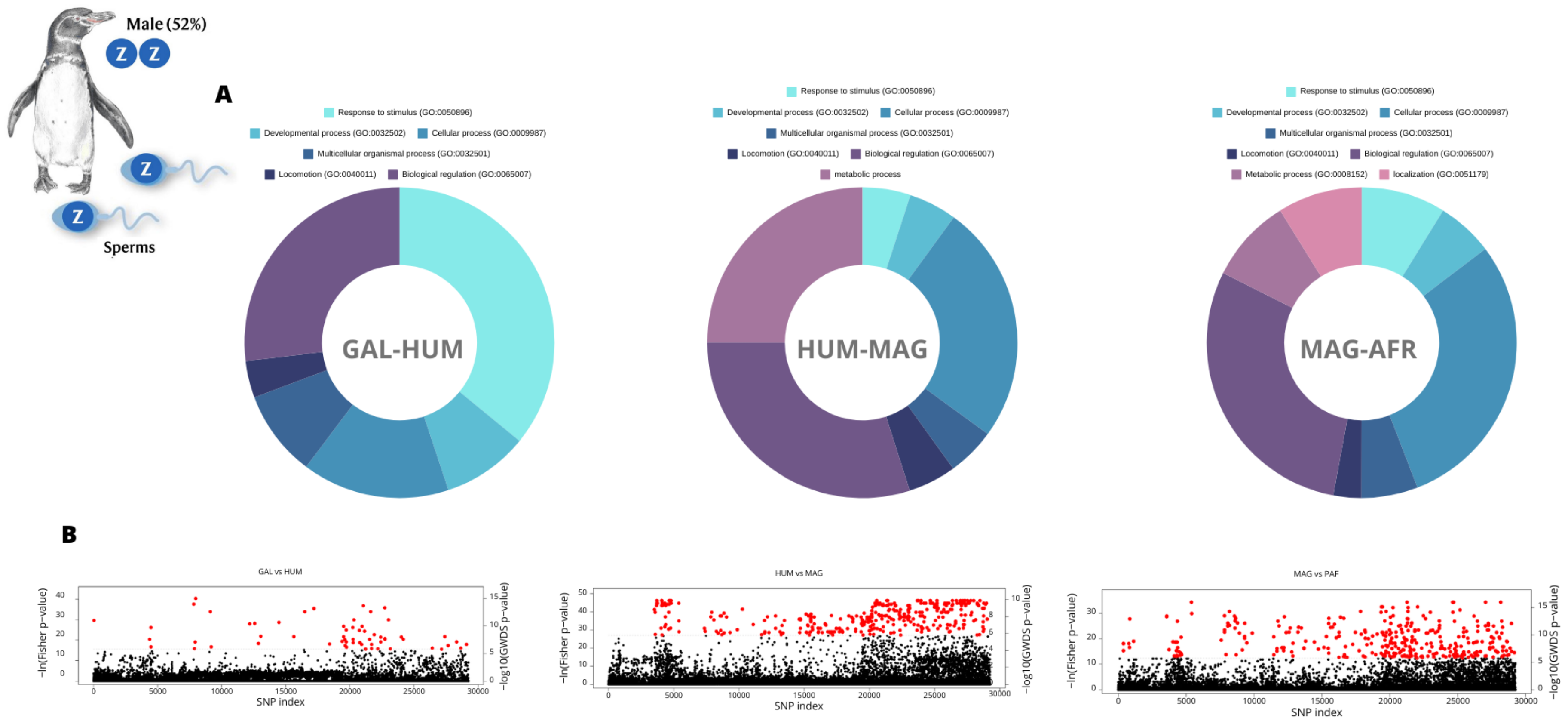

Supplementary Figure 12. ZZ sex-linked scaffolds compared between banded penguin species. (A) Primary Gene Ontology and enrichment analysis. (B) Manhattan plots illustrate the SNPs and their degree of differentiation.

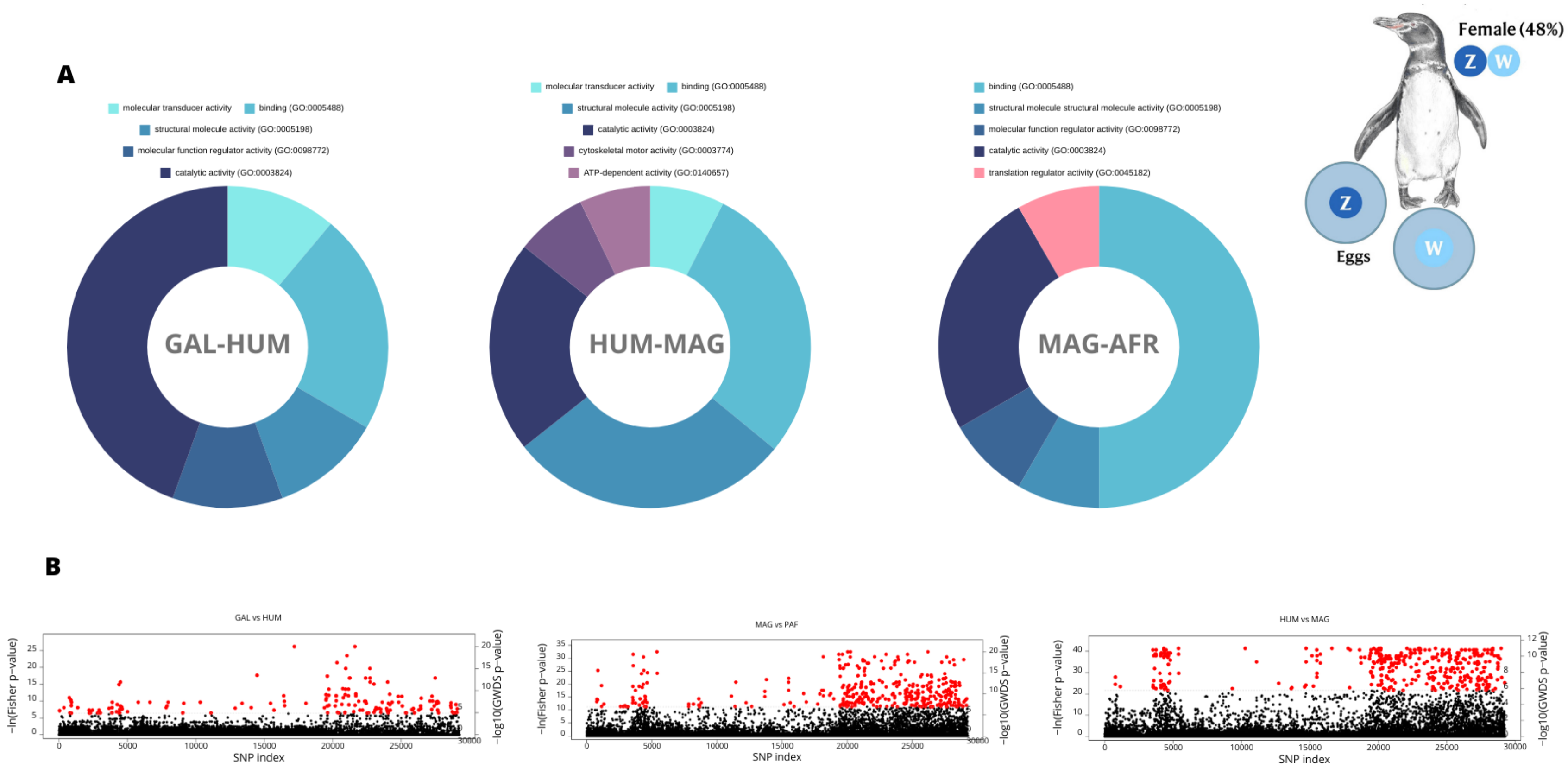

Supplementary Figure 13. ZW sex-linked scaffolds divergent selection between pairwise Spheniscus species. (A) The primary Gene Ontology and enrichment analysis of selection analysis between pairwise species (B) The Manhattan plots illustrate the SNPs between pairwise species and their degree of differentiation.

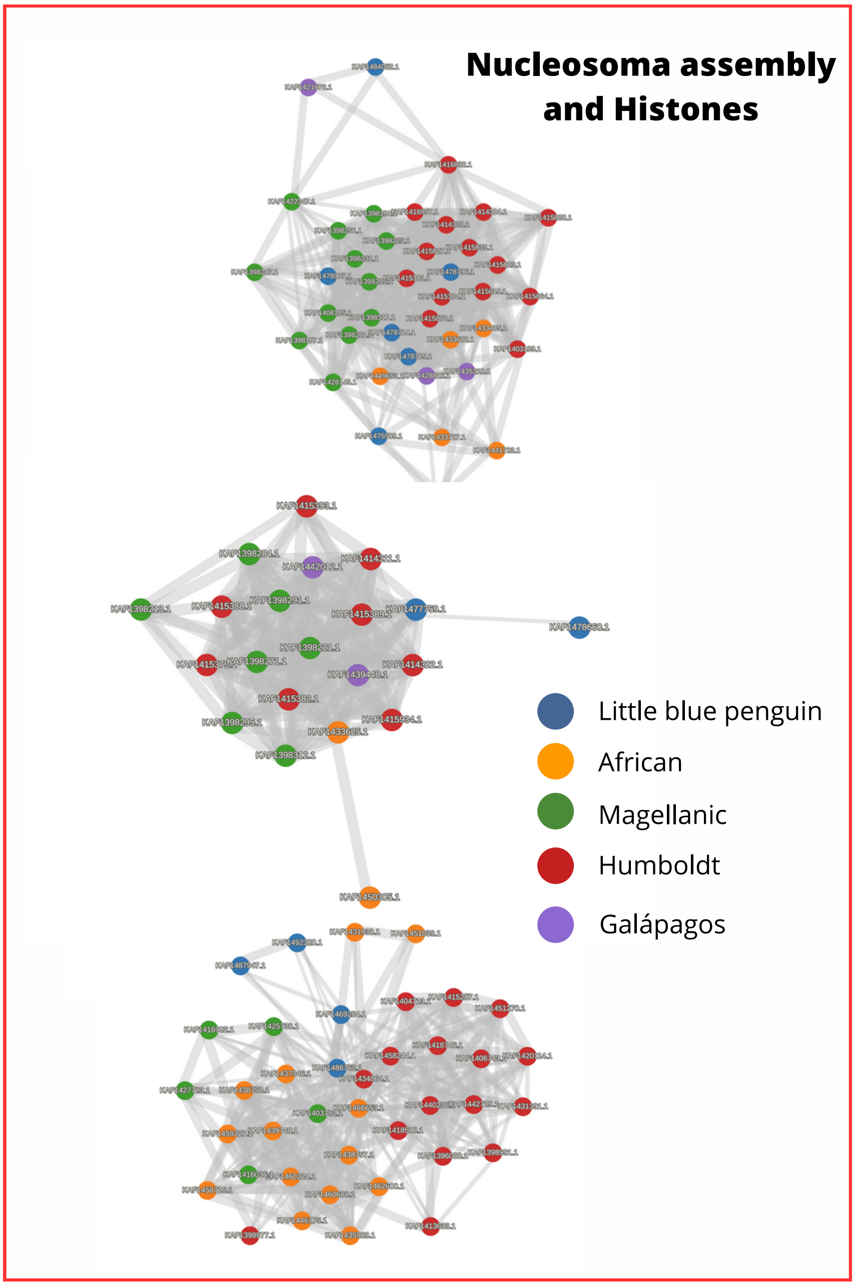

Supplementary Figure 14. Diversification of gene families in banded penguin species compared to the Little penguin, *Eudyptula minor*. Three different networks associated with the expansion of nucleosome assembly gene families among species.

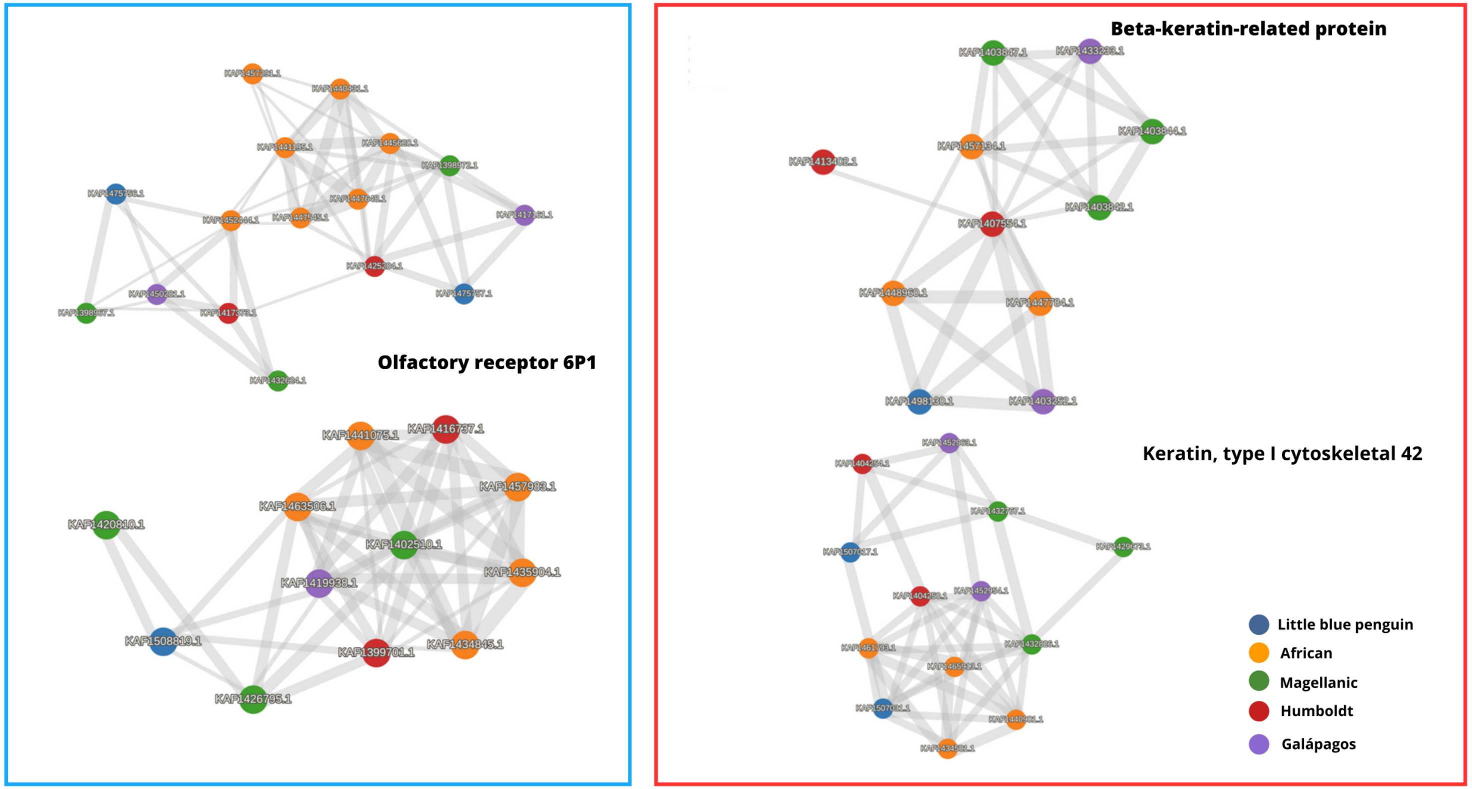

Supplementary Figure 15. Gain of gene families between Magellanic and African penguins. Left: diversification of olfactory receptor (ORs) gene families 6F1 and 6P1. Right: expansion of feather keratin gene families Beta-keratin-related protein and Keratin type I.

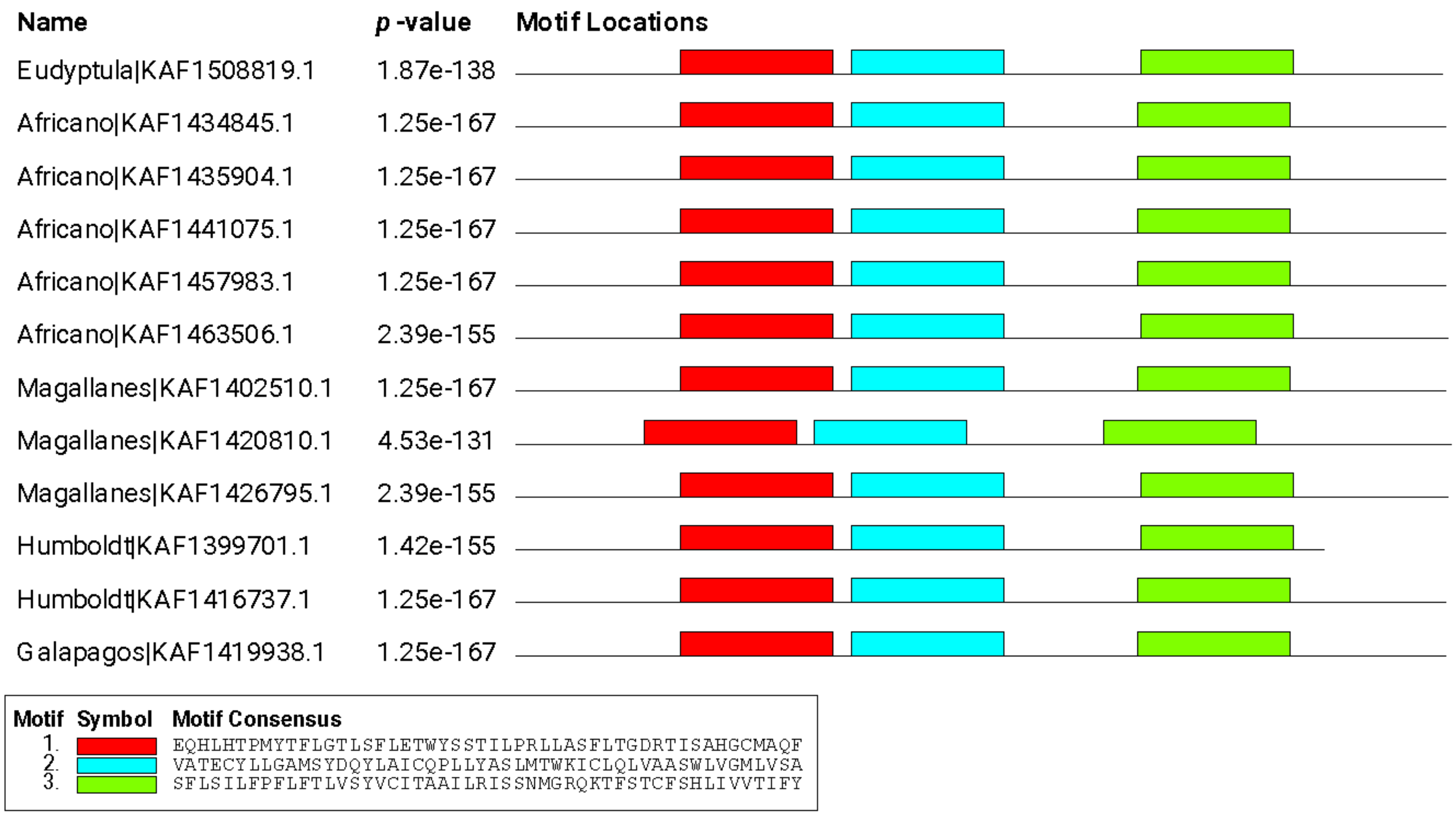

Supplementary Figure 16. Protein motif differences in olfactory receptor families between little penguin and banded penguin. Comparison shows how conservative are the primary amino acid sequences.

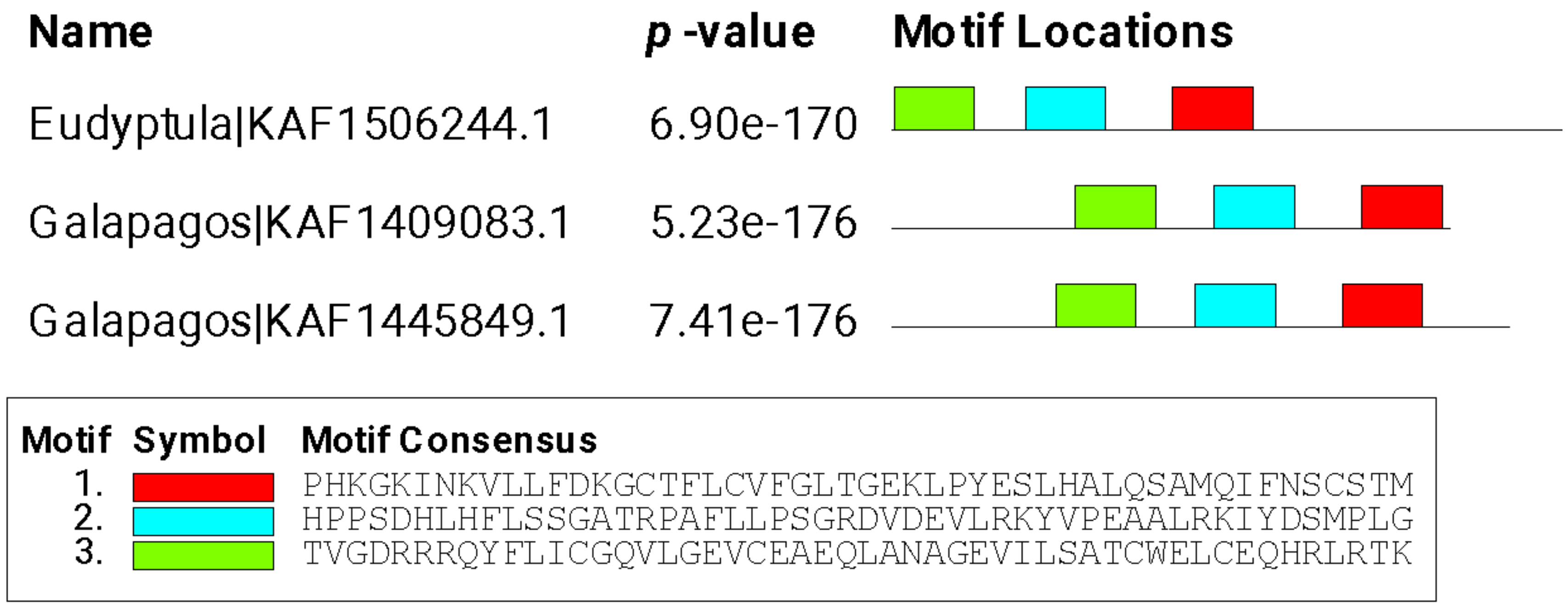

Supplementary Figure 17. Protein motif differences in Spermatogenesis gene family between little penguin and Galápagos penguin. Comparison shows the non-synonymous substitution and the possible implication in the protein secondary structure conformation.

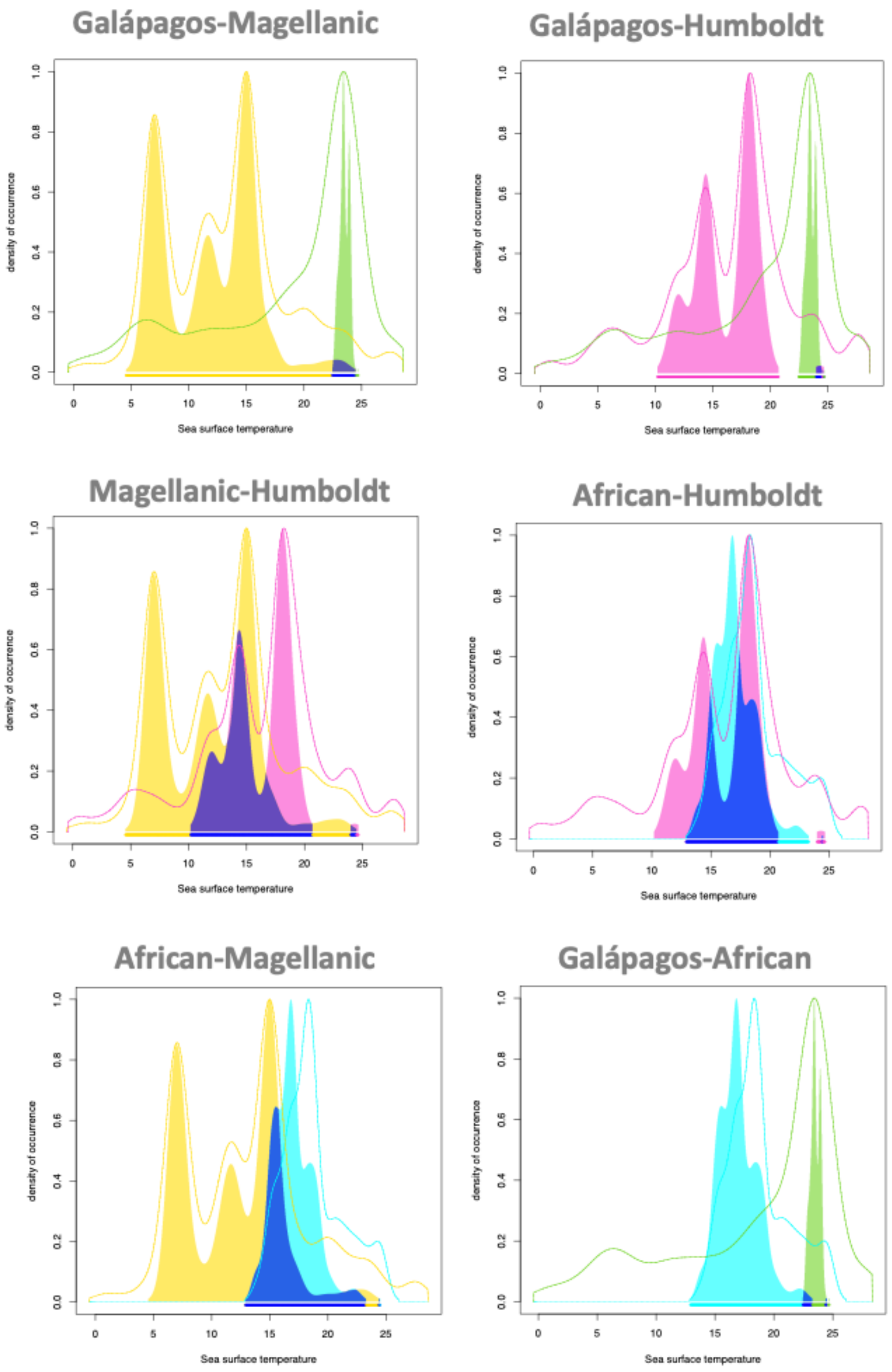

Supplementary Figure 18. Overlap in thermal niche between banded penguin species. Overlap between species: Galápagos (green) and Humboldt penguins (pink); Humboldt (pink) and Magellanic penguins (yellow); Magellanic (yellow) and African penguins (light blue); the

degree if niche overlap is indicated in purple and dark blue. See univariate D overlap values and dynamic niche overlap values in Table S21.

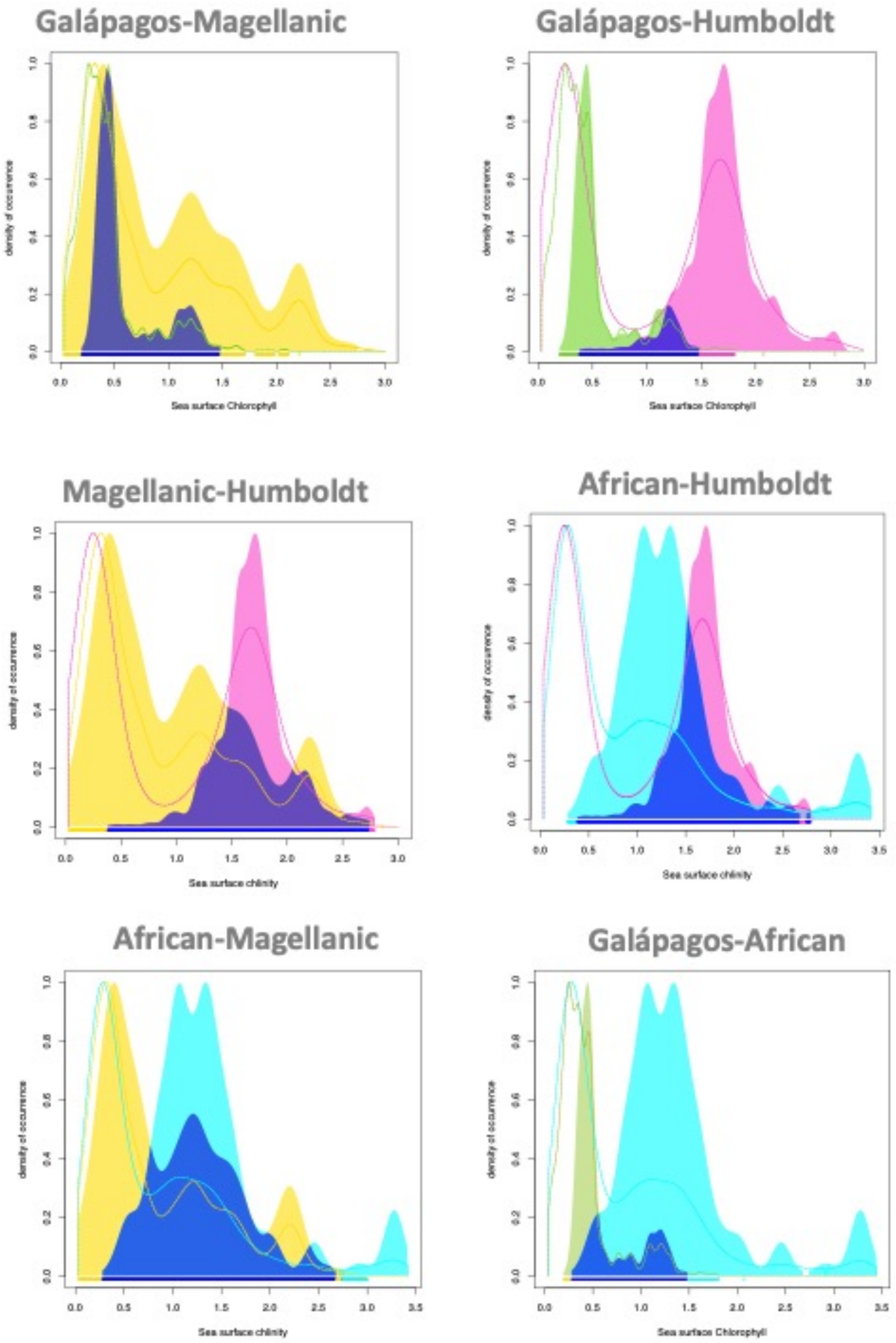

Supplementary Figure 19. Overlap in chlorophyll niche between banded penguin species. Overlap between species: Galápagos (green) and Humboldt penguins (pink); Humboldt (pink)

and Magellanic penguins (yellow); Magellanic (yellow) and African penguins (light blue); the degree if niche overlap is indicated in purple and dark blue. See univariate D overlap values and dynamic niche overlap values in Table S21.

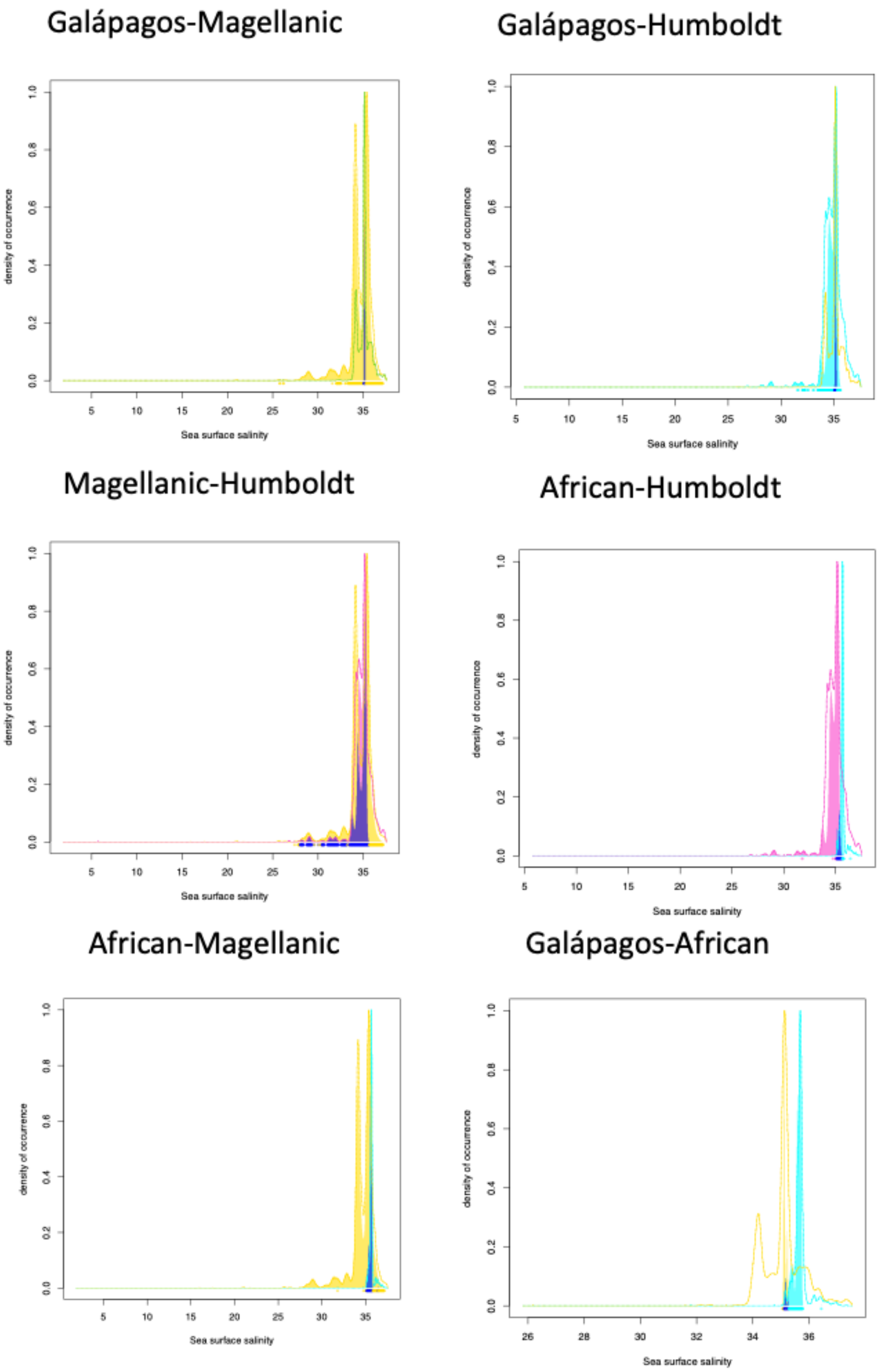

Supplementary Figure 20. Salinity Niche overlaps between pairwise banded penguin species. Overlap between pairwise species: Galápagos (green) and Humboldt penguins (pink); Humboldt (pink) and Magellanic penguins (yellow); Magellanic (yellow) and African penguins

(light blue); the degree if niche overlap is indicated in purple and dark blue. See univariate D overlap values and dynamic niche overlap values in Table S23.

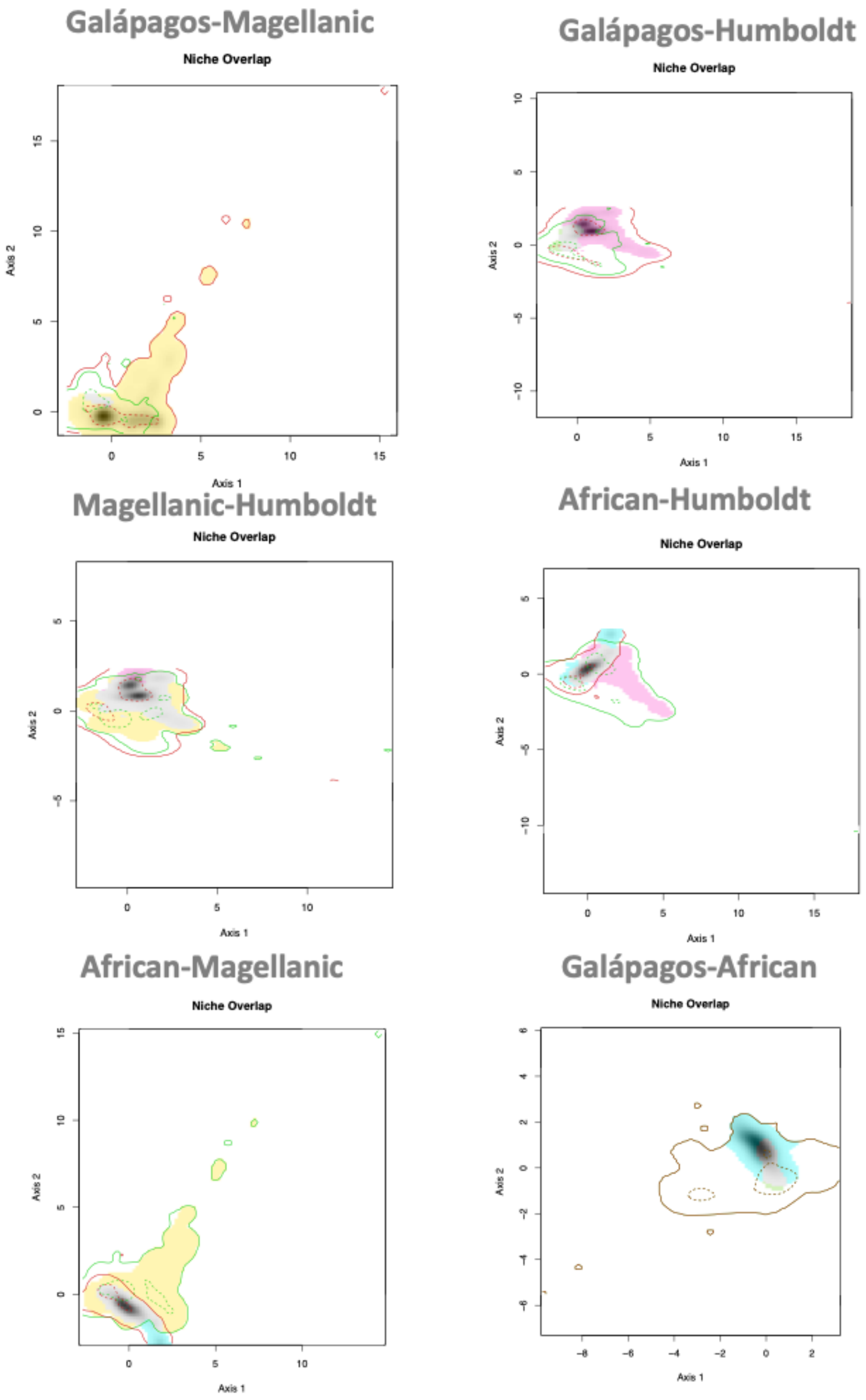

Supplementary Figure 21. Overall Niche overlaps between banded penguin species. See niche D overlap values in Table S24.

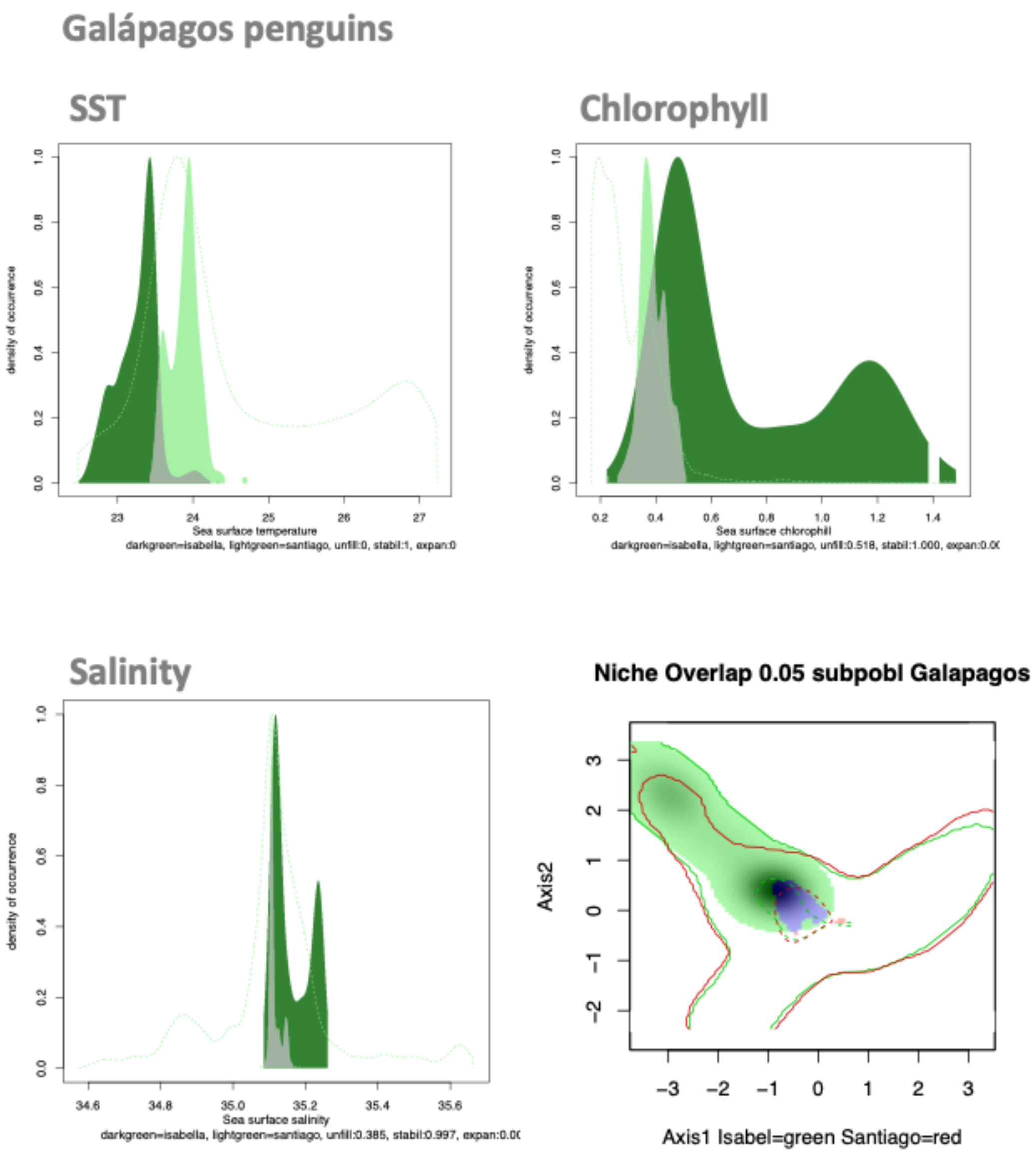

Supplementary Figure 22. Ecological Niche overlaps between EEMS genetic groups for Galápagos penguins. Niche overlap for the EEMS genetic cluster in different tones of green for Galápagos penguin. See all D overlap values and dynamic niche overlap values respectively in Table S25 and S26.

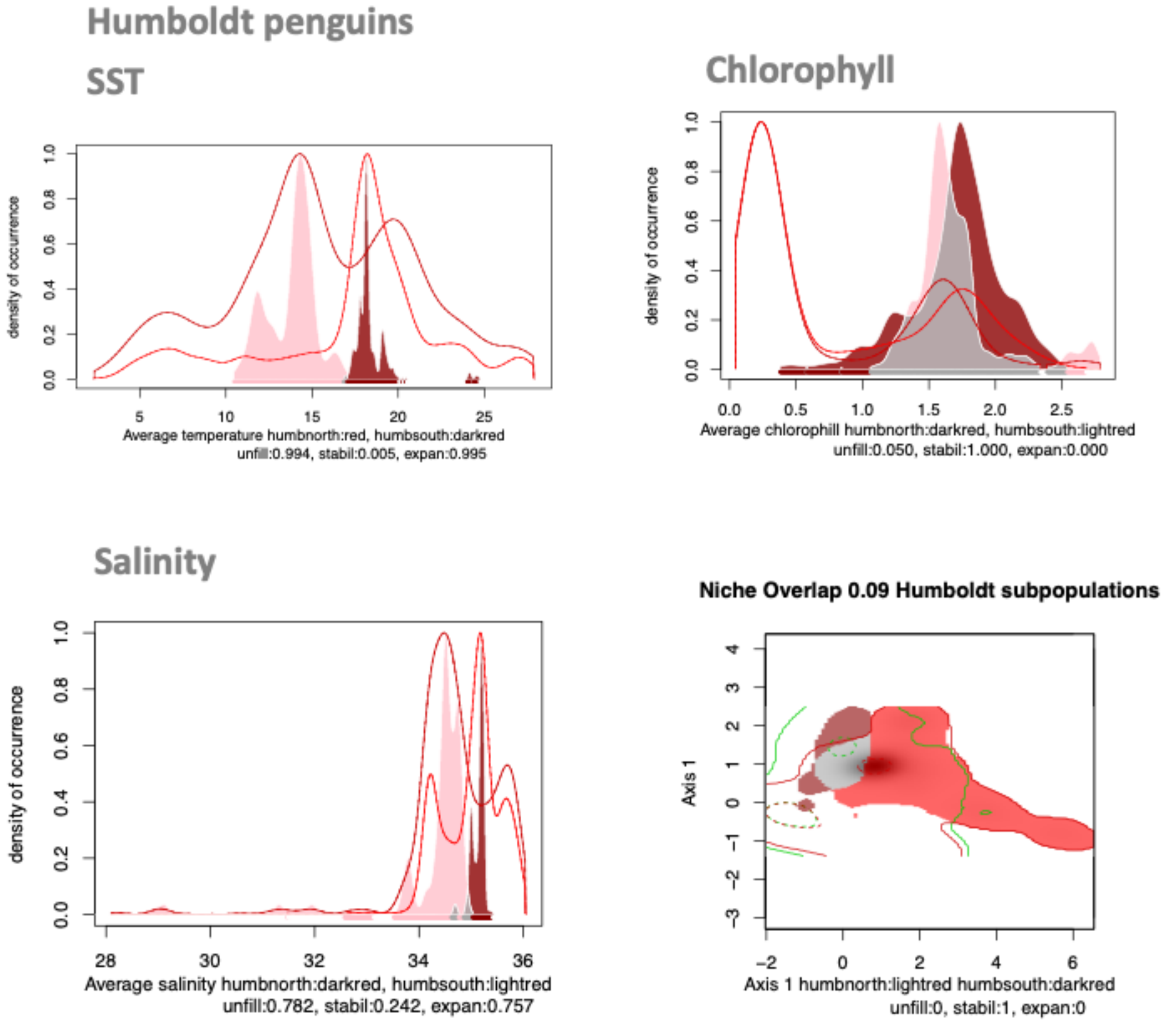

Supplementary Figure 23. Ecological Niche overlaps between EEMS genetic groups for Humboldt penguins. Niche overlap for the EEMS genetic cluster in different tones of pink/red for Humboldt penguin. See all D overlap values and dynamic niche overlap values respectively in Table S25 and S26.

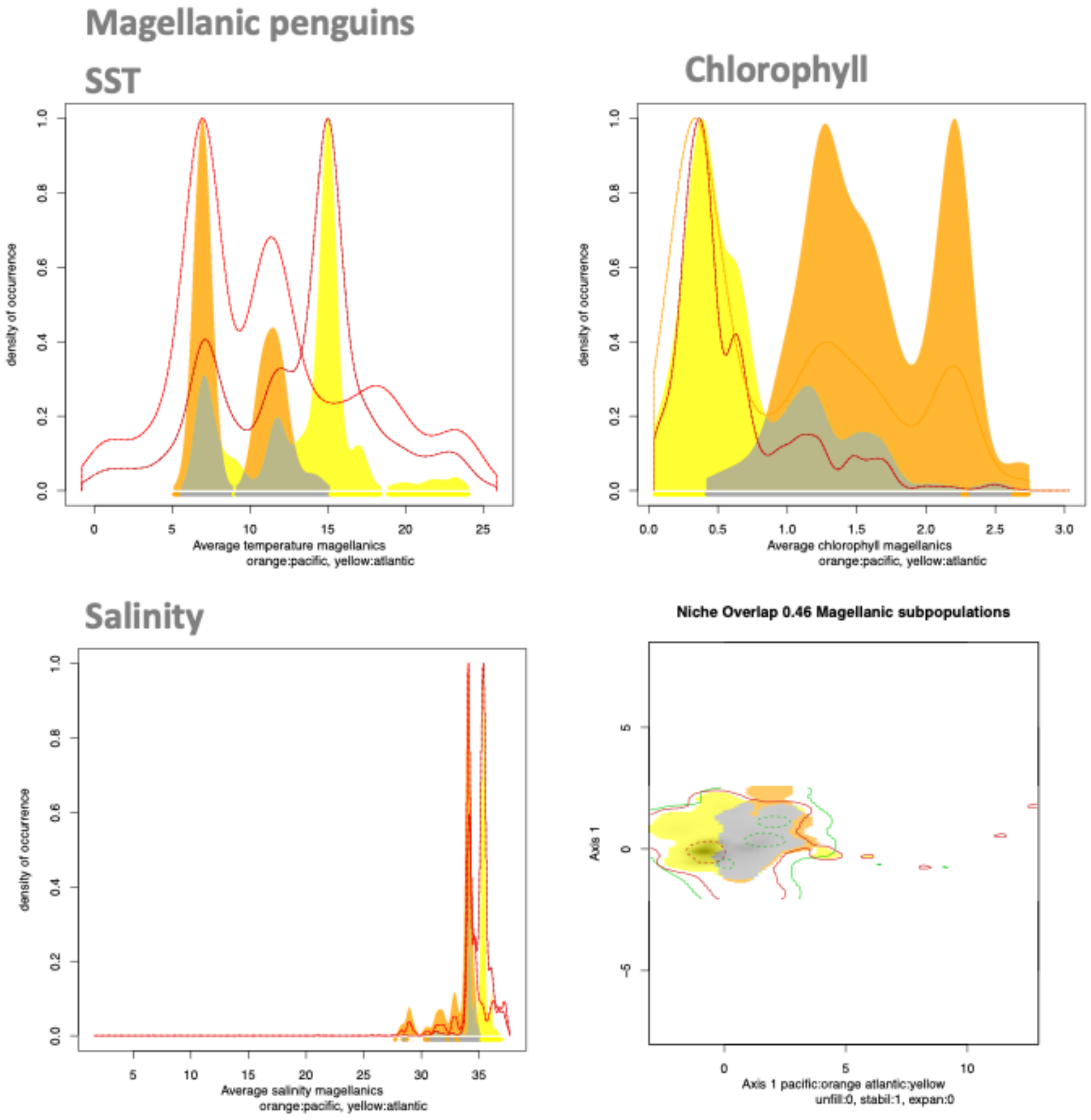

Supplementary Figure 24. Ecological Niche overlaps between EEMS genetic groups for Magellanic penguins. Niche overlap for the EEMS genetic cluster in different tones of yellow for Magellanic penguin. See all D overlap values and dynamic niche overlap values respectively in Table S25 and S26.

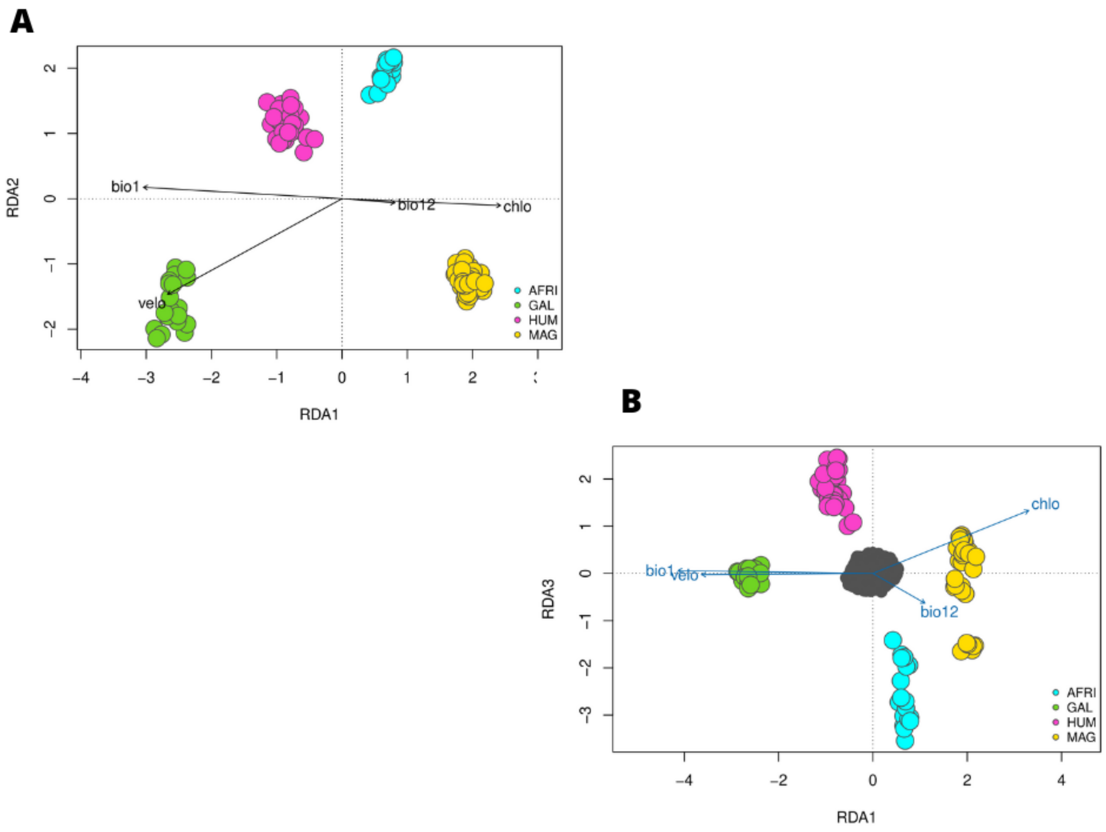

Supplementary Figure 25. Genotype Environment Analysis of the four *Spheniscus* species. The three significant RDA axes (p-value < 0.05) illustrate the relationships among genomic markers, individual characteristics, and environmental influences among penguin species. A) RDA1 vs RDA2 and B) RDA1 vs RDA3. Colored circles represent samples from the different populations and arrows signify environmental variables.

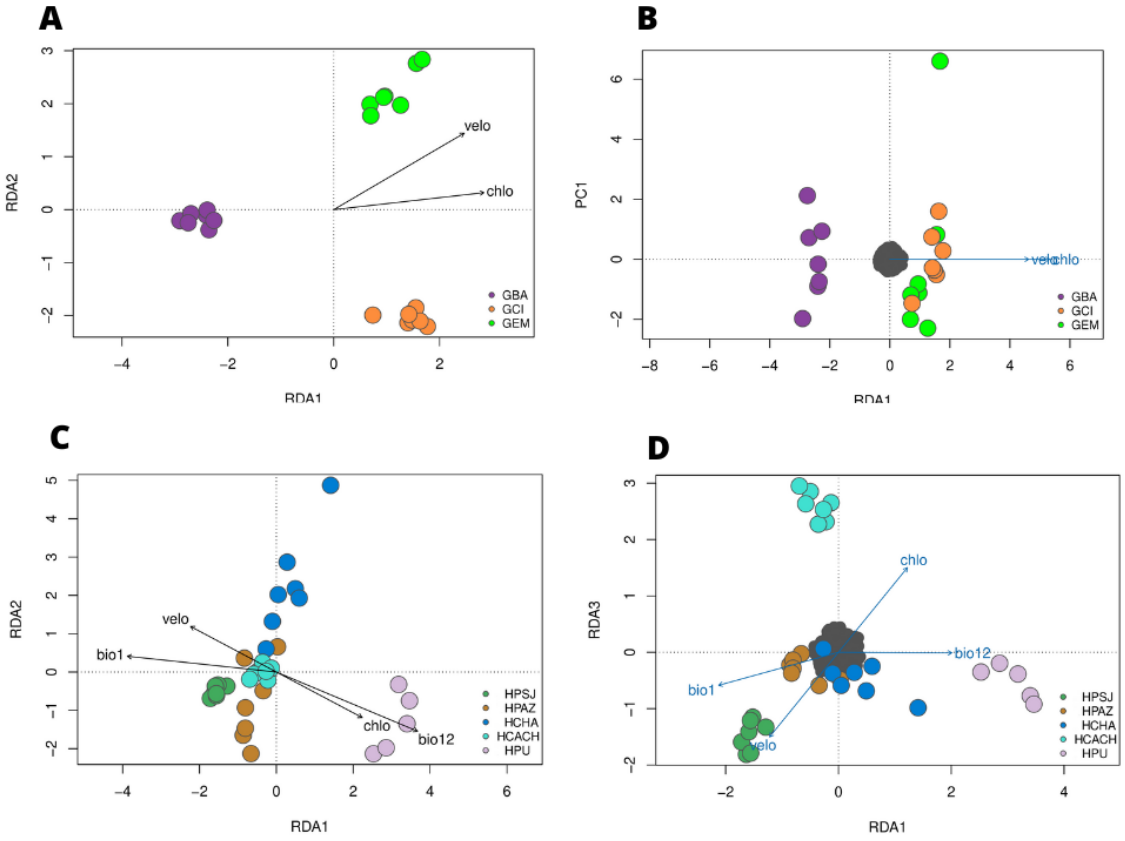
A
RDA1
RDA2
velo
chlo
GBA
GCI
GEM
B
RDA1
PC1
velochlo
GBA
GCI
GEM
C
RDA1
RDA2
velo
bio1
chlo
bio12
HPSJ
HPAZ
HCHA
HCACH
HPU
D
RDA1
RDA3
chlo
bio12
bio1
velo
HPSJ
HPAZ
HCHA
HCACH
HPU

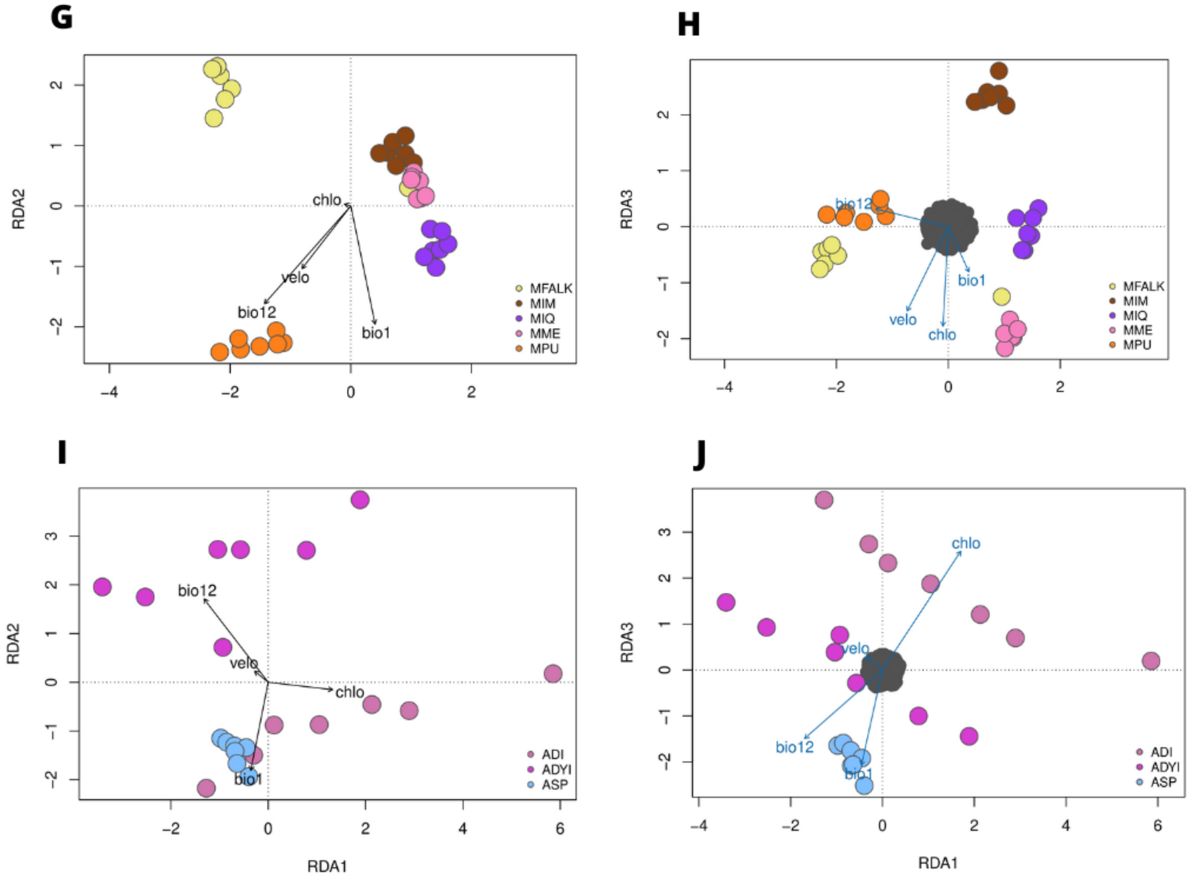

Supplementary Figure 26. Genotype Environment Analysis of the Galápagos and Humboldt penguin populations. Significant RDA axes (p-value < 0.05) illustrate the relationship among genomic markers, individual traits, and environmental factors across penguin species A-B) RDA1 vs RDA2 and RDA1 vs RDA3 of Galápagos penguins, B-C) RDA1 vs RDA2 and RDA1 vs RDA3 of Humboldt penguins, E-F) RDA1 vs RDA2 and RDA1 vs RDA3 of Magellanic penguins and E-F) RDA1 vs RDA2 and RDA1 vs RDA3 of African penguins.

TABLES

Supplementary Table 1. Average genomic coverage per individual and species. Samples in bold were obtained from Vianna et al. (2020).

SAMPLE	MEAN COVERAGE	SPECIES
10ADI	5.1	AFR
10ADYI	9.79	AFR
11ADI	5.97	AFR
14ASP	7.48	AFR
18ASP	6.68	AFR
19ADI	8.10	AFR
1ADI	6.01	AFR
21ADYI	10.72	AFR
22ADYI	6.29	AFR
23ADYI	8.83	AFR
25ADI	5.10	AFR
28ADYI	8.50	AFR
30ASP	6.85	AFR
40ASP	7.17	AFR
4ADYI	6.18	AFR
57ASP	6.36	AFR
65ASP	5.76	AFR
75ASP	3.35	AFR
7ADI	5.08	AFR
7ADYI	3.56	AFR
9ADI	1.84	AFR
**CASCS1**	**30.42**	**AFR**
**G1**	**36.93**	**GAL**
GAPE_1071	9.07	GAL
GAPE_1072	8.63	GAL
GAPE_1074	8.14	GAL
GAPE_1075	5.06	GAL
GAPE_1077	5.08	GAL
GAPE_1079	7.42	GAL
GAPE_1081	7.16	GAL
GAPE_398	5.00	GAL
GAPE_399	7.62	GAL
GAPE_400	6.16	GAL
GAPE_404	7.33	GAL
GAPE_405	4.89	GAL

PR_118	6.92	GAL
PR_119	10.15	GAL
PR_139	6.13	GAL
PR_140	3.89	GAL
PR_143	12.13	GAL
PR_2031	8.50	GAL
PR_2032	4.03	GAL
PR_23	7.85	GAL
PR_25	7.30	GAL
CH105	6.96	HUM
CH107	6.05	HUM
CH121	12.60	HUM
CH126	6.51	HUM
CH144	3.59	HUM
CH24	8.32	HUM
CH250	3.31	HUM
CH26	4.29	HUM
CH29	4.88	HUM
CH33	7.46	HUM
CH353	7.66	HUM
CH356	8.11	HUM
CH358	7.72	HUM
CH36	5.27	HUM
CH365	6.35	HUM
CH367	7.70	HUM
CH368	4.92	HUM
CH374	12.54	HUM
CH38	5.95	HUM
CH39	7.53	HUM
CH40	4.78	HUM
CH41	7.84	HUM
CH43	12.96	HUM
CH44	6.66	HUM
CH449	8.36	HUM
CH451	5.93	HUM
CH452	8.67	HUM
CH46	6.87	HUM
CH464	1.74	HUM
CH47	6.44	HUM
CH488	6.62	HUM
CH52	8.05	HUM

CH93	7.72	HUM
CH95	8.38	HUM
**G2**	**31.23**	**HUM**
PE06	8.43	HUM
PE07	7.67	HUM
PE10	3.26	HUM
PE108	11.28	HUM
PE119	7.02	HUM
PE120	4.67	HUM
PE16	6.05	HUM
CH17	4.50	MAG
CH18	6.52	MAG
CH20	2.75	MAG
CH28	7.23	MAG
CH37	5.23	MAG
CH446	7.61	MAG
**G3**	**36.44**	**MAG**
IQ1026	8.00	MAG
IQ351	6.85	MAG
IQ391	6.54	MAG
IQ393	5.20	MAG
IQ448	5.65	MAG
IQ595	6.44	MAG
IQ641	8.12	MAG
ME1	6.16	MAG
ME11	5.05	MAG
ME19	7.50	MAG
ME15	4.77	MAG
ME3	4.06	MAG
ME5	4.93	MAG
ME9	5.97	MAG
MF1	5.01	MAG
MF11	3.50	MAG
MF13	3.48	MAG
MF15	3.09	MAG
MF2	3.32	MAG
MF7	4.85	MAG
MF9	5.08	MAG

Supplementary Table 2. Details of the sampling. The number of samples by species and by locality, geographic coordinates, and the acronym used for each breeding colony in this study.

Site number	Sample species	Locality	Acronym	Latitude	Longitude	Samples
1	*S.mendiculus*	El Muñeco, Isabela	GEM	0°02´S	91°56´W	7
2	*S.mendiculus*	CE, Isabela	GCE	0°62´S	91°09´W	1
3	*S.mendiculus*	Caleta Iguana, Isabela	GCI	0°96´S	90°96´W	7
4	*S.mendiculus*	Bartolomé, Santiago	GBA	0°28´S	90°55´W	7
5	*S.humboldti*	Punta San Juan	HPSJ	15°36´S	75°19´W	7
6	*S.humboldti*	Islote Pan de Azúcar	HPAZ	26°17´S	70°65´W	8
7	*S.humboldti*	Islote Chañaral	HCHA	29°16´S	71°35´W	7
8	*S.humboldti*	Islote Cachagua	HCACHA	32°59´S	71°45´W	7
9	*S.humboldti and S. magellanicus*	Islote Puñihuil	HPU / MPU	41°92´S	74°04´W	5 & 8
10	*S. magellanicus*	Isla Magdalena	MIM	52°92´S	70°57´W	7
11	*S. magellanicus*	Islas Falklands/Malvinas	MFALK	51°69´S	59°53´W	7
12	*S. magellanicus*	Monte Entrance	MEM	50°08´S	68°22´W	7
13	*S. magellanicus*	Isla Quiroga	MIQ	47°64´S	65°94´W	7
14	*S. demersus*	Isla Dassen	ADI	33°42´S	18°06´E	7
15	*S. demersus*	Stony Point	ASP	34°68´S	19°45´E	7
16	*S. demersus*	Isla Dyer	ADYI	34°37´S	18°89´E	7

Supplementary Table 3. SNPs datasets resulting from different genome filters used for each specific data analyses.

Data Set	Number of Individuals	Number Species and/or population	Analyses	Genome Coverage	Number of SNPS before filters	Number of SNPS after filters	Filters
Data Set 1A	110	4 species 16 population	Interspecific and Intraspecific Genome wide Diversity: heterozygosity,Tajimas' D, nucleotide diversity, private alleles.	3-7X	60076142	202572	Phred scrore≥ 30, read depth ≥ 3, mean read depth 5, a minimum allele count of 2, no missing data.
Data Set 1B	110	4 species 16 population	Interspecific: PCA, Phylogeny, Admixture, Introgression	3-7X		162428	Phred scrore≥ 30, read depth ≥ 3, mean read depth 5, a minimum allele count of 2, no missing data, LD with r>0.5 were removed from sliding windows of 50 SNPS and with 10 SNPS of overlap,removed CDS.
Data Set 2A	110	4 species 16 population	Intraspecific: Admixture, EEMS	3-7X		GAL:86433<br>HUM:109790<br>MAG:144441	Phred scrore≥ 30, read depth ≥ 3, mean read depth 5, a minimum allele count of

					AFR: 90584	2,MAF 0,05 , LD with r>0.5 were removed from sliding windows of 50 SNPS and with 10 SNPS of overlap,remove CDS,no missing data
Data Set 2B	110	4 species 16 population	Intraspecific: PCA Removing gender clustering noise	3-7X	GAL:6489 HUM:17553 MAG:42660 AFR:12673	Phred scrore≥ 30, read depth ≥ 3, mean read depth 5, a minimum allele count of 2,LD with r>0.5 were removed from sliding windows of 50 SNPS and with 10 SNPS of overlap,remove CDS,no missing data and with highest standard desviation MAF
Data Set 3A	4	4 species, 4 population	Demographic inference PSMC	27-30 X		Phred scrore≥ 30, -d 15, -D 45 ,removed CDS.
Data Set 3B	16	4 species, 16 population	Demographic inference PSMC	3-7X		Phred scrore≥ 30, -d 3 -D 14 ,removed CDS.
Data Set 4	114	4 species, 16 population	Demographic inference Stairwayplot	3-7X	162428	Phred scrore≥ 30, read depth ≥ 3, mean read depth 5, a minimum allele count of 2, no missing data, LD with r>0.5 were removed from sliding windows of 50 SNPS and with 10 SNPS of

	overlap,removed CDS.

Supplementary Table 4. Interspecific diversity metrics. Diversity metrics for different banded penguin species. Indexes include nucleotide diversity (π), segregate sites (S) and Tajima's D statistic, and other parameters indicating demographic events and selection pressures.

pop	GAL	HUM	MAG	PAF
mean_nsites	593797.6	594769.3	576528.2	590225.1
mean_π	92994.55	99852.64	114072.03	107210.97
mean_maf	0.27	0.22	0.21	0.26
expected_π	115432.8	123411.6	157894	142229.4
S	292828	359591	475871	369619
harmonic_number	4.28	4.74	4.73	4.23
Watterson	68417.76	75863.08	100606.98	87380.38
TajimaD	24576.79	23989.56	13465.05	19830.59
sd_π	15374.2789	12707.8636	12862.2627	15922.9392
π_scaled	0.1566	0.1679	0.1979	0.1816
π_scaled_sd	0.0259	0.0214	0.0223	27
Watterson_scaled	0.1152	0.1276	0.1745	148
TajimaD_scaled	0.0414	0.0403	0.0234	0.0336
X2	8828.39	7586.02	1802.14	4500.46
pvalue	0	0	0	0
sign	**	**	**	**
rare_alleles	lack_of_rare	lack_of_rare	lack_of_rare	lack_of_rare
demography	contraction	contraction	contraction	contraction

Supplementary Table 5. Introgression signatures from D-suite (ABBA-BABA test). Positive values of the D-statistic show an excess of ABBA-patterns suggesting historical introgression between Humboldt and Magellanic Penguins.

sp1	sp2	sp3	Dstatistic	Z-SCORE	p-value	f4-ratio
GAL	HUM	MAG	0,00310904	15,4867	0	0,301022

Supplementary Table 6. Matrix of *Fst* differentiation among breeding colonies of banded penguins. *Fst* estimated (above) from Weir to Cockerham (1984) with significant p-values (< 0.05) (below) indicated. Breeding colonies of Galápagos penguin (green): El Muñeco (GEM), Caleta Iguana (GCI), Bartolomé (GBA); Humboldt penguin (purple): Punta San JUAN (HPSJ), Pan de Azucar (HPAZ), Isla Chañaral (HCHA), Isla Cachagua (HCACH), and Puñihuil (HPU); Magellanic penguin (yellow): Puñihuil (MPU), Isla Magdalena (MIM), Malvinas/Falkland Island (MFALK), Monte Entrance (MME), and Isla Quiroga (MIQ); African penguin (African): Dassen Island (ADI), Stony Point (ASP), Dyer Island (ADYI). Different colors represent different species and the different intersection between species.

	0.001	0.004	0.004	0.043	0.042	0.042	0.043	0.097	0.105	0.102	0.101	0.102	0.106	0.114	0.116	GEM
		0.005	0.041	0.044	0.041	0.044	0.045	0.1	0.106	0.105	0.101	0.103	0.108	0.114	0.118	GCI
			0.041	0.042	0.04	0.043	0.043	0.1	0.106	0.106	0.102	0.103	0.111	0.115	0.018	GBA
				0.001		-0.001	0.002	0.076	0.083	0.081	0.081	0.082	0.092	0.098	0.1	HPSJ
					0.001	0.001	0.001	0.078	0.085	0.087	0.086	0.085	0.098	0.102	0.104	HPAZ
						0.001	0.003	0.076	0.083	0.084	0.082	0.083	0.094	0.099	0.101	HCHA
								0.077	0.084	0.084	0.084	0.082	0.092	0.099	0.1	HCACHA
								0.072	0.078	0.079	0.075	0.077	0.091	0.096	0.098	HPU
										0.002	0.004	0.004	0.048	0.051	0.054	MPU
										0.004	0.004	0.003	0.051	0.054	0.055	MIM
											0.005	0.006	0.052	0.057	0.061	MFALK
												0.002	0.051	0.056	0.059	MME
													0.005	0.054	0.055	MIQ
														-0.001	0.002	ADI
															0.002	ASP
																ADYI
GEM	GCI	GBA	HPSJ	HPAZ	HCHA	HCACHA	HPU	MPU	MIM	MFALK	MME	MIQ	ADI	ASP	ADYI	

0	0	0	0	0	0	0	0	0	0	0	0	0	0	0	0	GEM
0	0	0	0	0	0	0	0	0	0	0	0	0	0	0	0	GCI
0	0	0	0	0	0	0	0	0	0	0	0	0	0	0	0	GBA
0	0	0	0	0	0	0	0	0	0	0	0	0	0	0	0	HPSJ

**0**	**0**	**0**	**0**	**0.06**	**0.17**	**0.97**	**0**	**0**	**0**	**0**	**0**	**0**	**0**	**0**	**0**	**HPAZ**
**0**	**0**	**0**	**0**	**0**	**0**	**0.12**	**0**	**0**	**0**	**0**	**0**	**0**	**0**	**0**	**0**	**HCHA**
**0**	**0**	**0**	**0**	**0**	**0**	**0**	**0.36**	**0**		**0**	**0**	**0**	**0**	**0**	**0**	**HCACHA**
**0**	**0**	**0**	**0**	**0**	**0**	**0**	**0**	**0**	**0**	**0**	**0**	**0**	**0**	**0**	**0**	**HPU**
**0**	**0**	**0**	**0**	**0**	**0**	**0**	**0**	**0**	**0.46**	**0**	**0**	**0**	**0**	**0**	**0**	**MPU**
**0**	**0**	**0**	**0**	**0**	**0**	**0**	**0**	**0**	**0**	**0**	**0**	**0**	**0**	**0**	**0**	**MIM**
**0**	**0**	**0**	**0**	**0**	**0**	**0**	**0**	**0**	**0**	**0**	**0**	**0**	**0**	**0**	**0**	**MFALK**
**0**	**0**	**0**	**0**	**0**	**0**	**0**	**0**	**0**	**0**	**0**	**0**	**0**	**0**	**0**	**0**	**MME**
**0**	**0**	**0**	**0**	**0**	**0**	**0**	**0**	**0**	**0**	**0**	**0**	**0**	**0**	**0**	**0**	**MIQ**
**0**	**0**	**0**	**0**	**0**	**0**	**0**	**0**	**0**	**0**	**0**	**0**	**0**	**0**	**0.84**	**0**	**ADI**
**0**	**0**	**0**	**0**	**0**	**0**	**0**	**0**	**0**	**0**	**0**	**0**	**0**	**0**	**0**	**0**	**ASP**
**0**	0	0	0	0	0	**0**	**0**	**0**	**0**	**0**	**0**	**0**	**0**	**0**	**0**	**ADYI**
**GEM**	**GCI**	**GBA**	**HPSJ**	**HPAZ**	**HCHA**	**HCACHA**	**HPU**	**MPU**	**MIM**	**MFALK**	**MME**	**MIQ**	**ADI**	**ASP**	**ADYI**	

Supplementary Table 7. Bayesass-estimated contemporaneous gene flow rates between population pairs of the four banded penguin species. Analysis of directional gene flow revealed bidirectional and asymmetrical rates of intraspecific and current gene flow between banded penguins. Values in parenthesis are the confidence interval.

		Migration from				
		El Muñeco (GEM)	Caleta Iguana (GCI)	Bartolomé (GBA)		
	El Muñeco (GEM)	0,760(0,0962)	0,1365(0,0701)	0,1106(0,0804)		
	Caleta Iguana (GCI)	0,861(0,0509)	0,7628(0,0992)	0,0676(0,0472)		
Migration into	Bartolomé (GBA)	0,1539(0,0786)	0,1007(0,0618)	0,8218(0,1015)		
		Migration from				
		Punta San Juan (HPSJ)	Pan de Azucar(HPAZ)	Chañaral(HCHA)	Cachagua(HCACHA)	Puñihuli(HPU)
	Punta San Juan(HPSJ)	0,8888(0,0438)	0,0256(0,0236)	0,0283(0,0266)	0,0281(0,0255)	0,0334(0,0307)
	Pan de Azucar(HPAZ)	0,0275(0,0255)	0,8977(0,0412)	0,0287(0,0265)	0,0274(0,0253)	0,0336(0,0305)
	Chañaral(HCHA)	0,0278(0,0275)	0,0251(0,0228)	0,8876(0,0442)	0,0282(0,0260)	0,0326(0,0294)
	Cachagua(HCACHA)	0,0280(0,0255)	0,0259(0,0240)	0,0272(0,0250)	0,8898(0,0436)	0,0332(0,0300)
Migration into	Puñihuli(HPU)	0,0279(0,0259)	0,0258(0,0242)	0,0281(0,0260)	0,0265(0,0240)	0,8673(0,0500)
		Puñihuli(HPU)	Isla Magdalena(MIM)	FalkLand Island(MFALK)	Monte Entrance(MME)	Isla Quiroga(MIQ)
	Puñihuli(HPU)	0,8954(0,0418)	0,0274(0,0257)	0,0317(0,0288)	0,0283(0,0257)	0,0275(0,0249)
	Isla Magdalena(MIM)	0,0258(0,0243)	0,8891(0,0438)	0,0278(0,0259)	0,0275(0,0248)	0,0278(0,0255)
	Falkland Island(MFALK	0,0258(0,0241)	0,0277(0,0255)	0,8848(0,0452)	0,0281(0,0260)	0,0284(0,0261)
	Monte Entrance(MME)	0,0255(0,0238)	0,0282(0,0261)	0,0280(0,0258)	0,8884(0,0433)	0,0272(0,0245)
Migration into	Isla Quiroga(MIQ)	0,0276(0,0251)	0,0276(0,0254)	0,0278(0,0254)	0,0277(0,0252)	0,8894(0,0429)
		Dassen Island (ADI)	Stony Point (ASP)	Dyer Island (ADYI)		
	Dassen Island	0,7778(0,1028)	0,0797(0,0514)	0,0832(0,0500)		
	Stony Point	0,0545(0,0404)	0,7629(0,0996)	0,0335(0,0304)		
Migration into	Dyer Island	0,1676(0,0914)	0,1574(0,0794)	0,8833(0,0543)		

Supplementary Table 8. Number of SNPs under selection. Number of SNPs recovered by PCAdapt, GWDS and OUTFLANK in each category between banded penguin species.

	CDSs	Genes	mRNA	Pseudogene	Others	Total
**GAL-HUM**	60	169	294	71	51	645
**HUM-MAG**	29	98	115	50	72	364
**MAG-AFR**	47	137	170	86	83	523

Supplementary Table 9. Top ranked genes recovered to be under divergent selection between autosomal and sex-linked scaffolds between pairwise species of banded penguins.

AUTOSOMIC	
GAL-HUM	ABRAXAS2,ABTB2,ACAP3,ACSF2,ACSF3,ADAMTS14,ADAMTS3,ADAMTSL2,ADAMTSL3,ADCK1,ADCY10,ADGRD1,ADK,ADORA1,ADPGK,Adprh,AGPAT3,Akap8,ALK,ALPK1,ALS2CL,AMBRA1,ANK2,ANK3,ANKRD11,Ano4,ANOS1,ANTXR1,ANXA8,AOAH,APLF,ARHGAP20,Arhgap24,Arhgap25,ARHGAP40,Arhgap6,ARID4A,ARSJ,ASB1,ASB11,Asb13,Asb2,ASIC2,ASL,ASTN2,Asx,ATG13,ATL1,Atp10a,ATP6V1H,ATRN,ATXN7L1,AXIN2,B4GALNT3,BARX2,BAZ2B,BCL2L13,Bmp2k,BMPR1B,Boc,BPI,BTBD11,BTN3A2,C4,Cacna1c,CACNA1G,Cacna1h,CadN,CAMKK1,CAMTA1,Capn5,CASP9,CASQ2,CBS,Ccdc30,CCDC42,ccdc85c,CCNI,Cd34,CD8B,CDH13,CDH2,Cdh23,CDH4,CDS2,Celsr1,CEMIP,CEPU1,CFAP61,CFTR,CHCHD3,CHL1,CHN2,CHST11,CLCN6,CLIP2,CLNS1A,Clvs1,CNNM2,Cnrip1,CNTN3,CNTN4,Cntnap2,COL22A1,COLEC12,COLQ,COQ2,CORO1C,Cox10,Cps1,crispld2,CSRNP3,Cstf3,CTNNA2,CTPS1,CTTN1,CUBN,CUL9,Cux1,CYB5R3,CYP2U1,CYT,CYYR1,daam1,DBH,dcakd,DCPS,DDR2,DDX31,DDX42,Dennd2d,DGKZ,DHRSX,DHX37,DISC1,Dlgap2,Dmbt1,DNAH14,DOT1L,DPAGT1,Drc1,DTX2,E2F4,Ebf1,EDC4,Efcab5,EGF,egfl6,EGFL7,EIF4ENIF1,ELF1,Elk3,EMP1,EMP2,ENDOD1,Enkd1,ENOX1,ENOX2,ENTPD8,EPAS1,EPHA10,ERBB4,ergic3,ESPL1,ESRRB,ETS1,EXOC6B,FAM120B,FAM76A,FAT2,FAXDC2,FBXO5,FCHSD2,Fgf18,FHAD1,FHIT,Fmnl2,FOXO1,FOXP4,Frmd4a,FSTL4,GABRA3,GABRR2,GALNT14,Galnt17,galt,Gas6,GBA,GBF1,GCN1,GDPD5,GLI2,Glra4,Gnai3,GPATCH8,GPC3,Gpx1,GRAMD1B,GRIA1,Gria3,GRID2,GRIK3,Grin1,GRIN2B,GRIP1,GRK3,GRM4,GSK

	3B,GTF2E2,GTF2I,GUCA1A,GULO,H2A1,H6PD,HDAC4,HDAC7,HECW1,HHIPL2,HIVEP3,Hlf,HMBOX1,HMGCLL1,HPGDS,HS3ST4,Hsd17b11,HSD17B12,HTR4,Htra1,IGDCC3,IGF2BP3,IQGAP1,Iqsec3,ISM1,ITGA9,ITPR1,KANSL1,Kcnd3,KCNH1,KCNH5,KCNH8,KCNJ3,KCNK2,KCNN3,KCNQ5,KCTD16,Khdrbs1,KIF26B,KIF9,Klhdc8a,Klhl12,KLHL14,Klhl42,KYNU,LAMA4,LAMB3,LDHA,LEF1,Lgr6,LMCD1,lmo1,LNP1,LRGUK,LRRC28,LRRC75A,LRRK1,lrwd1,LSAMP,MACF1,MAFK,Mamu,Map2k4,Map4k3,MAPKAP1,MARCH4,mast3,MB,MDFIC2,MDGA2,MED21,MED27,MEGF11,Mei1,metrnl,MFSD11,Mfsd14a,MGAT5B,MGLL,micu1,MID1,MKRN2,MN1,Mob2,MOGAT2,MORN5,MRC1,MROH2B,MSRA,MTHFD1L,MTHFD2L,MTHFSD,MTMR4,MTOR,MVB12B,MYB,mybbp1a,MYH1B,MYO1D,MYOG,MYSS,n4bp3,Nacc2,NAV1,NAV2,NBEA,NDNF,NDST4,NEK10,NEL,Nemf,NEURL1,Nfrkb,NFS1,NKAIN2,NLGN3,NMT2,NOL4,Nptx2,Nt5dc2,NTNG2,NTRK3,NUAK2,Nxn,NXPE3,OLFM4,Olfr6,OPHN1,OR10A4,ORAI2,Otop2,Oxsr1,P4HB,Pacs1,Pak5,PAPLN,Pappa,PARP1,PARP14,PCDH1,Pcdh15,PCDH18,Pcsk2,PCSK6,PCYT2,PDIA5,Pdk3,PDLIM4,PDLIM5,PDZD8,PEMT,PGM3,PHACTR2,PI4KB,Piezo2,Pigq,PIK3AP1,PIK3C2G,PIK3R4,PINX1,Pitpnm2,pitx3,PKD1L2,PKDCC,PKHD1,PKNOX2,PLEKHD1,PLG,PLXNA2,PLXNA4,PLXNB2,Plxnd1,PNPLA1,PODXL2,Pof1b,POLB,POLE,POLRMT,PPEF1,PPFIA4,PPM1H,PPP1R12B,PPP2CA,PPP6R2,PRDM16,PRICKLE2,PRKAG2,PRKCH,PRPF3,PRPSAP1,Psd,PTCHD1,Ptchd4,PTH1R,PTPDC1,PTPRA,PTPRT,RAB11FIP3,RADIL,Raly,Ranbp10,Rapgef5,RASSF8,Rcc1l,REEP3,RELN,REPS2,REXO2,Rgs9,RHOF,Rhoq,RIF1,RNF14,RNF19B,RNF43,RPA2,RPL14,RPN1,RPS4,Rptor,RSF1,RSPO3,RTN1,Rxra,SARS,SBF2,SCG3,SCTR,SEC31B,SEPT11,SFXN1,SH3GL1,SH3PXD2B,Sh3rf2,SHC4,SIK2,SIK3,SLC18A2,SLC25A21,SLC25A26,Slc29a1,SLC2A13,SLC36A1,SLC4A11,SLC4A3,SLC4A8,Slc5a7,SLC6A1,SLC6A12,SLC7A5,SLC8A3,Slco2b1,SLCO3A1,SLCO4A1,Smtnl2,Sntg2,SNX31,SOX5,Spag4,SRCIN1,SRPK,SSPN,ST8SIA1,Steap3,STIM1,Stk36,STS,STX1A,STX8,STYK1,SUN3,Susd4,Synpr,SYT6,TBC1D16,TBCD,tbrg4,TBX22,TCERG1L,TENM1,tfap2e,TGFBR2,TGS1,TIAM1,TIMM44,TLL2,Tm7sf3,TMC2,TMEFF2,Tmem2,TMLHE,Tmtc2,TOM1,TP53BP2,TP73,TRAF7,TRPC3,Trpc5,TRPC7,TSN,TSNARE1,Tspan14,TSPAN15,TTC28,Ttll10,TUBA1C,Twist2,TXLNB,TYW1,Ube2h,UBE2Q2,Ugt1a1,Ugt2a2,Ugt8,UIMC

	1,ULK1,UQCRC1,USP48,UVS2,Vav2,VEGFD,Vipr1,WARS,WASF2,WDFY3,WDR93,WNK1,Wnt3,WNT3A,wnt8b,WSCD1,WWP2,Xkr6,XPO6,YTHDC1,ZDHHC12,ZDHHC15,Zdhhc4,ZDHHC8,ZDHHC9,ZFAND1,Zfyve19,ZMYND12,ZNF19,ZNF3
HUM-MAG	Abcd4,ABCF3,ABTB2,ACAP3,ACAT2,ACSL6,ACSS2,ADAMTSL2,Adarb2,ADGRL3,AFF1,Agap1,ahcy,AKT3,ALS2CL,APRT,AQP2,ARHGEF26,ARHGEF9,ARMC9,Asb12,ASB18,asrgl1,ATP8A2,ATRN,BEGAIN,BFSP1,BMPER,C2CD2L,Cacna1h,CACNG4,CAMK1G,Capn5,Ccdc30,Ccdc85a,ccdc85c,Cct3,CDH4,CERS6,CFAP20,CFDP1,CHL1,Chmp3,CLASP1,CLEC19A,CNTNAP5,COL22A1,Col27a1,Cpne4,Cps1,CRISP2,CYP46A1,daam1,Dcx,DDX19B,DGKD,DHRS12,DHX37,DLGAP4,DOCK11,DTYMK,DUSP28,EFCAB11,EIF2AK3,EIF2B5,Elp6,EML6,EPB41,EPHB1,Epn2,Eppk1,ERBB4,ERI3,ESRP1,EXOC2,EXT1,FAM76A,FAM83H,FGFRL1,FLT1,FLT3,FOXN3,FOXO1,FOXP4,FSTL4,Galns,GALNT14,GALNT15,GALR1,GATAD2B,GLG1,GLI2,GPATCH8,GPC3,Gria3,GRIN2B,GRM1,HBS1L,HDAC4,HEATR1,HIVEP3,Hsd17b11,IGF2R,Il1rapl2,ILKAP,INTS9,Iqcj,ISM1,KCNAB1,Kcnd3,KCNK13,KCNK2,kcnk9,Kdf1,KDM3A,KIF13B,Klf15,KLF7,KLHL29,KTN1,LAMB3,LAMP3,LIMD1,LMO2,LNX2,LY75,Map2k4,MAP3K12,MAP3K4,MARCH4,Mbnl1,MDFIC2,MDGA2,MDH2,MED12L,MED20,MERTK,Mfn1,MLLT6,MN1,MOGAT2,MPRIP,MROH7,MRPL12,MT,MXRA8,MYO1D,n4bp3,NAALAD2,NAXD,Ndrg1,NDST4,Necab2,NECTIN1,NFKBID,Nipal2,NKAIN1,NOX3,NRBP1,NRP2,NTRK3,NTSR1,OGDH,Pak5,PASK,PDLIM1,PHF11,PHR,Pisd,PKHD1,PLCH1,PLD1,Plec,PLG,PLXNA2,POFUT2,POU2F3,PPM1G,PPP2R3A,PRICKLE2,PRKCA,PROC,PROSER1,Prpf6,PRR5L,PTPN11,PTPN14,PTPRA,Ptprk,RAB11FIP4,RBMS1,RCOR1,Reep1,RFLNA,Rilpl1,RYBP,SATB2,SCAMP4,SCD5,SDK1,SEC31A,SEPT6,SERGEF,SETD2,SFMBT1,SGK1,SH3PXD2B,SIK3,SLC13A3,Slc15a2,SLC22A5,SLC2A12,slc30a8,SLC35A1,Slc5a10,SLC6A14,SLC9A2,Slco2a1,smarcal1,SNAP47,SNRNP40,SNTB1,Snx17,Snx27,SPACA1,SPP2,SPRYD7,Spsb1,Srrm4,SSFA2,ST3GAL3,SUSD2,Sybu,T,Tab1,TCEA2,TECRL,TG,THAP4,TLK2,TMCO4,TMEFF2,TMEM18,Tmem2,Tnik,Tp63,TRAK2,TRAPPC9,Tspear,TTC12,TTC28,Ubash3b,UBE2G2,UBE4A,UBQLN4,Vav2,WISP1,ydjc,ZMYND11
MAG-AFR	A2ML1,ABCA10,ABTB2,Acot5,ada2,ADAM9,ADAMTS10,ADAMTS2,Adarb2,ADCY1,ADCY9,ADGR

	D1,ADGRL3,ADTRP,AFAP1L2,AFDN,AFF3,Agbl4,AHSG,AK4,AKAP12,Alkbh5,ALPK1,ALX4,Amn,AMPD1,ANK3,ANKRD26,Anks3,ANP32E,AR,ARHGAP20,ARNT2,Asb10,Ascc1,ASS1,ASTN2,Asx,Atp10a,B3GNTL1,BARX2,BCAS1,BCOR,BEGAIN,BEND7,Boc,Brf1,BRPF3,BSN,BSPRY,C1S,C2CD3,C8A,CACHD1,CACNA1G,Cacna2d4,CACNB2,CAMTA1,CAPN8,Carhsp1,CASC4,CBS,CCDC25,CCDC9B,CCND3,Cd72,Cdh22,Cdh23,Celsr1,CEMIP,CEPU1,CERS3,CFAP47,CFH,CFTR,CHAF1B,CHGB,CIZ1,CLASP1,CLCC1,CLCN4,CLDN11,CLIP2,Clvs1,CMIP,CNKSR2,Cntn6,CNTRL,COL6A2,COLQ,COMMD9,COPA,CORO1C,CP,Cpne4,CPSF6,Cr1l,cracr2b,CRAT,CRHR1,CSK22,CSMD1,CSMD2,Ctnna1,CTNND2,CTTN1,CUX2,Cyfip1,CYP2J2,CYYR1,daam1,DAGLA,Dedd,DEPTOR,DGKZ,DHX37,DHX38,Dhx40,DLGAP4,Dnaaf5,DNM3,Dock4,DOPEY1,DPAGT1,DPF3,DPM1,DROSHA,DTX2,DUSP13,EDC4,EEPD1,EFHC2,EHBP1,EHF,EIF4E2,ELMO1,Elp6,EML6,EPHA10,EPHB2,Epm2a,Eppk1,EPSTI1,ERBB4,ERC1,ERI3,ERP27,ESRRB,ETS1,EXOC6B,F8,FAF1,FAM13C,Fam19a1,FARP2,FBN1,FBXO16,FBXO45,Fbxw8,FCHSD2,FGFR2,FGFRL1,Fggy,FILIP1L,FIP1L1,Flcn,FN1,FNIP1,FOXO1,FRAS1,Frmd4a,FSHR,FSTL1,FSTL4,GABRA3,Gabrr1,Galns,GALNT14,Galnt17,GAREM1,Gatd1,GDPD5,GJA9,GLA,GLI2,Glp1r,GLP2R,Gmeb2,gna0,Gnal,GPC1,GPC3,GPC5,GPRC5B,Gps1,GRB10,GREB1L,Gria4,Grin1,GRIN2B,GRK3,GRM1,GRM4,GRM7,GUCY2C,H1,HCN4,HDAC4,HDAC8,HEPH,HIPK2,HMGXB3,hnf4a,HORMAD2,HPSE2,Hrh2,HS3ST3B1,HS3ST4,HSPA9,Htr2c,IFNG,IGBP1,IGSF3,Il1rapl2,INSR,IQSEC1,Iqsec3,ISM1,ITGA9,ITGB5,Itih5,ITPKB,JAKMIP2,JARID2,JPH2,Jup,KANSL1,KCNAB1,KCNAB2,Kcnd3,KCNH6,KCNJ6,KCNQ2,KHDRBS2,KIF13B,KIF18B,KIF26B,KIF9,KLF12,Klhl10,KLHL3,KMT2A,kpna1,KYNU,LAMA2,LARGE1,Lcp1,LDAH,LEF1,lhpp,LRP8,LRRC75A,LRRK1,LSAMP,M6PR,MAGI1,MAGI2,MAIP1,MAML3,MANBA,MAP10,Map2k4,MAP2K6,MAP4K5,MARK1,mast3,Mbnl1,MBNL3,MDGA1,MED13L,MEGF9,MET,METTL7A,Mfsd4a,Micu2,MID1,MINPP1,MIPEP,MLF1,MLPH,Mob1b,MOB3C,MPP1,MSX2,MT,MTHFD1L,MVB12B,MYB,MYH1B,MYMK,MYO5A,Myo7a,n4bp3,NBEA,NDST2,NDST4,Necab2,Necap1,Nemf,NFASC,NHSL1,Nipal2,NKAIN1,NKIRAS1,NMBR,NOL4,NPPA,NR6A1,NRG2,NRX3A,NTRK3,NUP93,Nup98,OGDH,OGFOD1,OIT3,OLFM4,OPHN1,OSBP2,OTOA,Oxsr1,P2RX6,Pappa,PAPSS2,Parp16,PATJ,PCDH1,Pcdh15,PC

	DHAC2,PCDHGC5,Pcsk2,PDSS2,Perm1,PID1,PIGK,Pigq,PIK3AP1,PINX1,Pisd,PITPNC1,PITPNM2,PKNOX2,PLA2G1B,PLCB1,PLEKHG1,Plxnd1,Pnpla2,POLA1,PPFIA4,PPM1L,PPP1R12B,PRDM14,PRICKLE2,PRKAR1B,PRKCH,PRKCQ,PRKD1,Prkg1,Proz,PRPF4,prr5,PRRX1,PSEN1,PSTPIP1,PTPN11,PTPRF,Ptprk,PTPRM,PTPRO,PTPRR,PTPRS,PTPRU,RAB4A,RABGAP1L,RAD54B,RALGAPA2,RALYL,Ranbp10,RARS,RASA3,RBBP7,RBM19,Reep1,RETREG3,RFWD3,RGS4,Rgs9,RIN3,Rnpepl1,ROS1,RPAP1,Rptor,RRAGC,Rrbp1,Rxra,SAFB,SAMD11,SARDH,SCNN1B,SCRN1,SDK1,SEC31A,SEMA5B,SERPINA10,SERPINE2,SETD2,SFMBT1,SFRP1,Sh3rf2,Shank3,SLC1A7,Slc22a18,SLC24A3,Slc25a22,slc25a25,SLC26A4,SLC2A12,SLC2A13,slc30a8,SLC39A11,SLC39A12,SLC4A4,SLC5A11,SLC5A12,SLC6A1,SLC6A14,SLC8A1,Slco2a1,smarcal1,SMARCC1,SMYD1,SND1,Snx27,Spsb3,SPSB4,SPTBN1,SRD5A2,ST3GAL3,STAMBPL1,STIM1,STIMATE,STK32C,STX1A,SUSD2,SUSD5,Sv2b,SYN3,SYT6,T,TADA1,TAF1A,TAF7,TBCD,TCEA2,TCEANC2,tcf3,TEAD4,Tedc1,TENM4,tfap2e,THSD7B,THUMPD2,TIRAP,Tkt,Tmc3,Tmcc1,TMEFF2,Tmem47,TMEM59,Tmie,Tmtc1,TNNI2,TNNI3K,TP73,TPCN1,Tpk1,TRABD,Trabd2b,TRAF3,TRAF3IP2,TRAK2,TRHDE,TRIM29,TRIM66,TSNARE1,TSPAN15,TSPAN5,TTC28,Tubgcp2,UMAD1,URB1,USP28,USP40,VASH2,VAT1L,Vav2,VDHAP,VPS33A,WASF2,WDR82,WISP1,WISP3,WRB,WRN,WSCD2,XPR1,XYLT1,YPEL2,Zbtb46,ZNF3
SEX-linked ZZ	
GAL-HUM	*PLCXD3, PARP8, POLI, DCC, Mapk4, CNTFR*
HUM-MAG	*ANKDD1B, TCF4, DCC, Mex3c, CTIF, PIAS2, ST8SIA5, LOXHD1, SIGLEC15, SETBP1, CELF4, Dnai1, DCTN3, CCBE1, ADAMTS12*
MAG-AFR	*PARP8, Map1b, ANKDD1B, DMGDH, CMYA5, Thbs4, TCF4, RAB27B, DCC, Mex3c, CTIF, ZBTB7C, katnal2, PSTPIP2, SETBP1, celf4, nol6, DCTN3, Atp8b2, CCBE1, C1QTNF3, LIFR*
SEX-linked ZW	
GAL-HUM	*FBN2, FSD1L, PLCXD3, PARP8, ST8SIA3, POLI, DCC, Mex3c, Mapk4, CNTFR, ADAMTS12*
HUM-MAG	*TCF4, DCC, Mex3c, MYO5B, CTIF, katnal2, PIAS2, ST8SIA5, LOXHD1, SIGLEC15, SETBP1, CELF4*

MAG-AFR	*FBN2, FSD1L, PARP8, DMGDH, TCF4, RAB27B, DCC, Mex3c, CTIF, ZBTB7C, katnal2, PSTPIP2, SETBP1*

Supplementary Table 10. **Functional Annotation of Genes in Biological Processes.** Genes categorized by their functional annotations according to Gene Ontology (GO) terms. The categories include Learning/Memory, Response to Stimulus, Muscle Contraction/Capacity, Osmoregulation/ION Transport, Hypoxia/ROS, and Starvation/Autophagy, each associated with specific biological functions in banded penguin species.

**Category**	Gene Ontology (GO)
Learning/Memory	GO:0021587, GO:0035176, GO:0008306, GO:0007612, GO:0007613, GO:0007611, GO:0007610, GO:0009612, GO:0009605, GO:0033554, GO:0050896, GO:0031929, GO:0086004, GO:0042594, GO:0051146, GO:0090257, GO:0006874, GO:0072507, GO:0070588, GO:0006816, GO:0055065, GO:0034765, GO:0034220, GO:0006873, GO:0050801, GO:0006812
Response to stimulus	GO:0033554, GO:0031929
Muscle contraction/capacity	GO:0031929, GO:0086004, GO:0042594, GO:0051146, GO:0090257, GO:0034765, GO:0034220
Osmoregulation/ION transport	GO:0006874, GO:0072507, GO:0070588, GO:0006816, GO:0055065, GO:0034765, GO:0034220, GO:0006873, GO:0050801, GO:0006812
HYPOXIA/ROS	GO:0071456, GO:0071453, GO:0033554, GO:1903522
Starvation/autophagy	GO:0031669, GO:0009267, GO:0043502

Supplementary Table 11. Phenotypic associations related with body size difference between Galapagos and Humboldt penguin found under potential selection pressures. Phenotypic traits identified through functional enrichment analysis and their corresponding adjusted p-values.

Phenotype	Phenotype ID	Adjusted p-value
Growth delay	HP:0001510	6.122×10^{-20}
Microcephaly	HP:0000252	6.530×10^{-13}
Short digit	HP:0011927	1.139×10^{-7}
Hypogonadism	HP:0000135	8.446×10^{-6}
Short nose	HP:0003196	1.493×10^{-4}
Hypothyroidism	HP:0000821	1.660×10^{-3}
Short toe	HP:0001831	4.237×10^{-3}
Short finger	HP:0009381	2.111×10^{-2}
Short neck	HP:0001792	3.215×10^{-2}
Small nail	HP:0001792	3.215×10^{-2}

Supplementary Table 12. Rapidly evolving gene families among little blue and banded penguins.

GO ID	Event	Namespace	Name (Child term)	Count p-value	p-value
GO:0000920	Expansion	biological_process	cell separation after cytokinesis	9	8.60E-19
GO:0042803	Expansion	molecular_function	protein homodimerization activity	8	2.73E-13
GO:0000278	Expansion	biological_process	mitotic cell cycle	8	2.25E-12
GO:0003774	Expansion	molecular_function	motor activity	7	1.53E-08
GO:0030182	Expansion	biological_process	neuron differentiation	3	5.86E-05
GO:0001764	Expansion	biological_process	neuron migration	3	1.44E-04
GO:0051225	Expansion	biological_process	spindle assembly	3	8.20E-04
GO:0043087	Expansion	biological_process	regulation of GTPase activity	2	1.14E-02
GO:0007283	Expansion	biological_process	spermatogenesis	5	3.89E-02

Supplementary Table 13. Rapidly evolving gene families between Galápagos and Humboldt penguins

GO ID	Event	Namespace	Name	Count p-value	p-value
GO:0000920	Expansion	biological_process	cell separation after cytokinesis	9	8.60E-19
GO:0042803	Expansion	molecular_function	protein homodimerization activity	8	2.73E-13
GO:0000278	Expansion	biological_process	mitotic cell cycle	8	2.25E-12
GO:0003774	Expansion	molecular_function	motor activity	7	1.53E-08
GO:0007283	Expansion	biological_process	spermatogenesis	5	3.89E-02
GO:0030182	Expansion	biological_process	neuron differentiation	3	5.86E-05
GO:0001764	Expansion	biological_process	neuron migration	3	1.44E-04
GO:0051225	Expansion	biological_process	spindle assembly	3	8.20E-04
GO:0043087	Expansion	biological_process	regulation of GTPase activity	2	1.14E-02

Supplementary Table 14. Rapidly evolving gene families between Magellanic and African penguins.

GO ID	Event	Namespace	Name	Count p-value	p-value
GO:0005200	Expansion	molecular_function	structural constituent of cytoskeleton	69	0.00E+00
GO:0007608	Expansion	biological_process	sensory perception of smell	10	0.00E+00
GO:0005198	Expansion	molecular_function	structural molecule activity	9	0.00E+00
GO:0004984	Expansion	molecular_function	olfactory receptor activity	9	0.00E+00
GO:0007411	Expansion	biological_process	axon guidance	9	0.00E+00
GO:0007283	Expansion	biological_process	spermatogenesis	6	3.57E-04
GO:0043587	Expansion	biological_process	tongue morphogenesis	4	2.23E-08

Supplementary Table 15. Rapidly evolving gene families in Galapagos penguin.

Galápagos					
**GO ID**	**Event**	**Namespace**	**Name (Child term)**	**Count p-value**	**p-value**
GO:0006814	Expansion	biological_process	sodium ion transport	66	9.14E-22
GO:0007165	Expansion	biological_process	signal transduction	65	1.21E-11
GO:0016055	Expansion	biological_process	Wnt signaling pathway	48	2.21E-02
GO:0006511	Expansion	biological_process	ubiquitin-dependent protein catabolic process	43	1.05E-06
GO:0016021	Expansion	cellular_component	integral component of membrane	38	3.15E-24
GO:0007283	Expansion	biological_process	spermatogenesis	36	3.00E-04
GO:0046872	Expansion	molecular_function	metal ion binding	30	1.24E-07
GO:0061038	Expansion	biological_process	uterus morphogenesis	28	7.7E-32
GO:0044826	Expansion	biological_process	viral genome integration into host DNA	28	2.34E-10
GO:0015031	Expansion	biological_process	protein transport	26	4.73E-05
GO:0008380	Expansion	biological_process	RNA splicing	23	1.83E-02
GO:0051932	Expansion	biological_process	synaptic transmission, GABAergic	22	2.73E-08
GO:0006412	Expansion	biological_process	translation	22	1.10E-03
GO:0016020	Expansion	cellular_component	membrane	21	2.68E-05
GO:0045944	Expansion	biological_process	positive regulation of transcription from RNA polymerase II promoter	18	1.34E-04
GO:0006355	Expansion	biological_process	regulation of transcription, DNA-templated	17	7.99E-14
GO:0016032	Expansion	biological_process	viral process	16	3.59E-12
GO:0002250	Expansion	biological_process	adaptive immune response	16	2.69E-11
GO:0016042	Expansion	biological_process	lipid catabolic process	16	1.55E-02
GO:0045747	Expansion	biological_process	positive regulation of Notch signaling pathway	15	3.12E-13

GO:0008270	Expansion	molecular_function	zinc ion binding	15	4.91E-03
GO:0007601	Expansion	biological_process	visual perception	14	5.71E-13
GO:0060676	Expansion	biological_process	ureteric bud formation	14	3.02E-12
GO:0016529	Expansion	cellular_component	sarcoplasmic reticulum	14	3.02E-12
GO:0043488	Expansion	biological_process	regulation of mRNA stability	13	2.73E-05
GO:0006829	Expansion	biological_process	zinc II ion transport	13	2.87E-04
GO:0003723	Expansion	molecular_function	RNA binding	13	1.49E-03
GO:0007018	Expansion	biological_process	microtubule-based movement	13	2.73E-02
GO:0004111	Expansion	molecular_function	creatine kinase activity	12	6.43E-14
GO:0010142	Expansion	biological_process	farnesyl diphosphate biosynthetic process, mevalonate pathway	12	6.43E-14
GO:0016192	Expansion	biological_process	vesicle-mediated transport	12	2.53E-10
GO:0006998	Expansion	biological_process	nuclear envelope organization	12	1.85E-08
GO:0016567	Expansion	biological_process	protein ubiquitination	12	1.81E-07
GO:0042803	Expansion	molecular_function	protein homodimerization activity	12	6.07E-04
GO:0043129	Expansion	biological_process	surfactant homeostasis	11	1.29E-07
GO:0051225	Expansion	biological_process	spindle assembly	11	7.15E-03
GO:0050852	Expansion	biological_process	T cell receptor signaling pathway	11	1.08E-02
GO:0001916	Expansion	biological_process	positive regulation of T cell mediated cytotoxicity	10	2.10E-08
GO:0007015	Expansion	biological_process	actin filament organization	10	2.10E-08
GO:0061408	Expansion	biological_process	positive regulation of transcription from RNA polymerase II promoter in response to heat stress	10	2.21E-05
GO:0005886	Expansion	cellular_component	plasma membrane	10	2.33E-03
GO:0033260	Expansion	biological_process	nuclear DNA replication	9	1.34E-10
GO:0006885	Expansion	biological_process	regulation of pH	9	1.78E-07
GO:0039702	Expansion	biological_process	viral budding via host ESCRT complex	9	7.34E-05
GO:0000920	Expansion	biological_process	cell separation after cytokinesis	9	1.03E-04
GO:0042273	Expansion	biological_process	ribosomal large subunit biogenesis	9	1.74E-02

GO:0005814	Expansion	cellular_component	centriole	8	1.69E-09
GO:0043048	Expansion	biological_process	dolichyl monophosphate biosynthetic process	8	1.69E-09
GO:0002286	Expansion	biological_process	T cell activation involved in immune response	8	1.44E-06
GO:0030018	Expansion	cellular_component	Z disc	8	1.44E-06
GO:0006703	Expansion	biological_process	estrogen biosynthetic process	8	3.11E-06
GO:0004386	Expansion	molecular_function	helicase activity	8	3.25E-05
GO:0051693	Expansion	biological_process	actin filament capping	8	3.25E-05
GO:0044782	Expansion	biological_process	cilium organization	8	5.18E-05
GO:0002009	Expansion	biological_process	morphogenesis of an epithelium	8	7.94E-05
GO:0035774	Expansion	biological_process	positive regulation of insulin secretion involved in cellular response to glucose stimulus	8	1.18E-04
GO:0007264	Expansion	biological_process	small GTPase mediated signal transduction	8	2.32E-04
GO:0014037	Expansion	biological_process	Schwann cell differentiation	8	2.41E-04
GO:0006486	Expansion	biological_process	protein glycosylation	8	2.77E-04
GO:0051897	Expansion	biological_process	positive regulation of protein kinase B signaling	8	4.51E-04
GO:0010469	Expansion	biological_process	regulation of receptor activity	8	6.00E-04
GO:0046983	Expansion	molecular_function	protein dimerization activity	8	6.00E-04
GO:0070207	Expansion	biological_process	protein homotrimerization	8	2.46E-03
GO:0007169	Expansion	biological_process	transmembrane receptor protein tyrosine kinase signaling pathway	8	6.15E-03
GO:0010212	Expansion	biological_process	response to ionizing radiation	8	9.27E-03
GO:0002576	Expansion	biological_process	platelet degranulation	8	2.12E-02
GO:0015718	Expansion	biological_process	monocarboxylic acid transport	8	2.12E-02
GO:0034632	Expansion	molecular_function	retinol transporter activity	7	2.14E-08
GO:0060819	Expansion	biological_process	inactivation of X chromosome by genetic imprinting	7	2.14E-08
GO:0097094	Expansion	biological_process	craniofacial suture morphogenesis	7	2.14E-08
GO:0035498	Expansion	biological_process	carnosine metabolic process	7	2.14E-08
GO:0007276	Expansion	biological_process	gamete generation	7	2.14E-08

GO:0006777	Expansion	biological_process	Mo-molybdopterin cofactor biosynthetic process	7	1.12E-05
GO:0060261	Expansion	biological_process	positive regulation of transcription initiation from RNA polymerase II promoter	7	1.12E-05
GO:0045773	Expansion	biological_process	positive regulation of axon extension	7	1.12E-05
GO:0048845	Expansion	biological_process	venous blood vessel morphogenesis	7	1.82E-04
GO:0055012	Expansion	biological_process	ventricular cardiac muscle cell differentiation	7	1.82E-04
GO:0051382	Expansion	biological_process	kinetochore assembly	7	1.82E-04
GO:0006471	Expansion	biological_process	protein ADP-ribosylation	7	2.74E-04
GO:0072593	Expansion	biological_process	reactive oxygen species metabolic process	7	4.00E-04
GO:0019731	Expansion	biological_process	antibacterial humoral response	7	5.67E-04
GO:0097428	Expansion	biological_process	protein maturation by iron-sulfur cluster transfer	7	9.09E-03
GO:1901796	Expansion	biological_process	regulation of signal transduction by p53 class mediator	7	1.23E-02
GO:0018105	Expansion	biological_process	peptidyl-serine phosphorylation	7	1.42E-02
GO:0001659	Expansion	biological_process	temperature homeostasis	7	1.84E-02
GO:0032012	Expansion	biological_process	regulation of ARF protein signal transduction	7	2.32E-02
GO:0043627	Expansion	biological_process	response to estrogen	7	2.32E-02
GO:0001944	Expansion	biological_process	vasculature development	7	2.32E-02
GO:0006479	Expansion	biological_process	protein methylation	7	2.59E-02
GO:0042182	Expansion	biological_process	ketone catabolic process	6	2.69E-07
GO:0006741	Expansion	biological_process	NADP biosynthetic process	6	2.69E-07
GO:0060078	Expansion	biological_process	regulation of postsynaptic membrane potential	6	2.69E-07
GO:0018401	Expansion	biological_process	peptidyl-proline hydroxylation to 4-hydroxy-L-proline	6	8.22E-05
GO:0046040	Expansion	biological_process	IMP metabolic process	6	1.51E-04
GO:0048729	Expansion	biological_process	tissue morphogenesis	6	9.42E-04
GO:0072573	Expansion	biological_process	tolerance induction to lipopolysaccharide	6	9.42E-04
GO:0035902	Expansion	biological_process	response to immobilization stress	6	9.42E-04
GO:0018095	Expansion	biological_process	protein polyglutamylation	6	3.27E-03

GO:0031098	Expansion	biological_process	stress-activated protein kinase signaling cascade	6	3.56E-03
GO:0051260	Expansion	biological_process	protein homooligomerization	6	7.08E-03
GO:0000921	Expansion	biological_process	septin ring assembly	6	9.86E-03
GO:0002040	Expansion	biological_process	sprouting angiogenesis	6	1.18E-02
GO:0006636	Expansion	biological_process	unsaturated fatty acid biosynthetic process	6	2.18E-02
GO:0006805	Expansion	biological_process	xenobiotic metabolic process	6	2.26E-02
GO:0006357	Expansion	biological_process	regulation of transcription from RNA polymerase II promoter	5	1.02E-17
GO:0045893	Expansion	biological_process	positive regulation of transcription, DNA-templated	5	2.08E-06
GO:0010466	Expansion	biological_process	negative regulation of peptidase activity	5	3.38E-06
GO:0006975	Expansion	biological_process	DNA damage induced protein phosphorylation	5	3.38E-06
GO:0036111	Expansion	biological_process	very long-chain fatty-acyl-CoA metabolic process	5	3.38E-06
GO:0021758	Expansion	biological_process	putamen development	5	3.38E-06
GO:0051639	Expansion	biological_process	actin filament network formation	5	3.38E-06
GO:0010863	Expansion	biological_process	positive regulation of phospholipase C activity	5	3.38E-06
GO:0060158	Expansion	biological_process	phospholipase C-activating dopamine receptor signaling pathway	5	3.38E-06
GO:0010814	Expansion	biological_process	substance P catabolic process	5	3.38E-06
GO:0055085	Expansion	biological_process	transmembrane transport	5	3.04E-05
GO:0035282	Expansion	biological_process	segmentation	5	6.01E-05
GO:0006979	Expansion	biological_process	response to oxidative stress	5	4.55E-04
GO:0051493	Expansion	biological_process	regulation of cytoskeleton organization	5	5.62E-04
GO:0050714	Expansion	biological_process	positive regulation of protein secretion	5	5.62E-04
GO:0004693	Expansion	molecular_function	cyclin-dependent protein serine/threonine kinase activity	5	5.62E-04
GO:1902896	Expansion	biological_process	terminal web assembly	5	5.62E-04
GO:0021559	Expansion	biological_process	trigeminal nerve development	5	9.48E-04
GO:0021644	Expansion	biological_process	vagus nerve morphogenesis	5	9.48E-04
GO:0007156	Expansion	biological_process	homophilic cell adhesion via plasma membrane adhesion molecules	5	1.08E-03
GO:0001504	Expansion	biological_process	neurotransmitter uptake	5	3.20E-03

GO:0043022	Expansion	molecular_function	ribosome binding	5	4.42E-03
GO:0005978	Expansion	biological_process	glycogen biosynthetic process	5	4.42E-03
GO:0042147	Expansion	biological_process	retrograde transport, endosome to Golgi	5	6.17E-03
GO:0006813	Expansion	biological_process	potassium ion transport	5	7.71E-03
GO:0045202	Expansion	cellular_component	synapse	5	1.23E-02
GO:0007517	Expansion	biological_process	muscle organ development	5	1.50E-02
GO:0034097	Expansion	biological_process	response to cytokine	5	2.54E-02
GO:0016358	Expansion	biological_process	dendrite development	4	4.23E-05
GO:0060048	Expansion	biological_process	cardiac muscle contraction	4	4.23E-05
GO:0016486	Expansion	biological_process	peptide hormone processing	4	4.23E-05
GO:0030223	Expansion	biological_process	neutrophil differentiation	4	4.23E-05
GO:0006449	Expansion	biological_process	regulation of translational termination	4	4.23E-05
GO:0019511	Expansion	biological_process	peptidyl-proline hydroxylation	4	4.23E-05
GO:0060322	Expansion	biological_process	head development	4	4.23E-05
GO:0007399	Expansion	biological_process	nervous system development	4	5.10E-04
GO:0008299	Expansion	biological_process	isoprenoid biosynthetic process	4	3.51E-03
GO:0046677	Expansion	biological_process	response to antibiotic	4	1.07E-02
GO:0051496	Expansion	biological_process	positive regulation of stress fiber assembly	4	1.84E-02
GO:0007605	Expansion	biological_process	sensory perception of sound	3	1.69E-07
GO:0008284	Expansion	biological_process	positive regulation of cell proliferation	3	1.52E-02
GO:0007608	Expansion	biological_process	sensory perception of smell	2	2.51E-06
GO:0006367	Expansion	biological_process	transcription initiation from RNA polymerase II promoter	2	5.13E-06
GO:0009617	Expansion	biological_process	response to bacterium	2	1.22E-05
GO:0072560	Expansion	biological_process	type B pancreatic cell maturation	2	6.54E-03
GO:0007603	Expansion	biological_process	phototransduction, visible light	2	6.54E-03
GO:0007626	Expansion	biological_process	locomotory behavior	2	1.81E-02

Supplementary Table 16. Rapidly evolving gene families in Humboldt penguin.

**Humboldt**					
**GO ID**	**Event**	**Namespace**	**Name (Child term)**	**Count p-value**	**p-value**
GO:0006334	Expansion	biological_process	nucleosome assembly	92	1.44E-77
GO:0046872	Expansion	molecular_function	metal ion binding	52	2.69E-03
GO:0007165	Expansion	biological_process	signal transduction	51	1.83E-23
GO:0006511	Expansion	biological_process	ubiquitin-dependent protein catabolic process	36	5.06E-12
GO:0006325	Expansion	biological_process	chromatin organization	34	2.90E-15
GO:0016032	Expansion	biological_process	viral process	31	5.06E-07
GO:0007601	Expansion	biological_process	visual perception	28	8.96E-08
GO:0006357	Expansion	biological_process	regulation of transcription from RNA polymerase II promoter	25	6.15E-07
GO:0003774	Expansion	molecular_function	motor activity	25	3.33E-05
GO:0007525	Expansion	biological_process	somatic muscle development	23	7.31E-14
GO:0000278	Expansion	biological_process	mitotic cell cycle	21	1.77E-07
GO:0016055	Expansion	biological_process	Wnt signaling pathway	19	9.80E-14
GO:0006412	Expansion	biological_process	translation	19	9.73E-06
GO:0060412	Expansion	biological_process	ventricular septum morphogenesis	18	8.02E-05
GO:0006366	Expansion	biological_process	transcription from RNA polymerase II promoter	18	2.06E-03
GO:0006904	Expansion	biological_process	vesicle docking involved in exocytosis	17	2.80E-03
GO:0007283	Expansion	biological_process	spermatogenesis	16	3.23E-15
GO:0015031	Expansion	biological_process	protein transport	16	2.64E-11
GO:0050776	Expansion	biological_process	regulation of immune response	16	1.09E-03
GO:0008270	Expansion	molecular_function	zinc ion binding	16	2.05E-03
GO:0043565	Expansion	molecular_function	sequence-specific DNA binding	14	6.27E-07
GO:0004849	Expansion	molecular_function	uridine kinase activity	12	2.41E-13

GO:0016192	Expansion	biological_process	vesicle-mediated transport	12	2.56E-12
GO:0030100	Expansion	biological_process	regulation of endocytosis	12	1.75E-03
GO:0006635	Expansion	biological_process	fatty acid beta-oxidation	11	5.54E-04
GO:0007275	Expansion	biological_process	multicellular organismal development	11	3.00E-03
GO:0071625	Expansion	biological_process	vocalization behavior	10	1.77E-02
GO:0061097	Expansion	biological_process	regulation of protein tyrosine kinase activity	9	3.58E-10
GO:0005509	Expansion	molecular_function	calcium ion binding	9	5.52E-03
GO:0051259	Expansion	biological_process	protein oligomerization	9	1.81E-02
GO:0045892	Expansion	biological_process	negative regulation of transcription, DNA-templated	8	2.23E-03
GO:0007399	Expansion	biological_process	nervous system development	8	7.91E-03
GO:0006468	Expansion	biological_process	protein phosphorylation	8	1.13E-02
GO:0016021	Expansion	cellular_component	integral component of membrane	7	8.40E-62
GO:0042450	Expansion	biological_process	arginine biosynthetic process via ornithine	7	4.59E-08
GO:0007140	Expansion	biological_process	male meiosis	7	4.59E-08
GO:0045893	Expansion	biological_process	positive regulation of transcription, DNA-templated	7	4.01E-06
GO:0035360	Expansion	biological_process	positive regulation of peroxisome proliferator activated receptor signaling pathway	7	2.29E-05
GO:0006351	Expansion	biological_process	transcription, DNA-templated	7	3.40E-05
GO:0070904	Expansion	biological_process	transepithelial L-ascorbic acid transport	7	1.41E-04
GO:0007179	Expansion	biological_process	transforming growth factor beta receptor signaling pathway	7	5.99E-04
GO:0007034	Expansion	biological_process	vacuolar transport	7	2.55E-03
GO:0071277	Expansion	biological_process	cellular response to calcium ion	7	5.17E-03
GO:0006890	Expansion	biological_process	retrograde vesicle-mediated transport, Golgi to ER	7	1.63E-02
GO:0016567	Expansion	biological_process	protein ubiquitination	6	2.36E-13
GO:0045944	Expansion	biological_process	positive regulation of transcription from RNA polymerase II promoter	6	6.02E-13
GO:0008380	Expansion	biological_process	RNA splicing	6	7.72E-11
GO:0042851	Expansion	biological_process	L-alanine metabolic process	6	5.17E-07

GO:0000395	Expansion	biological_process	mRNA 5'-splice site recognition	6	5.17E-07
GO:0032436	Expansion	biological_process	positive regulation of proteasomal ubiquitin-dependent protein catabolic process	6	5.17E-07
GO:0044065	Expansion	biological_process	regulation of respiratory system process	6	5.17E-07
GO:0070329	Expansion	biological_process	tRNA seleno-modification	6	5.17E-07
GO:0048147	Expansion	biological_process	negative regulation of fibroblast proliferation	6	5.17E-07
GO:1990573	Expansion	biological_process	potassium ion import across plasma membrane	6	5.17E-07
GO:0005886	Expansion	cellular_component	plasma membrane	6	5.39E-06
GO:0042060	Expansion	biological_process	wound healing	6	3.06E-05
GO:0008063	Expansion	biological_process	Toll signaling pathway	6	7.49E-05
GO:0009303	Expansion	biological_process	rRNA transcription	6	7.49E-05
GO:0010737	Expansion	biological_process	protein kinase A signaling	6	1.50E-04
GO:0016267	Expansion	biological_process	O-glycan processing, core 1	6	2.74E-04
GO:0017185	Expansion	biological_process	peptidyl-lysine hydroxylation	6	1.12E-03
GO:0052689	Expansion	molecular_function	carboxylic ester hydrolase activity	6	1.56E-02
GO:0007605	Expansion	biological_process	sensory perception of sound	5	5.77E-07
GO:0006543	Expansion	biological_process	glutamine catabolic process	5	5.82E-06
GO:0000098	Expansion	biological_process	sulfur amino acid catabolic process	5	5.82E-06
GO:0070120	Expansion	biological_process	ciliary neurotrophic factor-mediated signaling pathway	5	5.82E-06
GO:0007188	Expansion	biological_process	adenylate cyclase-modulating G-protein coupled receptor signaling pathway	5	5.82E-06
GO:0006754	Expansion	biological_process	ATP biosynthetic process	5	5.82E-06
GO:0033043	Expansion	biological_process	regulation of organelle organization	5	5.82E-06
GO:0034653	Expansion	biological_process	retinoic acid catabolic process	5	5.82E-06
GO:0015379	Expansion	molecular_function	potassium:chloride symporter activity	5	5.82E-06
GO:0040040	Expansion	biological_process	thermosensory behavior	5	5.82E-06
GO:2000036	Expansion	biological_process	regulation of stem cell maintenance	5	5.82E-06
GO:0010971	Expansion	biological_process	positive regulation of G2/M transition of mitotic cell cycle	5	5.82E-06

GO:1901388	Expansion	biological_process	regulation of transforming growth factor beta activation	5	3.18E-05
GO:0007186	Expansion	biological_process	G-protein coupled receptor signaling pathway	5	1.96E-04
GO:0007156	Expansion	biological_process	homophilic cell adhesion via plasma membrane adhesion molecules	5	2.81E-04
GO:0046034	Expansion	biological_process	ATP metabolic process	5	5.05E-04
GO:0007155	Expansion	biological_process	cell adhesion	5	6.55E-04
GO:0061304	Expansion	biological_process	retinal blood vessel morphogenesis	5	9.20E-04
GO:0007158	Expansion	biological_process	neuron cell-cell adhesion	5	9.20E-04
GO:0035239	Expansion	biological_process	tube morphogenesis	5	9.20E-04
GO:0036324	Expansion	biological_process	vascular endothelial growth factor receptor-2 signaling pathway	5	1.54E-03
GO:0006680	Expansion	biological_process	glucosylceramide catabolic process	5	2.40E-03
GO:0030050	Expansion	biological_process	vesicle transport along actin filament	5	2.40E-03
GO:0022604	Expansion	biological_process	regulation of cell morphogenesis	5	3.55E-03
GO:2001242	Expansion	biological_process	regulation of intrinsic apoptotic signaling pathway	5	5.03E-03
GO:0051290	Expansion	biological_process	protein heterotetramerization	5	5.03E-03
GO:2000582	Expansion	biological_process	positive regulation of ATP-dependent microtubule motor activity, plus-end-directed	5	5.03E-03
GO:0008542	Expansion	biological_process	visual learning	5	5.37E-03
GO:0000028	Expansion	biological_process	ribosomal small subunit assembly	5	6.87E-03
GO:0010842	Expansion	biological_process	retina layer formation	5	6.87E-03
GO:0051568	Expansion	biological_process	histone H3-K4 methylation	5	6.87E-03
GO:0006670	Expansion	biological_process	sphingosine metabolic process	5	6.87E-03
GO:0007178	Expansion	biological_process	transmembrane receptor protein serine/threonine kinase signaling pathway	5	6.87E-03
GO:1903358	Expansion	biological_process	regulation of Golgi organization	5	6.87E-03
GO:0008235	Expansion	molecular_function	metalloexopeptidase activity	5	9.10E-03
GO:0008589	Expansion	biological_process	regulation of smoothened signaling pathway	5	1.17E-02
GO:0046470	Expansion	biological_process	phosphatidylcholine metabolic process	5	1.48E-02
GO:0005506	Expansion	molecular_function	iron ion binding	5	1.83E-02

GO:0003700	Expansion	molecular_function	sequence-specific DNA binding transcription factor activity	5	2.22E-02
GO:0051787	Expansion	molecular_function	misfolded protein binding	4	6.52E-05
GO:0075732	Expansion	biological_process	viral penetration into host nucleus	4	6.52E-05
GO:0007214	Expansion	biological_process	gamma-aminobutyric acid signaling pathway	4	5.15E-03
GO:0007522	Expansion	biological_process	visceral muscle development	4	5.15E-03
GO:0050690	Expansion	biological_process	regulation of defense response to virus by virus	4	5.15E-03
GO:0060348	Expansion	biological_process	bone development	4	5.15E-03
GO:0005737	Expansion	cellular_component	cytoplasm	3	3.05E-04
GO:0006477	Expansion	biological_process	protein sulfation	3	7.29E-04
GO:0030201	Expansion	biological_process	heparan sulfate proteoglycan metabolic process	3	7.29E-04
GO:0015187	Expansion	molecular_function	glycine transmembrane transporter activity	3	7.29E-04
GO:0030278	Expansion	biological_process	regulation of ossification	3	7.29E-04
GO:1900746	Expansion	biological_process	regulation of vascular endothelial growth factor signaling pathway	3	6.04E-03
GO:0043312	Expansion	biological_process	neutrophil degranulation	3	1.95E-02
GO:0007264	Expansion	biological_process	small GTPase mediated signal transduction	2	1.53E-09
GO:0005634	Expansion	cellular_component	nucleus	2	1.84E-09
GO:0050852	Expansion	biological_process	T cell receptor signaling pathway	2	2.11E-08
GO:0048511	Expansion	biological_process	rhythmic process	2	2.19E-03
GO:0010468	Expansion	biological_process	regulation of gene expression	2	6.90E-03
GO:0048384	Expansion	biological_process	retinoic acid receptor signaling pathway	2	8.12E-03
GO:0016322	Expansion	biological_process	neuron remodeling	2	8.12E-03
GO:0010762	Expansion	biological_process	regulation of fibroblast migration	2	8.12E-03
GO:0060968	Expansion	biological_process	regulation of gene silencing	2	8.12E-03
GO:0035176	Expansion	biological_process	social behavior	2	2.04E-02

Supplementary Table 17. Rapidly evolving gene families in Magellanic penguin.

Magellanic					
**GO ID**	**Event**	**Namespace**	**Name (Child term)**	**Count p-value**	**p-value**
GO:0006334	Expansion	biological_process	nucleosome assembly	92	2.38E-94
GO:0005200	Expansion	molecular_function	structural constituent of cytoskeleton	82	3.81E-44
GO:0006355	Expansion	biological_process	regulation of transcription, DNA-templated	57	1.96E-02
GO:0006511	Expansion	biological_process	ubiquitin-dependent protein catabolic process	46	8.20E-03
GO:0006325	Expansion	biological_process	chromatin organization	34	3.95E-20
GO:0016032	Expansion	biological_process	viral process	29	2.51E-03
GO:0016055	Expansion	biological_process	Wnt signaling pathway	27	3.53E-04
GO:0003774	Expansion	molecular_function	motor activity	25	5.96E-08
GO:0015031	Expansion	biological_process	protein transport	25	4.34E-03
GO:0050776	Expansion	biological_process	regulation of immune response	22	1.50E-09
GO:0000122	Expansion	biological_process	negative regulation of transcription from RNA polymerase II promoter	22	4.70E-03
GO:0061038	Expansion	biological_process	uterus morphogenesis	21	2.41E-20
GO:0006890	Expansion	biological_process	retrograde vesicle-mediated transport, Golgi to ER	21	3.19E-04
GO:0006412	Expansion	biological_process	translation	21	1.97E-02
GO:0046872	Expansion	molecular_function	metal ion binding	20	8.38E-08
GO:0030433	Expansion	biological_process	ER-associated ubiquitin-dependent protein catabolic process	20	6.09E-03
GO:0007283	Expansion	biological_process	spermatogenesis	19	2.52E-02
GO:0007525	Expansion	biological_process	somatic muscle development	18	2.05E-11
GO:0060412	Expansion	biological_process	ventricular septum morphogenesis	18	6.82E-07
GO:2000300	Expansion	biological_process	regulation of synaptic vesicle exocytosis	16	2.12E-06
GO:0006357	Expansion	biological_process	regulation of transcription from RNA polymerase II promoter	15	2.20E-06
GO:0002244	Expansion	biological_process	hematopoietic progenitor cell differentiation	14	7.75E-09

GO:0048741	Expansion	biological_process	skeletal muscle fiber development	14	2.19E-07
GO:0008380	Expansion	biological_process	RNA splicing	14	4.01E-03
GO:0007165	Expansion	biological_process	signal transduction	13	5.13E-35
GO:0003677	Expansion	molecular_function	DNA binding	13	1.08E-03
GO:0006904	Expansion	biological_process	vesicle docking involved in exocytosis	13	4.46E-03
GO:0004111	Expansion	molecular_function	creatine kinase activity	12	3.37E-15
GO:0010142	Expansion	biological_process	farnesyl diphosphate biosynthetic process, mevalonate pathway	12	3.37E-15
GO:0004849	Expansion	molecular_function	uridine kinase activity	12	3.37E-15
GO:0016567	Expansion	biological_process	protein ubiquitination	12	6.74E-05
GO:0030100	Expansion	biological_process	regulation of endocytosis	12	8.29E-05
GO:0007601	Expansion	biological_process	visual perception	11	2.15E-10
GO:0030335	Expansion	biological_process	positive regulation of cell migration	11	5.12E-06
GO:0006366	Expansion	biological_process	transcription from RNA polymerase II promoter	11	2.60E-03
GO:0007015	Expansion	biological_process	actin filament organization	10	1.99E-09
GO:0051321	Expansion	biological_process	meiotic cell cycle	10	1.07E-05
GO:0061097	Expansion	biological_process	regulation of protein tyrosine kinase activity	9	1.48E-11
GO:0016192	Expansion	biological_process	vesicle-mediated transport	9	1.31E-08
GO:0045944	Expansion	biological_process	positive regulation of transcription from RNA polymerase II promoter	9	4.04E-06
GO:0051607	Expansion	biological_process	defense response to virus	9	1.10E-05
GO:0038123	Expansion	biological_process	toll-like receptor TLR1:TLR2 signaling pathway	8	2.41E-10
GO:0007605	Expansion	biological_process	sensory perception of sound	8	5.08E-03
GO:0005634	Expansion	cellular_component	nucleus	8	5.49E-03
GO:0050852	Expansion	biological_process	T cell receptor signaling pathway	8	1.41E-02
GO:0006897	Expansion	biological_process	endocytosis	8	1.45E-02
GO:0016021	Expansion	cellular_component	integral component of membrane	7	2.85E-40
GO:0042450	Expansion	biological_process	arginine biosynthetic process via ornithine	7	3.90E-09
GO:0034632	Expansion	molecular_function	retinol transporter activity	7	3.90E-09

GO:0060819	Expansion	biological_process	inactivation of X chromosome by genetic imprinting	7	3.90E-09
GO:0007140	Expansion	biological_process	male meiosis	7	3.90E-09
GO:0035498	Expansion	biological_process	carnosine metabolic process	7	3.90E-09
GO:0007276	Expansion	biological_process	gamete generation	7	3.90E-09
GO:0010818	Expansion	biological_process	T cell chemotaxis	7	1.24E-07
GO:0035360	Expansion	biological_process	positive regulation of peroxisome proliferator activated receptor signaling pathway	7	2.24E-06
GO:0006777	Expansion	biological_process	Mo-molybdopterin cofactor biosynthetic process	7	2.24E-06
GO:0060261	Expansion	biological_process	positive regulation of transcription initiation from RNA polymerase II promoter	7	2.24E-06
GO:0045773	Expansion	biological_process	positive regulation of axon extension	7	2.24E-06
GO:0070904	Expansion	biological_process	transepithelial L-ascorbic acid transport	7	1.51E-05
GO:0048845	Expansion	biological_process	venous blood vessel morphogenesis	7	4.00E-05
GO:0055012	Expansion	biological_process	ventricular cardiac muscle cell differentiation	7	4.00E-05
GO:0051382	Expansion	biological_process	kinetochore assembly	7	4.00E-05
GO:0004386	Expansion	molecular_function	helicase activity	7	6.15E-05
GO:0006471	Expansion	biological_process	protein ADP-ribosylation	7	6.15E-05
GO:0019731	Expansion	biological_process	antibacterial humoral response	7	1.32E-04
GO:0002009	Expansion	biological_process	morphogenesis of an epithelium	7	1.32E-04
GO:0007034	Expansion	biological_process	vacuolar transport	7	3.44E-04
GO:0071277	Expansion	biological_process	cellular response to calcium ion	7	7.61E-04
GO:0097428	Expansion	biological_process	protein maturation by iron-sulfur cluster transfer	7	2.65E-03
GO:1901796	Expansion	biological_process	regulation of signal transduction by p53 class mediator	7	3.72E-03
GO:0018105	Expansion	biological_process	peptidyl-serine phosphorylation	7	4.36E-03
GO:0001659	Expansion	biological_process	temperature homeostasis	7	5.87E-03
GO:0043627	Expansion	biological_process	response to estrogen	7	7.71E-03
GO:0001944	Expansion	biological_process	vasculature development	7	7.71E-03
GO:0006479	Expansion	biological_process	protein methylation	7	8.76E-03

GO:0019058	Expansion	biological_process	viral life cycle	7	1.54E-02
GO:0009408	Expansion	biological_process	response to heat	7	1.88E-02
GO:0045892	Expansion	biological_process	negative regulation of transcription, DNA-templated	7	2.50E-02
GO:0016485	Expansion	biological_process	protein processing	7	2.67E-02
GO:0060078	Expansion	biological_process	regulation of postsynaptic membrane potential	6	6.28E-08
GO:0005886	Expansion	cellular_component	plasma membrane	6	1.23E-03
GO:0052689	Expansion	molecular_function	carboxylic ester hydrolase activity	6	3.28E-03
GO:0043565	Expansion	molecular_function	sequence-specific DNA binding	6	1.58E-02
GO:0003016	Expansion	biological_process	respiratory system process	5	1.01E-06
GO:0045429	Expansion	biological_process	positive regulation of nitric oxide biosynthetic process	5	1.01E-06
GO:0007188	Expansion	biological_process	adenylate cyclase-modulating G-protein coupled receptor signaling pathway	5	1.01E-06
GO:0010863	Expansion	biological_process	positive regulation of phospholipase C activity	5	1.01E-06
GO:0060158	Expansion	biological_process	phospholipase C-activating dopamine receptor signaling pathway	5	1.01E-06
GO:0010971	Expansion	biological_process	positive regulation of G2/M transition of mitotic cell cycle	5	1.01E-06
GO:0008270	Expansion	molecular_function	zinc ion binding	5	1.71E-05
GO:0046034	Expansion	biological_process	ATP metabolic process	5	9.81E-05
GO:0006937	Expansion	biological_process	regulation of muscle contraction	5	1.84E-04
GO:0021978	Expansion	biological_process	telencephalon regionalization	5	1.84E-04
GO:0060510	Expansion	biological_process	Type II pneumocyte differentiation	5	1.84E-04
GO:2000582	Expansion	biological_process	positive regulation of ATP-dependent microtubule motor activity, plus-end-directed	5	1.13E-03
GO:0001504	Expansion	biological_process	neurotransmitter uptake	5	1.13E-03
GO:1903358	Expansion	biological_process	regulation of Golgi organization	5	1.59E-03
GO:0071805	Expansion	biological_process	potassium ion transmembrane transport	5	2.16E-03
GO:0045202	Expansion	cellular_component	synapse	5	4.74E-03
GO:0046982	Expansion	molecular_function	protein heterodimerization activity	5	7.30E-03
GO:0006936	Expansion	biological_process	muscle contraction	5	1.06E-02

GO:0031098	Expansion	biological_process	stress-activated protein kinase signaling cascade	5	1.20E-02
GO:0032434	Expansion	biological_process	regulation of proteasomal ubiquitin-dependent protein catabolic process	5	1.25E-02
GO:0016239	Expansion	biological_process	positive regulation of macroautophagy	5	1.47E-02
GO:0040020	Expansion	biological_process	regulation of meiotic nuclear division	4	1.61E-05
GO:0006434	Expansion	biological_process	seryl-tRNA aminoacylation	4	1.61E-05
GO:0060048	Expansion	biological_process	cardiac muscle contraction	4	1.61E-05
GO:0061563	Expansion	biological_process	trigeminal ganglion structural organization	4	1.61E-05
GO:0019511	Expansion	biological_process	peptidyl-proline hydroxylation	4	1.61E-05
GO:0060322	Expansion	biological_process	head development	4	1.61E-05
GO:0001975	Expansion	biological_process	response to amphetamine	4	8.69E-04
GO:0007214	Expansion	biological_process	gamma-aminobutyric acid signaling pathway	4	1.47E-03
GO:0007522	Expansion	biological_process	visceral muscle development	4	1.47E-03
GO:0031957	Expansion	molecular_function	very long-chain fatty acid-CoA ligase activity	4	3.37E-03
GO:0007156	Expansion	biological_process	homophilic cell adhesion via plasma membrane adhesion molecules	4	4.10E-03
GO:0007399	Expansion	biological_process	nervous system development	4	5.67E-03
GO:0008217	Expansion	biological_process	regulation of blood pressure	4	8.42E-03
GO:0051496	Expansion	biological_process	positive regulation of stress fiber assembly	4	8.42E-03
GO:0048515	Expansion	biological_process	spermatid differentiation	4	8.42E-03
GO:0061032	Expansion	biological_process	visceral serous pericardium development	4	1.08E-02
GO:0016226	Expansion	biological_process	iron-sulfur cluster assembly	4	2.36E-02
GO:0030278	Expansion	biological_process	regulation of ossification	3	2.56E-04
GO:0006468	Expansion	biological_process	protein phosphorylation	3	2.59E-03
GO:0014029	Expansion	biological_process	neural crest formation	3	1.03E-02
GO:0061551	Expansion	biological_process	trigeminal ganglion development	3	1.03E-02
GO:0048168	Expansion	biological_process	regulation of neuronal synaptic plasticity	3	1.45E-02
GO:0007179	Expansion	biological_process	transforming growth factor beta receptor signaling pathway	2	7.73E-05
GO:0048384	Expansion	biological_process	retinoic acid receptor signaling pathway	2	4.04E-03

GO:0072560	Expansion	biological_process	type B pancreatic cell maturation	2	4.04E-03
GO:0007603	Expansion	biological_process	phototransduction, visible light	2	4.04E-03
GO:0010762	Expansion	biological_process	regulation of fibroblast migration	2	4.04E-03
GO:0048511	Expansion	biological_process	rhythmic process	2	2.28E-02

Supplementary Table 18. Rapidly evolving gene families in African penguin.

**African**					
**GO ID**	**Event**	**Namespace**	**Name (Child term)**	**Count p-value**	**p-value**
GO:0044826	Expansion	biological_process	viral genome integration into host DNA	33	1.34E-30
GO:0042060	Expansion	biological_process	wound healing	24	3.75E-09
GO:0004984	Expansion	molecular_function	olfactory receptor activity	20	7.60E-14
GO:0051291	Expansion	biological_process	protein heterooligomerization	18	6.46E-19
GO:0007608	Expansion	biological_process	sensory perception of smell	18	4.90E-06
GO:0051453	Expansion	biological_process	regulation of intracellular pH	17	1.07E-21
GO:0002250	Expansion	biological_process	adaptive immune response	16	4.37E-19
GO:0007155	Expansion	biological_process	cell adhesion	15	3.60E-05
GO:0007275	Expansion	biological_process	multicellular organismal development	15	1.05E-03
GO:0006313	Expansion	biological_process	transposition, DNA-mediated	14	5.41E-16
GO:0005198	Expansion	molecular_function	structural molecule activity	14	1.12E-07
GO:0006355	Expansion	biological_process	regulation of transcription, DNA-templated	14	3.13E-02
GO:0050661	Expansion	molecular_function	NADP binding	13	2.15E-20
GO:0051932	Expansion	biological_process	synaptic transmission, GABAergic	13	3.90E-08
GO:0051412	Expansion	biological_process	response to corticosterone	12	3.43E-14
GO:0005576	Expansion	cellular_component	extracellular region	12	1.48E-02

GO:0016021	Expansion	cellular_component	integral component of membrane	10	1.91E-10
GO:0045110	Expansion	biological_process	intermediate filament bundle assembly	9	4.86E-15
GO:0046513	Expansion	biological_process	ceramide biosynthetic process	9	9.93E-13
GO:0007411	Expansion	biological_process	axon guidance	9	6.69E-09
GO:0019068	Expansion	biological_process	virion assembly	9	1.38E-07
GO:0046081	Expansion	biological_process	dUTP catabolic process	8	1.98E-13
GO:0004569	Expansion	molecular_function	glycoprotein endo-alpha-1,2-mannosidase activity	8	1.98E-13
GO:0033273	Expansion	biological_process	response to vitamin	8	1.98E-13
GO:0019731	Expansion	biological_process	antibacterial humoral response	8	1.85E-08
GO:0010951	Expansion	biological_process	negative regulation of endopeptidase activity	8	2.00E-05
GO:0007519	Expansion	biological_process	skeletal muscle tissue development	8	2.35E-05
GO:0006616	Expansion	biological_process	SRP-dependent cotranslational protein targeting to membrane, translocation	8	5.68E-05
GO:0051603	Expansion	biological_process	proteolysis involved in cellular protein catabolic process	8	1.22E-04
GO:0006487	Expansion	biological_process	protein N-linked glycosylation	8	3.58E-04
GO:0015747	Expansion	biological_process	urate transport	7	8.86E-10
GO:0005977	Expansion	biological_process	glycogen metabolic process	7	9.07E-06
GO:0032012	Expansion	biological_process	regulation of ARF protein signal transduction	7	5.45E-05
GO:0070723	Expansion	biological_process	response to cholesterol	6	2.16E-09
GO:0035902	Expansion	biological_process	response to immobilization stress	6	1.97E-06
GO:0030488	Expansion	biological_process	tRNA methylation	6	3.38E-03
GO:0042246	Expansion	biological_process	tissue regeneration	6	3.38E-03
GO:0006457	Expansion	biological_process	protein folding	6	2.36E-02
GO:0007165	Expansion	biological_process	signal transduction	5	1.35E-15
GO:0045657	Expansion	biological_process	positive regulation of monocyte differentiation	5	1.25E-08
GO:2000036	Expansion	biological_process	regulation of stem cell maintenance	5	1.25E-08
GO:0050916	Expansion	biological_process	sensory perception of sweet taste	5	2.76E-06

GO:0008589	Expansion	biological_process	regulation of smoothened signaling pathway	5	5.66E-05
GO:0016973	Expansion	biological_process	poly(A)+ mRNA export from nucleus	5	7.64E-05
GO:0005344	Expansion	molecular_function	oxygen transporter activity	5	1.64E-03
GO:0007525	Expansion	biological_process	somatic muscle development	5	4.49E-03
GO:0021537	Expansion	biological_process	telencephalon development	5	1.11E-02
GO:0043687	Expansion	biological_process	post-translational protein modification	5	1.27E-02
GO:0045766	Expansion	biological_process	positive regulation of angiogenesis	4	4.85E-07
GO:0002246	Expansion	biological_process	wound healing involved in inflammatory response	4	4.85E-07
GO:0046677	Expansion	biological_process	response to antibiotic	4	1.95E-04
GO:0060314	Expansion	biological_process	regulation of ryanodine-sensitive calcium-release channel activity	4	3.74E-04
GO:0043587	Expansion	biological_process	tongue morphogenesis	4	1.27E-03
GO:0005096	Expansion	molecular_function	GTPase activator activity	4	6.00E-03
GO:0030335	Expansion	biological_process	positive regulation of cell migration	4	1.13E-02
GO:0000038	Expansion	biological_process	very long-chain fatty acid metabolic process	4	1.73E-02
GO:1900746	Expansion	biological_process	regulation of vascular endothelial growth factor signaling pathway	3	1.77E-04
GO:0016055	Expansion	biological_process	Wnt signaling pathway	2	1.30E-07
GO:0007601	Expansion	biological_process	visual perception	2	4.66E-07
GO:0009952	Expansion	biological_process	anterior/posterior pattern specification	2	4.05E-03
GO:0042136	Expansion	biological_process	neurotransmitter biosynthetic process	2	6.57E-03
GO:0045893	Expansion	biological_process	positive regulation of transcription, DNA-templated	2	1.24E-02

Supplementary Table 19. Rapidly losses gene families among Little blue and banded penguins.

**Little Blue vs Banden Penguin**					
**GO ID**	**Event**	**Namespace**	**Name (Child term)**	**Count p-value**	**p-value**

GO:0007608	Contraction	biological_process	sensory perception of smell	28	1.12E-08
GO:0016021	Contraction	cellular_component	integral component of membrane	28	2.77E-08
GO:0016032	Contraction	biological_process	viral process	21	2.43E-02
GO:0007165	Contraction	biological_process	signal transduction	19	3.46E-14
GO:0005200	Contraction	molecular_function	structural constituent of cytoskeleton	19	2.02E-03
GO:0051453	Contraction	biological_process	regulation of intracellular pH	17	3.18E-18
GO:0006511	Contraction	biological_process	ubiquitin-dependent protein catabolic process	17	4.56E-06
GO:0070062	Contraction	cellular_component	extracellular exosome	16	1.30E-13
GO:0007283	Contraction	biological_process	spermatogenesis	16	1.34E-03
GO:0007155	Contraction	biological_process	cell adhesion	15	3.44E-03
GO:0006355	Contraction	biological_process	regulation of transcription, DNA-templated	14	5.84E-05
GO:0007586	Contraction	biological_process	digestion	13	1.51E-14
GO:0000278	Contraction	biological_process	mitotic cell cycle	13	2.54E-06
GO:0046872	Contraction	molecular_function	metal ion binding	13	1.29E-05
GO:0007186	Contraction	biological_process	G-protein coupled receptor signaling pathway	13	2.78E-02
GO:0006313	Contraction	biological_process	transposition, DNA-mediated	12	1.76E-10
GO:0030335	Contraction	biological_process	positive regulation of cell migration	11	7.57E-08
GO:0006357	Contraction	biological_process	regulation of transcription from RNA polymerase II promoter	11	3.85E-04
GO:0044826	Contraction	biological_process	viral genome integration into host DNA	11	1.08E-03
GO:0046685	Contraction	biological_process	response to arsenic-containing substance	10	1.20E-14
GO:0016055	Contraction	biological_process	Wnt signaling pathway	10	3.01E-06
GO:0033260	Contraction	biological_process	nuclear DNA replication	9	3.06E-13
GO:0016192	Contraction	biological_process	vesicle-mediated transport	9	2.17E-04
GO:0042273	Contraction	biological_process	ribosomal large subunit biogenesis	9	2.65E-04
GO:0002098	Contraction	biological_process	tRNA wobble uridine modification	9	6.49E-04

GO:0005975	Contraction	biological_process	carbohydrate metabolic process	9	3.69E-03
GO:0004984	Contraction	molecular_function	olfactory receptor activity	9	9.15E-03
GO:0006904	Contraction	biological_process	vesicle docking involved in exocytosis	9	1.16E-02
GO:0046081	Contraction	biological_process	dUTP catabolic process	8	7.74E-12
GO:0038123	Contraction	biological_process	toll-like receptor TLR1:TLR2 signaling pathway	8	7.74E-12
GO:0051786	Contraction	molecular_function	all-trans-retinol 13,14-reductase activity	8	6.69E-11
GO:0006865	Contraction	biological_process	amino acid transport	8	1.31E-07
GO:0031175	Contraction	biological_process	neuron projection development	8	3.50E-03
GO:0019432	Contraction	biological_process	triglyceride biosynthetic process	8	3.50E-03
GO:0044319	Contraction	biological_process	wound healing, spreading of cells	8	4.18E-03
GO:0032496	Contraction	biological_process	response to lipopolysaccharide	8	2.43E-02
GO:0006915	Contraction	biological_process	apoptotic process	8	2.80E-02
GO:0006068	Contraction	biological_process	ethanol catabolic process	7	1.94E-10
GO:0007157	Contraction	biological_process	heterophilic cell-cell adhesion via plasma membrane cell adhesion molecules	7	1.94E-10
GO:0051492	Contraction	biological_process	regulation of stress fiber assembly	7	1.54E-06
GO:0051289	Contraction	biological_process	protein homotetramerization	7	2.67E-03
GO:0043065	Contraction	biological_process	positive regulation of apoptotic process	6	2.44E-02
GO:0070374	Contraction	biological_process	positive regulation of ERK1 and ERK2 cascade	6	2.60E-02
GO:0007601	Contraction	biological_process	visual perception	5	8.60E-09
GO:0003334	Contraction	biological_process	keratinocyte development	5	1.20E-07
GO:0016592	Contraction	cellular_component	mediator complex	5	1.20E-07
GO:0071333	Contraction	biological_process	cellular response to glucose stimulus	5	1.20E-07
GO:0035282	Contraction	biological_process	segmentation	5	2.31E-06
GO:0046883	Contraction	biological_process	regulation of hormone secretion	5	2.45E-05
GO:0035751	Contraction	biological_process	regulation of lysosomal lumen pH	5	2.45E-05

GO:0006776	Contraction	biological_process	vitamin A metabolic process	5	2.45E-05
GO:0043457	Contraction	biological_process	regulation of cellular respiration	5	2.45E-05
GO:0021978	Contraction	biological_process	telencephalon regionalization	5	2.45E-05
GO:0017188	Contraction	molecular_function	aspartate N-acetyltransferase activity	5	2.45E-05
GO:0030208	Contraction	biological_process	dermatan sulfate biosynthetic process	5	2.45E-05
GO:0030672	Contraction	cellular_component	synaptic vesicle membrane	5	2.45E-05
GO:0016567	Contraction	biological_process	protein ubiquitination	5	4.40E-05
GO:0071805	Contraction	biological_process	potassium ion transmembrane transport	5	3.31E-04
GO:0050871	Contraction	biological_process	positive regulation of B cell activation	5	4.50E-04
GO:0043523	Contraction	biological_process	regulation of neuron apoptotic process	5	1.25E-03
GO:0043149	Contraction	biological_process	stress fiber assembly	5	1.91E-03
GO:0009791	Contraction	biological_process	post-embryonic development	5	1.91E-03
GO:0006364	Contraction	biological_process	rRNA processing	5	2.04E-03
GO:0031514	Contraction	cellular_component	motile cilium	5	3.30E-03
GO:0046415	Contraction	biological_process	urate metabolic process	5	1.12E-02
GO:0035567	Contraction	biological_process	non-canonical Wnt signaling pathway	5	1.68E-02
GO:0016338	Contraction	biological_process	calcium-independent cell-cell adhesion via plasma membrane cell-adhesion molecules	5	2.01E-02
GO:0007219	Contraction	biological_process	Notch signaling pathway	5	2.01E-02
GO:0045663	Contraction	biological_process	positive regulation of myoblast differentiation	4	2.94E-06
GO:0035220	Contraction	biological_process	wing disc development	4	2.94E-06
GO:0045944	Contraction	biological_process	positive regulation of transcription from RNA polymerase II promoter	4	1.47E-05
GO:0061630	Contraction	molecular_function	ubiquitin protein ligase activity	4	3.01E-04
GO:0006556	Contraction	biological_process	S-adenosylmethionine biosynthetic process	4	3.01E-04
GO:2001256	Contraction	biological_process	regulation of store-operated calcium entry	4	1.94E-03

GO:2000582	Contraction	biological_process	positive regulation of ATP-dependent microtubule motor activity, plus-end-directed	4	1.94E-03
GO:0039702	Contraction	biological_process	viral budding via host ESCRT complex	4	1.55E-02
GO:0005096	Contraction	molecular_function	GTPase activator activity	4	2.46E-02
GO:0010867	Contraction	biological_process	positive regulation of triglyceride biosynthetic process	3	3.25E-03
GO:0048168	Contraction	biological_process	regulation of neuronal synaptic plasticity	3	4.68E-03
GO:0045599	Contraction	biological_process	negative regulation of fat cell differentiation	3	8.46E-03
GO:0005044	Contraction	molecular_function	scavenger receptor activity	3	1.64E-02
GO:0007017	Contraction	biological_process	microtubule-based process	3	3.12E-02
GO:0005886	Contraction	cellular_component	plasma membrane	2	7.77E-04
GO:0010092	Contraction	biological_process	specification of organ identity	2	1.74E-03
GO:0007399	Contraction	biological_process	nervous system development	2	1.26E-02

Supplementary Table 20. Rapidly losses gene families between Galápagos and Humboldt penguins

Galápagos Humboldt					
**GO ID**	**Event**	**Namespace**	**Name (Child term)**	**Count p-value**	**p-value**
GO:0007608	Contraction	biological_process	sensory perception of smell	28	1.12E-08
GO:0016021	Contraction	cellular_component	integral component of membrane	28	2.77E-08
GO:0016032	Contraction	biological_process	viral process	21	2.43E-02
GO:0007165	Contraction	biological_process	signal transduction	19	3.46E-14
GO:0005200	Contraction	molecular_function	structural constituent of cytoskeleton	19	2.02E-03
GO:0051453	Contraction	biological_process	regulation of intracellular pH	17	3.18E-18
GO:0006511	Contraction	biological_process	ubiquitin-dependent protein catabolic process	17	4.56E-06

GO:0070062	Contraction	cellular_component	extracellular exosome	16	1.30E-13
GO:0007283	Contraction	biological_process	spermatogenesis	16	1.34E-03
GO:0007155	Contraction	biological_process	cell adhesion	15	3.44E-03
GO:0006355	Contraction	biological_process	regulation of transcription, DNA-templated	14	5.84E-05
GO:0007586	Contraction	biological_process	digestion	13	1.51E-14
GO:0000278	Contraction	biological_process	mitotic cell cycle	13	2.54E-06
GO:0046872	Contraction	molecular_function	metal ion binding	13	1.29E-05
GO:0007186	Contraction	biological_process	G-protein coupled receptor signaling pathway	13	2.78E-02
GO:0006313	Contraction	biological_process	transposition, DNA-mediated	12	1.76E-10
GO:0030335	Contraction	biological_process	positive regulation of cell migration	11	7.57E-08
GO:0006357	Contraction	biological_process	regulation of transcription from RNA polymerase II promoter	11	3.85E-04
GO:0044826	Contraction	biological_process	viral genome integration into host DNA	11	1.08E-03
GO:0046685	Contraction	biological_process	response to arsenic-containing substance	10	1.20E-14
GO:0016055	Contraction	biological_process	Wnt signaling pathway	10	3.01E-06
GO:0033260	Contraction	biological_process	nuclear DNA replication	9	3.06E-13
GO:0016192	Contraction	biological_process	vesicle-mediated transport	9	2.17E-04
GO:0042273	Contraction	biological_process	ribosomal large subunit biogenesis	9	2.65E-04
GO:0002098	Contraction	biological_process	tRNA wobble uridine modification	9	6.49E-04
GO:0005975	Contraction	biological_process	carbohydrate metabolic process	9	3.69E-03
GO:0004984	Contraction	molecular_function	olfactory receptor activity	9	9.15E-03
GO:0006904	Contraction	biological_process	vesicle docking involved in exocytosis	9	1.16E-02
GO:0046081	Contraction	biological_process	dUTP catabolic process	8	7.74E-12
GO:0038123	Contraction	biological_process	toll-like receptor TLR1:TLR2 signaling pathway	8	7.74E-12
GO:0051786	Contraction	molecular_function	all-trans-retinol 13,14-reductase activity	8	6.69E-11
GO:0006865	Contraction	biological_process	amino acid transport	8	1.31E-07
GO:0031175	Contraction	biological_process	neuron projection development	8	3.50E-03
GO:0019432	Contraction	biological_process	triglyceride biosynthetic process	8	3.50E-03

GO:0044319	Contraction	biological_process	wound healing, spreading of cells	8	4.18E-03
GO:0032496	Contraction	biological_process	response to lipopolysaccharide	8	2.43E-02
GO:0006915	Contraction	biological_process	apoptotic process	8	2.80E-02
GO:0006068	Contraction	biological_process	ethanol catabolic process	7	1.94E-10
GO:0007157	Contraction	biological_process	heterophilic cell-cell adhesion via plasma membrane cell adhesion molecules	7	1.94E-10
GO:0051492	Contraction	biological_process	regulation of stress fiber assembly	7	1.54E-06
GO:0051289	Contraction	biological_process	protein homotetramerization	7	2.67E-03
GO:0043065	Contraction	biological_process	positive regulation of apoptotic process	6	2.44E-02
GO:0070374	Contraction	biological_process	positive regulation of ERK1 and ERK2 cascade	6	2.60E-02
GO:0007601	Contraction	biological_process	visual perception	5	8.60E-09
GO:0003334	Contraction	biological_process	keratinocyte development	5	1.20E-07
GO:0016592	Contraction	cellular_component	mediator complex	5	1.20E-07
GO:0071333	Contraction	biological_process	cellular response to glucose stimulus	5	1.20E-07
GO:0035282	Contraction	biological_process	segmentation	5	2.31E-06
GO:0046883	Contraction	biological_process	regulation of hormone secretion	5	2.45E-05
GO:0035751	Contraction	biological_process	regulation of lysosomal lumen pH	5	2.45E-05
GO:0006776	Contraction	biological_process	vitamin A metabolic process	5	2.45E-05
GO:0043457	Contraction	biological_process	regulation of cellular respiration	5	2.45E-05
GO:0021978	Contraction	biological_process	telencephalon regionalization	5	2.45E-05
GO:0017188	Contraction	molecular_function	aspartate N-acetyltransferase activity	5	2.45E-05
GO:0030208	Contraction	biological_process	dermatan sulfate biosynthetic process	5	2.45E-05
GO:0030672	Contraction	cellular_component	synaptic vesicle membrane	5	2.45E-05
GO:0016567	Contraction	biological_process	protein ubiquitination	5	4.40E-05
GO:0071805	Contraction	biological_process	potassium ion transmembrane transport	5	3.31E-04
GO:0050871	Contraction	biological_process	positive regulation of B cell activation	5	4.50E-04
GO:0043523		biological_process	regulation of neuron apoptotic process	5	1.25E-03

GO:0043149		biological_process	stress fiber assembly	5	1.91E-03
GO:0009791		biological_process	post-embryonic development	5	1.91E-03
GO:0006364		biological_process	rRNA processing	5	2.04E-03
GO:0031514		cellular_component	motile cilium	5	3.30E-03
GO:0046415		biological_process	urate metabolic process	5	1.12E-02
GO:0035567		biological_process	non-canonical Wnt signaling pathway	5	1.68E-02
GO:0016338		biological_process	calcium-independent cell-cell adhesion via plasma membrane cell-adhesion molecules	5	2.01E-02
GO:0007219		biological_process	Notch signaling pathway	5	2.01E-02
GO:0045663		biological_process	positive regulation of myoblast differentiation	4	2.94E-06
GO:0035220		biological_process	wing disc development	4	2.94E-06
GO:0045944		biological_process	positive regulation of transcription from RNA polymerase II promoter	4	1.47E-05
GO:0061630		molecular_function	ubiquitin protein ligase activity	4	3.01E-04
GO:0006556		biological_process	S-adenosylmethionine biosynthetic process	4	3.01E-04
GO:2001256		biological_process	regulation of store-operated calcium entry	4	1.94E-03
GO:2000582		biological_process	positive regulation of ATP-dependent microtubule motor activity, plus-end-directed	4	1.94E-03
GO:0039702		biological_process	viral budding via host ESCRT complex	4	1.55E-02
GO:0005096		molecular_function	GTPase activator activity	4	2.46E-02
GO:0010867		biological_process	positive regulation of triglyceride biosynthetic process	3	3.25E-03
GO:0048168		biological_process	regulation of neuronal synaptic plasticity	3	4.68E-03
GO:0045599		biological_process	negative regulation of fat cell differentiation	3	8.46E-03
GO:0005044		molecular_function	scavenger receptor activity	3	1.64E-02
GO:0007017		biological_process	microtubule-based process	3	3.12E-02
GO:0005886		cellular_component	plasma membrane	2	7.77E-04
GO:0010092		biological_process	specification of organ identity	2	1.74E-03
GO:0007399		biological_process	nervous system development	2	1.26E-02

Supplementary Table 21. Rapidly losses gene families between Magellanic and African penguins

**Magellanic-African**					
**GO ID**	**Event**	**Namespace**	**Name (Child term)**	**Count p-value**	**p-value**
GO:0007608	Contraction	biological_process	sensory perception of smell	28	1.01E-11
GO:0000278	Contraction	biological_process	mitotic cell cycle	21	2.08E-16
GO:0016020	Contraction	cellular_component	membrane	21	1.20E-12
GO:0015031	Contraction	biological_process	protein transport	10	7.78E-03
GO:0007165	Contraction	biological_process	signal transduction	6	5.72E-18
GO:0006355	Contraction	biological_process	regulation of transcription, DNA-templated	6	2.07E-06
GO:0006953	Contraction	biological_process	acute-phase response	5	3.50E-04
GO:0016021	Contraction	cellular_component	integral component of membrane	4	2.42E-19
GO:0007283	Contraction	biological_process	spermatogenesis	4	4.26E-07
GO:0016192	Contraction	biological_process	vesicle-mediated transport	4	4.25E-05
GO:0007016	Contraction	biological_process	cytoskeletal anchoring at plasma membrane	4	6.80E-04
GO:0060545	Contraction	biological_process	positive regulation of necroptotic process	3	3.02E-05
GO:1901031	Contraction	biological_process	regulation of response to reactive oxygen species	3	3.02E-05
GO:0006412	Contraction	biological_process	translation	3	1.20E-04
GO:0035744	Contraction	biological_process	T-helper 1 cell cytokine production	3	1.45E-03
GO:0002026	Contraction	biological_process	regulation of the force of heart contraction	3	1.45E-03

GO:0004129	Contraction	molecular_function	cytochrome-c oxidase activity	3	5.03E-03
GO:0005044	Contraction	molecular_function	scavenger receptor activity	3	7.81E-03
GO:0051258	Contraction	biological_process	protein polymerization	3	9.47E-03
GO:0007017	Contraction	biological_process	microtubule-based process	3	1.55E-02
GO:0046872	Contraction	molecular_function	metal ion binding	2	4.98E-10
GO:0016567	Contraction	biological_process	protein ubiquitination	2	2.52E-05
GO:0004715	Contraction	molecular_function	non-membrane spanning protein tyrosine kinase activity	2	9.79E-04
GO:0032648	Contraction	biological_process	regulation of interferon-beta production	2	9.79E-04
GO:0050793	Contraction	biological_process	regulation of developmental process	2	9.79E-04
GO:0060179	Contraction	biological_process	male mating behavior	2	9.79E-04
GO:0048037	Contraction	molecular_function	cofactor binding	2	9.79E-04
GO:0031998	Contraction	biological_process	regulation of fatty acid beta-oxidation	2	9.79E-04
GO:0006105	Contraction	biological_process	succinate metabolic process	2	9.79E-04
GO:0016616	Contraction	molecular_function	oxidoreductase activity, acting on the CH-OH group of donors, NAD or NADP as acceptor	2	2.85E-03
GO:0060994	Contraction	biological_process	regulation of transcription from RNA polymerase II promoter involved in kidney development	2	2.85E-03
GO:0033993	Contraction	biological_process	response to lipid	2	8.91E-03
GO:1900746	Contraction	biological_process	regulation of vascular endothelial growth factor signaling pathway	2	8.91E-03
GO:0007275	Contraction	biological_process	multicellular organismal development	2	1.20E-02
GO:0032438	Contraction	biological_process	melanosome organization	2	1.30E-02
GO:0042060	Contraction	biological_process	wound healing	2	1.39E-02
GO:0030593	Contraction	biological_process	neutrophil chemotaxis	2	1.76E-02

Supplementary Table 22. Rapidly losses gene families in Galápagos penguin

Galápagos					
**GO ID**	**Event**	**Namespace**	**Name (Child term)**	**Count p-value**	**p-value**
GO:0006334	Contraction	biological_process	nucleosome assembly	87	4.15E-69
GO:0005200	Contraction	molecular_function	structural constituent of cytoskeleton	87	2.32E-35
GO:0007165	Contraction	biological_process	signal transduction	46	5.81E-27
GO:0006355	Contraction	biological_process	regulation of transcription, DNA-templated	40	8.17E-06
GO:0007608	Contraction	biological_process	sensory perception of smell	37	1.94E-05
GO:0006325	Contraction	biological_process	chromatin organization	27	1.29E-09
GO:0007156	Contraction	biological_process	homophilic cell adhesion via plasma membrane adhesion molecules	25	1.25E-02
GO:0016021	Contraction	cellular_component	integral component of membrane	24	3.63E-42
GO:0046872	Contraction	molecular_function	metal ion binding	24	5.26E-13
GO:0016032	Contraction	biological_process	viral process	24	3.77E-10
GO:0007525	Contraction	biological_process	somatic muscle development	18	7.17E-09
GO:0006511	Contraction	biological_process	ubiquitin-dependent protein catabolic process	17	2.60E-24
GO:0007283	Contraction	biological_process	spermatogenesis	16	2.21E-15
GO:0015031	Contraction	biological_process	protein transport	16	1.96E-11
GO:0007601	Contraction	biological_process	visual perception	15	6.11E-15
GO:0050776	Contraction	biological_process	regulation of immune response	15	2.96E-03
GO:0016055	Contraction	biological_process	Wnt signaling pathway	14	2.26E-17
GO:0036158	Contraction	biological_process	outer dynein arm assembly	14	1.30E-03

GO:0006417	Contraction	biological_process	regulation of translation	14	3.64E-03
GO:0050852	Contraction	biological_process	T cell receptor signaling pathway	14	1.83E-02
GO:0006357	Contraction	biological_process	regulation of transcription from RNA polymerase II promoter	13	1.82E-13
GO:0050661	Contraction	molecular_function	NADP binding	13	2.91E-13
GO:0045893	Contraction	biological_process	positive regulation of transcription, DNA-templated	13	1.57E-03
GO:0000278	Contraction	biological_process	mitotic cell cycle	13	4.48E-03
GO:0007275	Contraction	biological_process	multicellular organismal development	12	5.52E-03
GO:0030335	Contraction	biological_process	positive regulation of cell migration	11	1.34E-04
GO:0042310	Contraction	biological_process	vasoconstriction	11	1.20E-02
GO:0034765	Contraction	biological_process	regulation of ion transmembrane transport	10	2.09E-03
GO:0045944	Contraction	biological_process	positive regulation of transcription from RNA polymerase II promoter	9	1.12E-10
GO:0045110	Contraction	biological_process	intermediate filament bundle assembly	9	3.79E-10
GO:0000921	Contraction	biological_process	septin ring assembly	9	1.75E-04
GO:0051259	Contraction	biological_process	protein oligomerization	9	1.87E-02
GO:0016567	Contraction	biological_process	protein ubiquitination	8	8.38E-12
GO:0046081	Contraction	biological_process	dUTP catabolic process	8	4.27E-09
GO:0038123	Contraction	biological_process	toll-like receptor TLR1:TLR2 signaling pathway	8	4.27E-09
GO:0042060	Contraction	biological_process	wound healing	8	2.84E-04
GO:0060236	Contraction	biological_process	regulation of mitotic spindle organization	8	6.86E-03
GO:0035567	Contraction	biological_process	non-canonical Wnt signaling pathway	8	1.45E-02
GO:0007140	Contraction	biological_process	male meiosis	7	4.80E-08
GO:0031957	Contraction	molecular_function	very long-chain fatty acid-CoA ligase activity	7	1.09E-05
GO:0045892	Contraction	biological_process	negative regulation of transcription, DNA-templated	7	8.09E-04

GO:0006412	Contraction	biological_process	translation	6	1.12E-13
GO:0008270	Contraction	molecular_function	zinc ion binding	6	3.65E-08
GO:0005634	Contraction	cellular_componen t	nucleus	6	2.74E-06
GO:0070723	Contraction	biological_process	response to cholesterol	6	3.43E-06
GO:0030071	Contraction	biological_process	regulation of mitotic metaphase/anaphase transition	6	1.56E-04
GO:0000122	Contraction	biological_process	negative regulation of transcription from RNA polymerase II promoter	6	2.57E-04
GO:0007186	Contraction	biological_process	G-protein coupled receptor signaling pathway	6	5.84E-04
GO:0001570	Contraction	biological_process	vasculogenesis	6	1.49E-02
GO:0052689	Contraction	molecular_function	carboxylic ester hydrolase activity	6	1.60E-02
GO:0007188	Contraction	biological_process	adenylate cyclase-modulating G-protein coupled receptor signaling pathway	5	6.01E-06
GO:0045657	Contraction	biological_process	positive regulation of monocyte differentiation	5	6.01E-06
GO:2000036	Contraction	biological_process	regulation of stem cell maintenance	5	6.01E-06
GO:0071333	Contraction	biological_process	cellular response to glucose stimulus	5	6.01E-06
GO:0030850	Contraction	biological_process	prostate gland development	5	6.01E-06
GO:0051260	Contraction	biological_process	protein homooligomerization	5	8.00E-04
GO:0006937	Contraction	biological_process	regulation of muscle contraction	5	9.47E-04
GO:0043457	Contraction	biological_process	regulation of cellular respiration	5	9.47E-04
GO:0021978	Contraction	biological_process	telencephalon regionalization	5	9.47E-04
GO:0017188	Contraction	molecular_function	aspartate N-acetyltransferase activity	5	9.47E-04
GO:0051493	Contraction	biological_process	regulation of cytoskeleton organization	5	9.47E-04
GO:0060510	Contraction	biological_process	Type II pneumocyte differentiation	5	9.47E-04
GO:0045862	Contraction	biological_process	positive regulation of proteolysis	5	9.47E-04
GO:0009651	Contraction	biological_process	response to salt stress	5	9.47E-04

GO:0033209	Contraction	biological_process	tumor necrosis factor-mediated signaling pathway	5	1.11E-02
GO:0016192	Contraction	biological_process	vesicle-mediated transport	4	1.45E-19
GO:0006351	Contraction	biological_process	transcription, DNA-templated	4	3.52E-07
GO:0045663	Contraction	biological_process	positive regulation of myoblast differentiation	4	6.69E-05
GO:0006434	Contraction	biological_process	seryl-tRNA aminoacylation	4	6.69E-05
GO:0045766	Contraction	biological_process	positive regulation of angiogenesis	4	6.69E-05
GO:0002246	Contraction	biological_process	wound healing involved in inflammatory response	4	6.69E-05
GO:0061563	Contraction	biological_process	trigeminal ganglion structural organization	4	6.69E-05
GO:0006468	Contraction	biological_process	protein phosphorylation	4	1.98E-04
GO:0001975	Contraction	biological_process	response to amphetamine	4	3.21E-03
GO:0061630	Contraction	molecular_function	ubiquitin protein ligase activity	4	5.26E-03
GO:0007214	Contraction	biological_process	gamma-aminobutyric acid signaling pathway	4	5.26E-03
GO:0050690	Contraction	biological_process	regulation of defense response to virus by virus	4	5.26E-03
GO:0008380	Contraction	biological_process	RNA splicing	3	9.01E-14
GO:0006508	Contraction	biological_process	proteolysis	3	6.48E-13
GO:0007264	Contraction	biological_process	small GTPase mediated signal transduction	3	1.24E-08
GO:0055085	Contraction	biological_process	transmembrane transport	3	1.19E-07
GO:0007169	Contraction	biological_process	transmembrane receptor protein tyrosine kinase signaling pathway	3	2.78E-06
GO:0006979	Contraction	biological_process	response to oxidative stress	3	4.27E-06
GO:0006367	Contraction	biological_process	transcription initiation from RNA polymerase II promoter	3	5.06E-06
GO:0006477	Contraction	biological_process	protein sulfation	3	7.43E-04
GO:0030201	Contraction	biological_process	heparan sulfate proteoglycan metabolic process	3	7.43E-04
GO:0042942	Contraction	biological_process	D-serine transport	3	7.43E-04

GO:0072513	Contraction	biological_process	positive regulation of secondary heart field cardioblast proliferation	3	7.43E-04
GO:0030687	Contraction	cellular_component	preribosome, large subunit precursor	3	7.43E-04
GO:2000045	Contraction	biological_process	regulation of G1/S transition of mitotic cell cycle	3	7.43E-04
GO:0060223	Contraction	biological_process	retinal rod cell fate commitment	3	7.43E-04
GO:0003420	Contraction	biological_process	regulation of growth plate cartilage chondrocyte proliferation	3	7.43E-04
GO:0072321	Contraction	biological_process	chaperone-mediated protein transport	3	7.43E-04
GO:0043204	Contraction	cellular_component	perikaryon	3	7.43E-04
GO:0030195	Contraction	biological_process	negative regulation of blood coagulation	3	7.43E-04
GO:0001164	Contraction	molecular_function	RNA polymerase I CORE element sequence-specific DNA binding	3	7.43E-04
GO:0060535	Contraction	biological_process	trachea cartilage morphogenesis	3	7.43E-04
GO:0042823	Contraction	biological_process	pyridoxal phosphate biosynthetic process	3	7.43E-04
GO:0045725	Contraction	biological_process	positive regulation of glycogen biosynthetic process	3	7.43E-04
GO:0008154	Contraction	biological_process	actin polymerization or depolymerization	3	7.43E-04
GO:0021722	Contraction	biological_process	superior olivary nucleus maturation	3	7.43E-04
GO:0071731	Contraction	biological_process	response to nitric oxide	3	7.43E-04
GO:0070863	Contraction	biological_process	positive regulation of protein exit from endoplasmic reticulum	3	7.43E-04
GO:0005355	Contraction	molecular_function	glucose transmembrane transporter activity	3	7.43E-04
GO:0050772	Contraction	biological_process	positive regulation of axonogenesis	3	7.43E-04
GO:0010610	Contraction	biological_process	regulation of mRNA stability involved in response to stress	3	2.70E-03
GO:0016188	Contraction	biological_process	synaptic vesicle maturation	3	6.15E-03

GO:0061569	Contraction	biological_process	UDP phosphorylation	3	6.15E-03
GO:0060285	Contraction	biological_process	cilium-dependent cell motility	3	6.15E-03
GO:0015887	Contraction	biological_process	pantothenate transmembrane transport	3	6.15E-03
GO:0042136	Contraction	biological_process	neurotransmitter biosynthetic process	3	6.15E-03
GO:0008284	Contraction	biological_process	positive regulation of cell proliferation	3	6.90E-03
GO:0006368	Contraction	biological_process	transcription elongation from RNA polymerase II promoter	3	7.92E-03
GO:1901998	Contraction	biological_process	toxin transport	3	9.08E-03
GO:0006915	Contraction	biological_process	apoptotic process	3	1.45E-02
GO:0032496	Contraction	biological_process	response to lipopolysaccharide	3	1.76E-02
GO:1990440	Contraction	biological_process	positive regulation of transcription from RNA polymerase II promoter in response to endoplasmic reticulum stress	3	1.78E-02
GO:0007416	Contraction	biological_process	synapse assembly	2	3.32E-03
GO:0006694	Contraction	biological_process	steroid biosynthetic process	2	4.88E-03
GO:0001501	Contraction	biological_process	skeletal system development	2	6.60E-03
GO:0097191	Contraction	biological_process	extrinsic apoptotic signaling pathway	2	8.23E-03
GO:0050793	Contraction	biological_process	regulation of developmental process	2	8.23E-03
GO:0060179	Contraction	biological_process	male mating behavior	2	8.23E-03
GO:0031998	Contraction	biological_process	regulation of fatty acid beta-oxidation	2	8.23E-03
GO:0006105	Contraction	biological_process	succinate metabolic process	2	8.23E-03
GO:0016322	Contraction	biological_process	neuron remodeling	2	8.23E-03
GO:0006396	Contraction	biological_process	RNA processing	2	1.97E-02

Supplementary Table 23. Rapidly evolving gene families in Humboldt penguin

**Humboldt**					
**GO ID**	**Event**	**Namespace**	**Name (Child term)**	**Count p-value**	**p-value**
GO:0007165	Contraction	biological_process	signal transduction	38	1.17E-09
GO:0016021	Contraction	cellular_component	integral component of membrane	33	4.24E-11
GO:0006511	Contraction	biological_process	ubiquitin-dependent protein catabolic process	32	1.04E-03
GO:0046872	Contraction	molecular_function	metal ion binding	29	8.66E-03
GO:0044826	Contraction	biological_process	viral genome integration into host DNA	28	4.11E-15
GO:0006355	Contraction	biological_process	regulation of transcription, DNA-templated	21	1.32E-04
GO:0051453	Contraction	biological_process	regulation of intracellular pH	17	1.68E-16
GO:0002250	Contraction	biological_process	adaptive immune response	16	2.92E-14
GO:0007155	Contraction	biological_process	cell adhesion	15	1.94E-02
GO:0007283	Contraction	biological_process	spermatogenesis	14	1.54E-06
GO:0006313	Contraction	biological_process	transposition, DNA-mediated	13	1.50E-10
GO:0007018	Contraction	biological_process	microtubule-based movement	13	1.18E-03
GO:0061408	Contraction	biological_process	positive regulation of transcription from RNA polymerase II promoter in response to heat stress	10	4.41E-07
GO:0006885	Contraction	biological_process	regulation of pH	9	3.87E-09
GO:0051607	Contraction	biological_process	defense response to virus	9	2.24E-06
GO:0051932	Contraction	biological_process	synaptic transmission, GABAergic	9	9.51E-03
GO:0016055	Contraction	biological_process	Wnt signaling pathway	8	1.97E-10
GO:0010447	Contraction	biological_process	response to acidic pH	8	4.93E-08
GO:0002009	Contraction	biological_process	morphogenesis of an epithelium	8	3.36E-06
GO:0009791	Contraction	biological_process	post-embryonic development	8	1.12E-05
GO:0039702	Contraction	biological_process	viral budding via host ESCRT complex	8	2.23E-05

GO:0051603	Contraction	biological_process	proteolysis involved in cellular protein catabolic process	8	7.77E-03
GO:0051492	Contraction	biological_process	regulation of stress fiber assembly	7	7.03E-06
GO:0006366	Contraction	biological_process	transcription from RNA polymerase II promoter	7	1.01E-03
GO:0048167	Contraction	biological_process	regulation of synaptic plasticity	7	1.14E-02
GO:0007034	Contraction	biological_process	vacuolar transport	6	8.01E-04
GO:0034765	Contraction	biological_process	regulation of ion transmembrane transport	6	1.96E-02
GO:0033993	Contraction	biological_process	response to lipid	5	3.78E-07
GO:0016592	Contraction	cellular_component	mediator complex	5	3.78E-07
GO:0048660	Contraction	biological_process	regulation of smooth muscle cell proliferation	5	3.78E-07
GO:0045944	Contraction	biological_process	positive regulation of transcription from RNA polymerase II promoter	5	1.02E-06
GO:0035282	Contraction	biological_process	segmentation	5	7.14E-06
GO:0030208	Contraction	biological_process	dermatan sulfate biosynthetic process	5	7.32E-05
GO:0006364	Contraction	biological_process	rRNA processing	5	1.22E-04
GO:0005978	Contraction	biological_process	glycogen biosynthetic process	5	6.71E-04
GO:0016973	Contraction	biological_process	poly(A)+ mRNA export from nucleus	5	1.63E-03
GO:0008234	Contraction	molecular_function	cysteine-type peptidase activity	5	2.66E-03
GO:0043149	Contraction	biological_process	stress fiber assembly	5	4.93E-03
GO:0042060	Contraction	biological_process	wound healing	5	8.09E-03
GO:0031514	Contraction	cellular_component	motile cilium	5	8.23E-03
GO:0002040	Contraction	biological_process	sprouting angiogenesis	5	8.23E-03
GO:0021762	Contraction	biological_process	substantia nigra development	5	2.05E-02
GO:0005509	Contraction	molecular_function	calcium ion binding	5	2.47E-02
GO:0006556	Contraction	biological_process	S-adenosylmethionine biosynthetic process	4	7.11E-04
GO:0007016	Contraction	biological_process	cytoskeletal anchoring at plasma membrane	4	4.34E-03

GO:0060314	Contraction	biological_process	regulation of ryanodine-sensitive calcium-release channel activity	4	4.34E-03
GO:0007169	Contraction	biological_process	transmembrane receptor protein tyrosine kinase signaling pathway	4	9.06E-03
GO:0007601	Contraction	biological_process	visual perception	3	6.95E-14
GO:0006357	Contraction	biological_process	regulation of transcription from RNA polymerase II promoter	3	6.04E-12
GO:0016192	Contraction	biological_process	vesicle-mediated transport	3	8.38E-11
GO:0006412	Contraction	biological_process	translation	3	1.19E-08
GO:0008380	Contraction	biological_process	RNA splicing	3	4.52E-07
GO:0008270	Contraction	molecular_function	zinc ion binding	3	1.83E-05
GO:0003723	Contraction	molecular_function	RNA binding	3	2.12E-05
GO:0045893	Contraction	biological_process	positive regulation of transcription, DNA-templated	3	1.13E-04
GO:1902775	Contraction	biological_process	mitochondrial large ribosomal subunit assembly	3	1.42E-04
GO:0060541	Contraction	biological_process	respiratory system development	3	1.42E-04
GO:0003431	Contraction	biological_process	growth plate cartilage chondrocyte development	3	1.42E-04
GO:1901082	Contraction	biological_process	positive regulation of relaxation of smooth muscle	3	1.42E-04
GO:0046889	Contraction	biological_process	positive regulation of lipid biosynthetic process	3	1.42E-04
GO:0070508	Contraction	biological_process	cholesterol import	3	1.42E-04
GO:0010765	Contraction	biological_process	positive regulation of sodium ion transport	3	1.42E-04
GO:0006351	Contraction	biological_process	transcription, DNA-templated	3	4.33E-04
GO:0035745	Contraction	biological_process	T-helper 2 cell cytokine production	3	5.40E-04
GO:0046549	Contraction	biological_process	retinal cone cell development	3	1.28E-03
GO:0046328	Contraction	biological_process	regulation of JNK cascade	3	1.28E-03
GO:1903861	Contraction	biological_process	positive regulation of dendrite extension	3	2.43E-03
GO:0006979	Contraction	biological_process	response to oxidative stress	3	3.79E-03

GO:0007186	Contraction	biological_process	G-protein coupled receptor signaling pathway	3	5.47E-03
GO:0010867	Contraction	biological_process	positive regulation of triglyceride biosynthetic process	3	6.11E-03
GO:0034497	Contraction	biological_process	protein localization to pre-autophagosomal structure	3	6.11E-03
GO:1901329	Contraction	biological_process	regulation of odontoblast differentiation	3	6.11E-03
GO:0006409	Contraction	biological_process	tRNA export from nucleus	3	6.11E-03
GO:0030433	Contraction	biological_process	ER-associated ubiquitin-dependent protein catabolic process	3	7.54E-03
GO:0051260	Contraction	biological_process	protein homooligomerization	3	1.25E-02
GO:0004129	Contraction	molecular_function	cytochrome-c oxidase activity	3	1.94E-02
GO:0009268	Contraction	biological_process	response to pH	3	1.94E-02
GO:0036295	Contraction	biological_process	cellular response to increased oxygen levels	3	2.39E-02
GO:0043393	Contraction	biological_process	regulation of protein binding	3	2.39E-02
GO:0015031	Contraction	biological_process	protein transport	2	5.20E-13
GO:0005576	Contraction	cellular_component	extracellular region	2	1.44E-04
GO:0007608	Contraction	biological_process	sensory perception of smell	2	7.05E-04
GO:1903818	Contraction	biological_process	positive regulation of voltage-gated potassium channel activity	2	2.74E-03
GO:0015826	Contraction	biological_process	threonine transport	2	2.74E-03
GO:0032648	Contraction	biological_process	regulation of interferon-beta production	2	2.74E-03
GO:0048037	Contraction	molecular_function	cofactor binding	2	2.74E-03
GO:0060976	Contraction	biological_process	coronary vasculature development	2	2.74E-03
GO:0016616	Contraction	molecular_function	oxidoreductase activity, acting on the CH-OH group of donors, NAD or NADP as acceptor	2	7.79E-03
GO:0003823	Contraction	molecular_function	antigen binding	2	2.33E-02
GO:1900746	Contraction	biological_process	regulation of vascular endothelial growth factor signaling pathway	2	2.33E-02

Supplementary Table 24. Rapidly losses gene families in Magellanic penguin.

Magellanic					
**GO ID**	**Event**	**Namespace**	**Name (Child term)**	**Count p-value**	**p-value**
GO:0006334	Expansion	biological_process	nucleosome assembly	92	2.38E-94
GO:0005200	Expansion	molecular_function	structural constituent of cytoskeleton	82	3.81E-44
GO:0006355	Expansion	biological_process	regulation of transcription, DNA-templated	57	1.96E-02
GO:0006511	Expansion	biological_process	ubiquitin-dependent protein catabolic process	46	8.20E-03
GO:0006325	Expansion	biological_process	chromatin organization	34	3.95E-20
GO:0016032	Expansion	biological_process	viral process	29	2.51E-03
GO:0016055	Expansion	biological_process	Wnt signaling pathway	27	3.53E-04
GO:0003774	Expansion	molecular_function	motor activity	25	5.96E-08
GO:0015031	Expansion	biological_process	protein transport	25	4.34E-03
GO:0050776	Expansion	biological_process	regulation of immune response	22	1.50E-09
GO:0000122	Expansion	biological_process	negative regulation of transcription from RNA polymerase II promoter	22	4.70E-03
GO:0061038	Expansion	biological_process	uterus morphogenesis	21	2.41E-20
GO:0006890	Expansion	biological_process	retrograde vesicle-mediated transport, Golgi to ER	21	3.19E-04
GO:0006412	Expansion	biological_process	translation	21	1.97E-02
GO:0046872	Expansion	molecular_function	metal ion binding	20	8.38E-08
GO:0030433	Expansion	biological_process	ER-associated ubiquitin-dependent protein catabolic process	20	6.09E-03
GO:0007283	Expansion	biological_process	spermatogenesis	19	2.52E-02
GO:0007525	Expansion	biological_process	somatic muscle development	18	2.05E-11
GO:0060412	Expansion	biological_process	ventricular septum morphogenesis	18	6.82E-07
GO:2000300	Expansion	biological_process	regulation of synaptic vesicle exocytosis	16	2.12E-06
GO:0006357	Expansion	biological_process	regulation of transcription from RNA polymerase II promoter	15	2.20E-06

GO:0002244	Expansion	biological_process	hematopoietic progenitor cell differentiation	14	7.75E-09
GO:0048741	Expansion	biological_process	skeletal muscle fiber development	14	2.19E-07
GO:0008380	Expansion	biological_process	RNA splicing	14	4.01E-03
GO:0007165	Expansion	biological_process	signal transduction	13	5.13E-35
GO:0003677	Expansion	molecular_function	DNA binding	13	1.08E-03
GO:0006904	Expansion	biological_process	vesicle docking involved in exocytosis	13	4.46E-03
GO:0004111	Expansion	molecular_function	creatine kinase activity	12	3.37E-15
GO:0010142	Expansion	biological_process	farnesyl diphosphate biosynthetic process, mevalonate pathway	12	3.37E-15
GO:0004849	Expansion	molecular_function	uridine kinase activity	12	3.37E-15
GO:0016567	Expansion	biological_process	protein ubiquitination	12	6.74E-05
GO:0030100	Expansion	biological_process	regulation of endocytosis	12	8.29E-05
GO:0007601	Expansion	biological_process	visual perception	11	2.15E-10
GO:0030335	Expansion	biological_process	positive regulation of cell migration	11	5.12E-06
GO:0006366	Expansion	biological_process	transcription from RNA polymerase II promoter	11	2.60E-03
GO:0007015	Expansion	biological_process	actin filament organization	10	1.99E-09
GO:0051321	Expansion	biological_process	meiotic cell cycle	10	1.07E-05
GO:0061097	Expansion	biological_process	regulation of protein tyrosine kinase activity	9	1.48E-11
GO:0016192	Expansion	biological_process	vesicle-mediated transport	9	1.31E-08
GO:0045944	Expansion	biological_process	positive regulation of transcription from RNA polymerase II promoter	9	4.04E-06
GO:0051607	Expansion	biological_process	defense response to virus	9	1.10E-05
GO:0038123	Expansion	biological_process	toll-like receptor TLR1:TLR2 signaling pathway	8	2.41E-10
GO:0007605	Expansion	biological_process	sensory perception of sound	8	5.08E-03
GO:0005634	Expansion	cellular_component	nucleus	8	5.49E-03
GO:0050852	Expansion	biological_process	T cell receptor signaling pathway	8	1.41E-02
GO:0006897	Expansion	biological_process	endocytosis	8	1.45E-02
GO:0016021	Expansion	cellular_component	integral component of membrane	7	2.85E-40
GO:0042450	Expansion	biological_process	arginine biosynthetic process via ornithine	7	3.90E-09

GO:0034632	Expansion	molecular_function	retinol transporter activity	7	3.90E-09
GO:0060819	Expansion	biological_process	inactivation of X chromosome by genetic imprinting	7	3.90E-09
GO:0007140	Expansion	biological_process	male meiosis	7	3.90E-09
GO:0035498	Expansion	biological_process	carnosine metabolic process	7	3.90E-09
GO:0007276	Expansion	biological_process	gamete generation	7	3.90E-09
GO:0010818	Expansion	biological_process	T cell chemotaxis	7	1.24E-07
GO:0035360	Expansion	biological_process	positive regulation of peroxisome proliferator activated receptor signaling pathway	7	2.24E-06
GO:0006777	Expansion	biological_process	Mo-molybdopterin cofactor biosynthetic process	7	2.24E-06
GO:0060261	Expansion	biological_process	positive regulation of transcription initiation from RNA polymerase II promoter	7	2.24E-06
GO:0045773	Expansion	biological_process	positive regulation of axon extension	7	2.24E-06
GO:0070904	Expansion	biological_process	transepithelial L-ascorbic acid transport	7	1.51E-05
GO:0048845	Expansion	biological_process	venous blood vessel morphogenesis	7	4.00E-05
GO:0055012	Expansion	biological_process	ventricular cardiac muscle cell differentiation	7	4.00E-05
GO:0051382	Expansion	biological_process	kinetochore assembly	7	4.00E-05
GO:0004386	Expansion	molecular_function	helicase activity	7	6.15E-05
GO:0006471	Expansion	biological_process	protein ADP-ribosylation	7	6.15E-05
GO:0019731	Expansion	biological_process	antibacterial humoral response	7	1.32E-04
GO:0002009	Expansion	biological_process	morphogenesis of an epithelium	7	1.32E-04
GO:0007034	Expansion	biological_process	vacuolar transport	7	3.44E-04
GO:0071277	Expansion	biological_process	cellular response to calcium ion	7	7.61E-04
GO:0097428	Expansion	biological_process	protein maturation by iron-sulfur cluster transfer	7	2.65E-03
GO:1901796	Expansion	biological_process	regulation of signal transduction by p53 class mediator	7	3.72E-03
GO:0018105	Expansion	biological_process	peptidyl-serine phosphorylation	7	4.36E-03
GO:0001659	Expansion	biological_process	temperature homeostasis	7	5.87E-03
GO:0043627	Expansion	biological_process	response to estrogen	7	7.71E-03
GO:0001944	Expansion	biological_process	vasculature development	7	7.71E-03

GO:0006479	Expansion	biological_process	protein methylation	7	8.76E-03
GO:0019058	Expansion	biological_process	viral life cycle	7	1.54E-02
GO:0009408	Expansion	biological_process	response to heat	7	1.88E-02
GO:0045892	Expansion	biological_process	negative regulation of transcription, DNA-templated	7	2.50E-02
GO:0016485	Expansion	biological_process	protein processing	7	2.67E-02
GO:0060078	Expansion	biological_process	regulation of postsynaptic membrane potential	6	6.28E-08
GO:0005886	Expansion	cellular_component	plasma membrane	6	1.23E-03
GO:0052689	Expansion	molecular_function	carboxylic ester hydrolase activity	6	3.28E-03
GO:0043565	Expansion	molecular_function	sequence-specific DNA binding	6	1.58E-02
GO:0003016	Expansion	biological_process	respiratory system process	5	1.01E-06
GO:0045429	Expansion	biological_process	positive regulation of nitric oxide biosynthetic process	5	1.01E-06
GO:0007188	Expansion	biological_process	adenylate cyclase-modulating G-protein coupled receptor signaling pathway	5	1.01E-06
GO:0010863	Expansion	biological_process	positive regulation of phospholipase C activity	5	1.01E-06
GO:0060158	Expansion	biological_process	phospholipase C-activating dopamine receptor signaling pathway	5	1.01E-06
GO:0010971	Expansion	biological_process	positive regulation of G2/M transition of mitotic cell cycle	5	1.01E-06
GO:0008270	Expansion	molecular_function	zinc ion binding	5	1.71E-05
GO:0046034	Expansion	biological_process	ATP metabolic process	5	9.81E-05
GO:0006937	Expansion	biological_process	regulation of muscle contraction	5	1.84E-04
GO:0021978	Expansion	biological_process	telencephalon regionalization	5	1.84E-04
GO:0060510	Expansion	biological_process	Type II pneumocyte differentiation	5	1.84E-04
GO:2000582	Expansion	biological_process	positive regulation of ATP-dependent microtubule motor activity, plus-end-directed	5	1.13E-03
GO:0001504	Expansion	biological_process	neurotransmitter uptake	5	1.13E-03
GO:1903358	Expansion	biological_process	regulation of Golgi organization	5	1.59E-03
GO:0071805	Expansion	biological_process	potassium ion transmembrane transport	5	2.16E-03
GO:0045202	Expansion	cellular_component	synapse	5	4.74E-03
GO:0046982	Expansion	molecular_function	protein heterodimerization activity	5	7.30E-03

GO:0006936	Expansion	biological_process	muscle contraction	5	1.06E-02
GO:0031098	Expansion	biological_process	stress-activated protein kinase signaling cascade	5	1.20E-02
GO:0032434	Expansion	biological_process	regulation of proteasomal ubiquitin-dependent protein catabolic process	5	1.25E-02
GO:0016239	Expansion	biological_process	positive regulation of macroautophagy	5	1.47E-02
GO:0040020	Expansion	biological_process	regulation of meiotic nuclear division	4	1.61E-05
GO:0006434	Expansion	biological_process	seryl-tRNA aminoacylation	4	1.61E-05
GO:0060048	Expansion	biological_process	cardiac muscle contraction	4	1.61E-05
GO:0061563	Expansion	biological_process	trigeminal ganglion structural organization	4	1.61E-05
GO:0019511	Expansion	biological_process	peptidyl-proline hydroxylation	4	1.61E-05
GO:0060322	Expansion	biological_process	head development	4	1.61E-05
GO:0001975	Expansion	biological_process	response to amphetamine	4	8.69E-04
GO:0007214	Expansion	biological_process	gamma-aminobutyric acid signaling pathway	4	1.47E-03
GO:0007522	Expansion	biological_process	visceral muscle development	4	1.47E-03
GO:0031957	Expansion	molecular_function	very long-chain fatty acid-CoA ligase activity	4	3.37E-03
GO:0007156	Expansion	biological_process	homophilic cell adhesion via plasma membrane adhesion molecules	4	4.10E-03
GO:0007399	Expansion	biological_process	nervous system development	4	5.67E-03
GO:0008217	Expansion	biological_process	regulation of blood pressure	4	8.42E-03
GO:0051496	Expansion	biological_process	positive regulation of stress fiber assembly	4	8.42E-03
GO:0048515	Expansion	biological_process	spermatid differentiation	4	8.42E-03
GO:0061032	Expansion	biological_process	visceral serous pericardium development	4	1.08E-02
GO:0016226	Expansion	biological_process	iron-sulfur cluster assembly	4	2.36E-02
GO:0030278	Expansion	biological_process	regulation of ossification	3	2.56E-04
GO:0006468	Expansion	biological_process	protein phosphorylation	3	2.59E-03
GO:0014029	Expansion	biological_process	neural crest formation	3	1.03E-02
GO:0061551	Expansion	biological_process	trigeminal ganglion development	3	1.03E-02
GO:0048168	Expansion	biological_process	regulation of neuronal synaptic plasticity	3	1.45E-02
GO:0007179	Expansion	biological_process	transforming growth factor beta receptor signaling pathway	2	7.73E-05

GO:0048384	Expansion	biological_process	retinoic acid receptor signaling pathway	2	4.04E-03
GO:0072560	Expansion	biological_process	type B pancreatic cell maturation	2	4.04E-03
GO:0007603	Expansion	biological_process	phototransduction, visible light	2	4.04E-03
GO:0010762	Expansion	biological_process	regulation of fibroblast migration	2	4.04E-03
GO:0048511	Expansion	biological_process	rhythmic process	2	2.28E-02

Supplementary Table 25. Rapidly losses gene families in African penguin.

**African**					
**GO ID**	**Event**	**Namespace**	**Name (Child term)**	**Count p-value**	**p-value**
GO:0006334	Contraction	biological_process	nucleosome assembly	92	2.03E-77
GO:0007165	Contraction	biological_process	signal transduction	54	6.99E-22
GO:0006814	Contraction	biological_process	sodium ion transport	40	2.15E-05
GO:0046872	Contraction	molecular_function	metal ion binding	28	7.87E-11
GO:0007156	Contraction	biological_process	homophilic cell adhesion via plasma membrane adhesion molecules	28	2.30E-03
GO:0006325	Contraction	biological_process	chromatin organization	27	1.20E-09
GO:0003774	Contraction	molecular_function	motor activity	25	3.51E-05
GO:0006357	Contraction	biological_process	regulation of transcription from RNA polymerase II promoter	23	8.07E-08
GO:0007601	Contraction	biological_process	visual perception	22	1.31E-10
GO:0006366	Contraction	biological_process	transcription from RNA polymerase II promoter	22	1.47E-02
GO:0016021	Contraction	cellular_component	integral component of membrane	21	6.44E-45
GO:0006511	Contraction	biological_process	ubiquitin-dependent protein catabolic process	21	4.74E-21

GO:0050776	Contraction	biological_process	regulation of immune response	21	3.40E-06
GO:0007283	Contraction	biological_process	spermatogenesis	18	5.81E-14
GO:0016032	Contraction	biological_process	viral process	18	1.94E-13
GO:0007525	Contraction	biological_process	somatic muscle development	18	6.82E-09
GO:0016567	Contraction	biological_process	protein ubiquitination	14	6.58E-08
GO:0045944	Contraction	biological_process	positive regulation of transcription from RNA polymerase II promoter	13	4.01E-08
GO:0008270	Contraction	molecular_function	zinc ion binding	13	2.00E-04
GO:0016192	Contraction	biological_process	vesicle-mediated transport	11	4.28E-13
GO:0030335	Contraction	biological_process	positive regulation of cell migration	11	1.31E-04
GO:0045893	Contraction	biological_process	positive regulation of transcription, DNA-templated	11	3.24E-04
GO:0048741	Contraction	biological_process	skeletal muscle fiber development	11	1.04E-03
GO:0051225	Contraction	biological_process	spindle assembly	11	1.44E-02
GO:0001916	Contraction	biological_process	positive regulation of T cell mediated cytotoxicity	10	6.15E-08
GO:0061408	Contraction	biological_process	positive regulation of transcription from RNA polymerase II promoter in response to heat stress	10	5.71E-05
GO:0051321	Contraction	biological_process	meiotic cell cycle	10	2.07E-04
GO:0006351	Contraction	biological_process	transcription, DNA-templated	10	7.42E-04
GO:0061097	Contraction	biological_process	regulation of protein tyrosine kinase activity	9	3.68E-10
GO:0005200	Contraction	molecular_function	structural constituent of cytoskeleton	9	8.33E-04
GO:0005509	Contraction	molecular_function	calcium ion binding	9	5.34E-03
GO:0006468	Contraction	biological_process	protein phosphorylation	9	2.06E-02
GO:0060236	Contraction	biological_process	regulation of mitotic spindle organization	8	6.74E-03
GO:0006068	Contraction	biological_process	ethanol catabolic process	7	4.69E-08
GO:0007140	Contraction	biological_process	male meiosis	7	4.69E-08
GO:0005634	Contraction	cellular_component	nucleus	7	1.15E-05

GO:0051492	Contraction	biological_process	regulation of stress fiber assembly	7	2.32E-04
GO:0007169	Contraction	biological_process	transmembrane receptor protein tyrosine kinase signaling pathway	7	6.96E-04
GO:0009617	Contraction	biological_process	response to bacterium	7	2.14E-03
GO:0006412	Contraction	biological_process	translation	6	1.28E-13
GO:0045599	Contraction	biological_process	negative regulation of fat cell differentiation	6	1.53E-04
GO:0000122	Contraction	biological_process	negative regulation of transcription from RNA polymerase II promoter	6	2.69E-04
GO:0006890	Contraction	biological_process	retrograde vesicle-mediated transport, Golgi to ER	6	7.43E-03
GO:0001570	Contraction	biological_process	vasculogenesis	6	1.52E-02
GO:0003016	Contraction	biological_process	respiratory system process	5	5.91E-06
GO:0045429	Contraction	biological_process	positive regulation of nitric oxide biosynthetic process	5	5.91E-06
GO:0007188	Contraction	biological_process	adenylate cyclase-modulating G-protein coupled receptor signaling pathway	5	5.91E-06
GO:0010814	Contraction	biological_process	substance P catabolic process	5	5.91E-06
GO:0030850	Contraction	biological_process	prostate gland development	5	5.91E-06
GO:0031098	Contraction	biological_process	stress-activated protein kinase signaling cascade	5	3.34E-04
GO:0046883	Contraction	biological_process	regulation of hormone secretion	5	9.33E-04
GO:0035751	Contraction	biological_process	regulation of lysosomal lumen pH	5	9.33E-04
GO:0006776	Contraction	biological_process	vitamin A metabolic process	5	9.33E-04
GO:0051493	Contraction	biological_process	regulation of cytoskeleton organization	5	9.33E-04
GO:0009651	Contraction	biological_process	response to salt stress	5	9.33E-04
GO:0005978	Contraction	biological_process	glycogen biosynthetic process	5	6.96E-03
GO:0050871	Contraction	biological_process	positive regulation of B cell activation	5	1.19E-02
GO:0050852	Contraction	biological_process	T cell receptor signaling pathway	4	1.04E-06
GO:0006979	Contraction	biological_process	response to oxidative stress	4	2.35E-05

GO:0040020	Contraction	biological_process	regulation of meiotic nuclear division	4	6.60E-05
GO:0051787	Contraction	molecular_function	misfolded protein binding	4	6.60E-05
GO:0045663	Contraction	biological_process	positive regulation of myoblast differentiation	4	6.60E-05
GO:0006434	Contraction	biological_process	seryl-tRNA aminoacylation	4	6.60E-05
GO:0016486	Contraction	biological_process	peptide hormone processing	4	6.60E-05
GO:0061563	Contraction	biological_process	trigeminal ganglion structural organization	4	6.60E-05
GO:0004714	Contraction	molecular_function	transmembrane receptor protein tyrosine kinase activity	4	6.60E-05
GO:0060285	Contraction	biological_process	cilium-dependent cell motility	4	3.01E-04
GO:0042147	Contraction	biological_process	retrograde transport, endosome to Golgi	4	6.75E-04
GO:0061630	Contraction	molecular_function	ubiquitin protein ligase activity	4	5.21E-03
GO:0006556	Contraction	biological_process	S-adenosylmethionine biosynthetic process	4	5.21E-03
GO:0007214	Contraction	biological_process	gamma-aminobutyric acid signaling pathway	4	5.21E-03
GO:0007522	Contraction	biological_process	visceral muscle development	4	5.21E-03
GO:0050690	Contraction	biological_process	regulation of defense response to virus by virus	4	5.21E-03
GO:0007286	Contraction	biological_process	spermatid development	4	1.48E-02
GO:0016055	Contraction	biological_process	Wnt signaling pathway	3	1.80E-28
GO:0015031	Contraction	biological_process	protein transport	3	1.02E-22
GO:0008380	Contraction	biological_process	RNA splicing	3	1.02E-13
GO:0006508	Contraction	biological_process	proteolysis	3	7.27E-13
GO:0005886	Contraction	cellular_component	plasma membrane	3	3.03E-08
GO:0055085	Contraction	biological_process	transmembrane transport	3	1.27E-07
GO:0007275	Contraction	biological_process	multicellular organismal development	3	1.39E-07
GO:0006367	Contraction	biological_process	transcription initiation from RNA polymerase II promoter	3	5.34E-06
GO:0006477	Contraction	biological_process	protein sulfation	3	7.36E-04

GO:0030201	Contraction	biological_process	heparan sulfate proteoglycan metabolic process	3	7.36E-04
GO:0072513	Contraction	biological_process	positive regulation of secondary heart field cardioblast proliferation	3	7.36E-04
GO:0030687	Contraction	cellular_component	preribosome, large subunit precursor	3	7.36E-04
GO:2000045	Contraction	biological_process	regulation of G1/S transition of mitotic cell cycle	3	7.36E-04
GO:0060223	Contraction	biological_process	retinal rod cell fate commitment	3	7.36E-04
GO:0046889	Contraction	biological_process	positive regulation of lipid biosynthetic process	3	7.36E-04
GO:0003420	Contraction	biological_process	regulation of growth plate cartilage chondrocyte proliferation	3	7.36E-04
GO:0072321	Contraction	biological_process	chaperone-mediated protein transport	3	7.36E-04
GO:0043204	Contraction	cellular_component	perikaryon	3	7.36E-04
GO:0030195	Contraction	biological_process	negative regulation of blood coagulation	3	7.36E-04
GO:0001164	Contraction	molecular_function	RNA polymerase I CORE element sequence-specific DNA binding	3	7.36E-04
GO:0070508	Contraction	biological_process	cholesterol import	3	7.36E-04
GO:0060535	Contraction	biological_process	trachea cartilage morphogenesis	3	7.36E-04
GO:0042823	Contraction	biological_process	pyridoxal phosphate biosynthetic process	3	7.36E-04
GO:0045725	Contraction	biological_process	positive regulation of glycogen biosynthetic process	3	7.36E-04
GO:0008154	Contraction	biological_process	actin polymerization or depolymerization	3	7.36E-04
GO:0021722	Contraction	biological_process	superior olivary nucleus maturation	3	7.36E-04
GO:0071731	Contraction	biological_process	response to nitric oxide	3	7.36E-04
GO:0010765	Contraction	biological_process	positive regulation of sodium ion transport	3	7.36E-04
GO:0070863	Contraction	biological_process	positive regulation of protein exit from endoplasmic reticulum	3	7.36E-04
GO:0005355	Contraction	molecular_function	glucose transmembrane transporter activity	3	7.36E-04
GO:0050772	Contraction	biological_process	positive regulation of axonogenesis	3	7.36E-04

GO:0033209	Contraction	biological_process	tumor necrosis factor-mediated signaling pathway	3	1.36E-03
GO:0015887	Contraction	biological_process	pantothenate transmembrane transport	3	6.10E-03
GO:0042136	Contraction	biological_process	neurotransmitter biosynthetic process	3	6.10E-03
GO:0008284	Contraction	biological_process	positive regulation of cell proliferation	3	7.07E-03
GO:0006368	Contraction	biological_process	transcription elongation from RNA polymerase II promoter	3	8.11E-03
GO:1901998	Contraction	biological_process	toxin transport	3	9.29E-03
GO:0006915	Contraction	biological_process	apoptotic process	3	1.48E-02
GO:1990440	Contraction	biological_process	positive regulation of transcription from RNA polymerase II promoter in response to endoplasmic reticulum stress	3	1.77E-02
GO:0032496	Contraction	biological_process	response to lipopolysaccharide	3	1.79E-02
GO:0010468	Contraction	biological_process	regulation of gene expression	3	2.04E-02
GO:0005576	Contraction	cellular_component	extracellular region	2	1.63E-08
GO:0007399	Contraction	biological_process	nervous system development	2	4.22E-06
GO:0048511	Contraction	biological_process	rhythmic process	2	2.14E-03
GO:0010092	Contraction	biological_process	specification of organ identity	2	8.17E-03
GO:1903818	Contraction	biological_process	positive regulation of voltage-gated potassium channel activity	2	8.17E-03
GO:0015826	Contraction	biological_process	threonine transport	2	8.17E-03
GO:0097191	Contraction	biological_process	extrinsic apoptotic signaling pathway	2	8.17E-03
GO:0006505	Contraction	biological_process	GPI anchor metabolic process	2	8.17E-03
GO:0072560	Contraction	biological_process	type B pancreatic cell maturation	2	8.17E-03
GO:0016322	Contraction	biological_process	neuron remodeling	2	8.17E-03
GO:0060976	Contraction	biological_process	coronary vasculature development	2	8.17E-03
GO:0035249	Contraction	biological_process	synaptic transmission, glutamatergic	2	1.41E-02

Supplementary Table 26. Thermal niche overlap between banded penguin species.

Species Pair	niche expansion Exclusive niche sp2 (not shared with 2)	niche stability (shared Niche Of 1 with 2)	Niche unfilling (unoccupied niche of sp1 by sp2)	Salinity D overlap
Galápagos-Magellanic	0.988	0.012	0.002	0.041
Galápagos-Humboldt	0.996	0.004	0.861	0.014
Magellanic-Humboldt	0.001	0.999	0.374	0.555
Humboldt-African	0.005	0.995	0.028	0.568
Magellanic-African	0	1	0.012	0.374
Galápagos-African	0.993	0.007	0.717	0.014

Supplementary Table 27. Chlorophyll niche overlap between banded penguin species.

Species Pair	niche expansion Exclusive niche sp2 (not shared with 2)	niche stability (shared Niche Of 1 with 2)	Niche unfilling (unoccupied niche of sp1 by sp2)	Salinity D overlap
Galápagos-Magellanic	0.151	0.849	0.000	0.444
Galápagos-Humboldt	0.610	0.390	0.216	0.256
Magellanic-Humboldt	0	1	0.195	0.742

Humboldt-African	0	1	0.005	0.633
Magellanic-African	0	1	0.107	0.698
Galápagos-African	0.146	0.854	0.022	0.382

Supplementary Table 28. Salinity niche overlap between banded penguin species.

Species Pair	niche expansion Exclusive niche sp2 (not shared with 2)	niche stability (shared Niche Of 1 with 2)	Niche unfilling (unoccupied niche of sp1 by sp2)	Salinity D overlap
Galápagos-Magellanic	0	1	0	0.016
Galápagos-Humboldt	0.715	0.285	0	0.037
Magellanic-Humboldt	0	1	0.118	0.500
Humboldt-African	0.33	0.67	0.489	0.060
Magellanic-African	0	1	0.202	0.059
Galápagos-African	0.965	0.035	0.289	0.312

Supplementary Table 29. Overall niche overlap between banded penguin species.

Species	Galápagos	Magellanic	Humboldt	African
Galápagos		0.007037	0.100545	0.04792
Magellanic			0.530628	0.183882
Humboldt				0.324413
African				

Supplementary Table 30. Overall and univariate niche overlap between genetic populations of banded penguin species.

	Niche Overlap	Temp D	Salinity D	Chlorophyll
S. mendilicus (Isabella-Santiago)	0.05610183	0.11819	0.39965	0.06557
S. humboldti (North - South)	0.0909311	0.00691	0.03415	0.65676
S. magellanicus (Pacific - Atlantic)	0.3954385	0.4926	0.41261	0.69899

Supplementary Table 31. Dynamic niche evolution between genetic populations of banded penguin species. Niche expansion / stability / unfilling is given as comparison of pair 2 to pair 1.

**Galápagos penguin**	**Expansion**	**Stability**	**Unfilling**
Temperature	0.02435257	0.97564743	0.69061723
Salinity	0.002850638	0.997149362	0.388978330
Chlorophyll	0.000000	1.000000	0.523524
**Humboldt Penguin**	**Expansion**	**Stability**	**Unfilling**
Temperature	0.995253235	0.004746765	0.994453671
Salinity	0.7653543	0.2346457	0.7746007
Chlorophyll	0.00000000	1.00000000	0.04767163
**Magellanicus Penguin**	**Expansion**	**Stability**	**Unfilling**
Temperature	0.323249127	0.676750873	0.006495807

Salinity	0.69485188	0.30514812	0.06332115
Chlorophyll	0.34090898	0.65909102	0.02369917

Supplementary Table 32. **Significant bioclimatic variables for RDA methods.**

Variable	Acronym
Average speed of present ocean currents	VELO
Annual Mean Temperature	BIO1
Chlorophyll	CHLO
Annual Mean Precipitation	BIO12
Mean Diurnal Range	BIO2

Supplementary Table 33. Redundancy Analysis (RDA) statistic significant for autosomal Single Nucleotide Polymorphisms (SNPs) of banded penguin Species. The R-squared values explained variance and the adjusted R-squared values account for predictor influence. Both sets exhibit significant F-statistics underscoring the importance of genetic variations in influencing the observed outcomes.

Species	R^2	R^2 Ajusted	Eigenvalues (RDA1)	Eigenvalues (RDA2)	Proportion explain (RDA1)	Proportion explain (RDA2)	Pr(>F)
**All Species**	0.166	0.135	568.5569	175.7189	0.6832	0.2111	0.001
**Gal**	0.107	0.008	231.1567	207.246	0.5273	0.4727	0.006
**Hum**	0.128	0.003	156.2886	144.2786	0.2731	0.2521	0.096
**Mag**	0.121	0.004	155.6432	141.6952	0.2756	0.2509	0.017
**Afr**	0.201	0.001	273.4021	219.2992	0.3072	0.2464	0.092
